# Structure-Antitumor Activity Relationships of Aza- and Diaza-Anthracene-2,9,10-Triones and Their Partially Saturated Derivatives

**DOI:** 10.3390/molecules29020489

**Published:** 2024-01-18

**Authors:** Carmen Avendaño, Pilar López-Alvarado, José María Pérez, Miguel Ángel Alonso, Eva Pascual-Alfonso, Miriam Ruiz-Serrano, J. Carlos Menéndez

**Affiliations:** Unidad de Química Orgánica y Farmacéutica, Departamento de Química en Ciencias Farmacéuticas, Facultad de Farmacia, Universidad Complutense, 28040 Madrid, Spain; alvarado@ucm.es (P.L.-A.); jmpnunez@gmail.con (J.M.P.); alonrizal@hotmail.com (M.Á.A.); epascual24@hotmail.com (E.P.-A.); miriamruizserrano@ucm.es (M.R.-S.)

**Keywords:** azaanthracene-2,9,10-triones, diazaanthracene-2,9,10-triones, hetero Diels–Alder reactions, antitumor activity, diazaquinomycins, marcanines

## Abstract

The 1,8-Diazaanthracene-2,9,10-triones, their 5,8-dihydro derivatives, and 1,8-diazaanthracene-2,7,9,10-tetraones, structurally related to the diazaquinomycin family of natural products, were synthesized in a regioselective fashion employing Diels–Alder strategies. These libraries were studied for their cytotoxicity in a variety of human cancer cell lines in order to establish structure–activity relationships. From the results obtained, we conclude that some representatives of the 1,8-diazaanthracene-2,9,10-trione framework show potent and selective cytotoxicity against solid tumors. Similar findings were made for the related 1-azaanthracene-2,9,10-trione derivatives, structurally similar to the marcanine natural products, which showed improved activity over their natural counterparts. An enantioselective protocol based on the use of a SAMP-related chiral auxiliary derived was developed for the case of chiral 5-substituted 1,8-diazaanthracene-2,9,10-triones, and showed that their cytotoxicity was not enantiospecific.

## 1. Introduction

Natural products containing a 9,10-anthracenedione substructure are well known and have been widely studied as dyes [1] and for their anticancer activity [2], among other properties. They include antitumour compounds such as the anthracyclines [3,4,5], the pluramycins [6,7,8] and some of the enediyne antibiotics [9,10]. On the other hand, their hetero analogues are less common. We have focused on the synthesis and study of analogues of less known structures such as 1-aza- and 1,8-diaza-9,10-anthracenediones [11,12].

**Figure 1:**
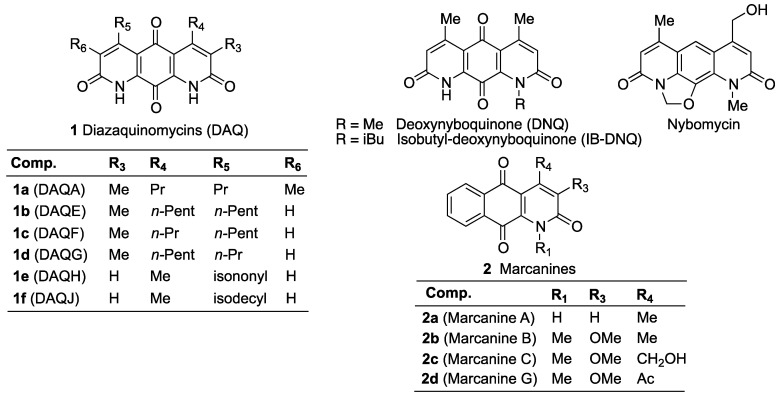
Structures of the diazaquinomycins, deoxynyboquinones, and marcanines.

The deoxyniboquinones [13,14] and the diazaquinomycins (DAQs) are the only natural products derived from the 1,8-diazaanthracene-2,7,9,10-trione framework, although some related natural products, like nybomycin, are known [15,16]. After its isolation from a *Streptomyces* strain, the Ōmura group found that diazaquinomycin A (DAQA) possessed antibacterial properties [17,18], which they subsequently proposed to be due to its ability to inhibit thimidylate synthase [19]. Both findings made DAQA an attractive lead compound in the field of cancer chemotherapy; although the natural product itself lacked useful antitumor activity because of its poor pharmacokinetic properties [20]. On the other hand, subsequent studies by Murphy that involved the screening of an actinomycete secondary metabolite library against an ovarian cancer cell line led to the identification of the diazaquinomycins E, F and G [21], and a similar study by the same group of a library of aquatic bacterial natural product fractions led to the discovery of diazoquinomycins H and J, and also a promising in vitro activity profile for some DAQs against a panel of drug-resistant *Mycobacterium tuberculosis* strains [22] (Figure 1, compounds **1**). Interestingly, diazaquinomycin biosynthetic gene clusters have been sequenced from marine and freshwater actinomycetes [23].

The structurally related 1-azaanthracenones are another class of natural compounds with interesting anticancer activity. In 1999, Soonthornchareonnon et al. described the cytotoxic activity of marcanine A, isolated from the stem bark of *Goniothalaus marcanii* [24], and its derivatives [25] against several tumor cell lines (Figure 1, compounds **2**). Subsequently, these compounds have also been found to exhibit antimalarial activity against *Plasmodium falciparum* [26,27]. Jacobs et al. evaluated the cytotoxic activity of a series of compounds with an azaanthracenedione structure, finding that the quinone scaffold and the lactam moiety are important for activity [28]. Their mechanism of action is not clear, although some marcanine derivatives have been found to induce apoptosis via caspase-3 activation [29].

**Figure 2:**
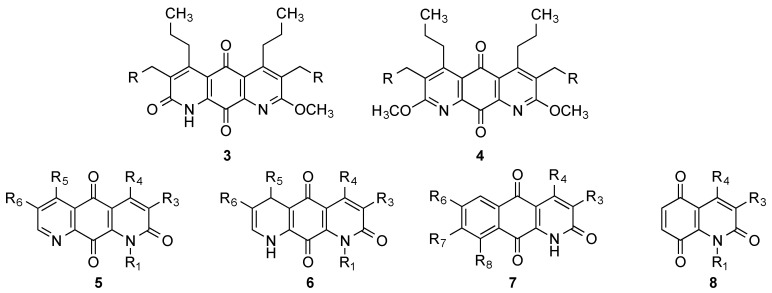
Diazaquinomycin derivatives prepared by Ōmura (**3** and **4**) and compounds **5**, **6**, **7**, and **8** studied here.

To date, there are no conclusive data about structure–activity relationships for diazoquinomycin analogues. The preparation and study of a number of semisynthetic analogues of DAQA (compounds **3** and **4**) led Ōmura to the conclusion that the bis-lactam structure of the natural product was an essential requisite for thimidylate synthase inhibition and antitumor activity [30]. However, when we investigated the antitumor activity of a small set of derivatives of the structure **5**, we found that they exhibited good antitumor activities, particularly towards solid tumors [31], while the introduction of a fluorine atom at C-3 led to compounds with antileukemic activity [32]. In order to assist the establishment of structure–activity relationships within this class of compounds, we describe here a systematic study of the in vitro antitumor properties of a large series of derivatives of structure **5**, their 5,8-dihydro derivatives **6**, where one of the electron-withdrawing pyridine nitrogen atoms has been replaced by an electron-releasing enamine-like moiety, 1-azaanthraquinones derived from structure **7** related to the marcanines and more simplified structures such as quinolinedione derivatives **8** where the lactam scaffold that appears to be essential for cytotoxic activity is maintained (Figure 2).

## 2. Results and Discussion

### 2.1. Preparation of 1,8-Diazaanthracene-2,9,10-trione Derivatives

Compounds **5** and **6** were prepared using hetero Diels–Alder chemistry [33,34,35,36,37]. The starting materials were, on the one hand, the unsaturated dimethylhydrazones **9**, obtained [38,39] from *N*,*N*-dimethylhydrazine and the corresponding aldehydes, which are commercially available with the exception of 2-methyl-2-hexenal, synthesized using a literature procedure [40] (Table 1). On the other hand, the quinones **8** acting as dienophiles were obtained from simple aromatic starting materials using literature methods based on the Knorr [41,42,43,44] and Meth–Cohn [45] quinoline syntheses, followed by an oxidative demethylation reaction (Table 2).

**Scheme 1:**
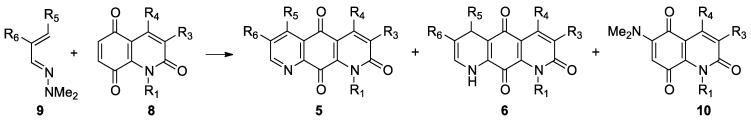
Hetero Diels–Alder reaction between azadienes **9** and quinones **8**.

The course of the reaction between compounds **9** and **8** depended on their substituent distribution (Scheme 1); thus, combinations of hydrazones **9** and quinones **8** (R_4_, R_5_ ≠ H) gave 5,8-dihydro-1,8-diazaanthraquinones **6** as the sole cycloadducts, while other combinations gave the fully aromatic compounds **5** [33,46] with the exception of the reaction between azadiene **9b** and quinone **8h**, which, together with **5p**, gave a significant amount of the corresponding dihydro derivative **6p** that could not be isolated in pure form. In this particular case, the reaction crude was treated with manganese dioxide to force the dehydrogenation of **6p**. As a secondary product of the reaction, compound **10** was obtained from the addition of dimethylamine to the starting quinones **8**, followed by spontaneous air oxidation (Table 3, method A). These side products were formed in a higher yield when dimethylhydrazone derivatives of crotonaldehyde were used; in these cases, a method previously described by our group based on the use of a silica gel support [34] allowed us to considerably reduce the formation of **10** (Table 3, method B). Alternatively, some of the reactions were performed in the presence of a chloroformyl polystyrene resin in order to trap the liberated dimethylamine [35] (Table 3, method C).

**Scheme 2:**
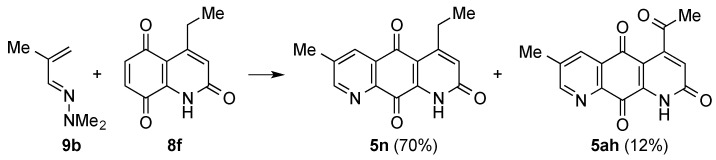
The hetero Diels–Alder reaction of azadiene **9b** and quinone **8f**.

The reaction between azadiene **9b** and quinone **8f** afforded compound **5n** as the main reaction product, together with a small amount of a derivative oxidized at the C_4_ benzylic position (**5ah**) (Scheme 2).

**Scheme 3:**
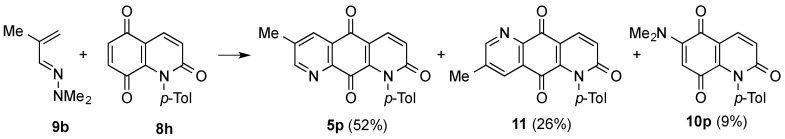
A mixture of 1,5-diaza- and 1,8-diaza-regioisomers was isolated in the reaction of the N-aryl quinolinetrione **8h** with azadiene **9b**.

The hetero Diels–Alder reactions performed were usually regioselective, according to the previous experience of our group in similar reactions [33,46], and afforded only the 1,8-diazanthracene-2,9,10-trione structure. The selectivity of the process towards the C_6_ end can be attributed to the combination of two electronic factors: the conjugation of the carbonyl at C-5 with the lactam nitrogen and the deficiency created at C_8_=O as a consequence of its conjugation with the C_2_=O group and the existence of an intramolecular hydrogen bond with N_1_-H [33]. In one case, as shown in Scheme 3, the reaction of 1-(4-tolyl)-1-*H*-2,5,8-quinolinetrione (**8h**) with azadiene **9b** afforded significant amounts of the regioisomer 1,5-diaza adduct **11** (**5p**:**11** = 2:1). In this case, the presence of an aryl group on N_1_ of the quinone may alter the first effect mentioned above because of the partial transfer of the nitrogen charge towards the aromatic ring; on the other hand, the presence of a substituent on N_1_ may affect the coplanarity of the system, hindering the conjugative effects described above and also leading to the loss of the N_1_-H bond.

**Scheme 4:**
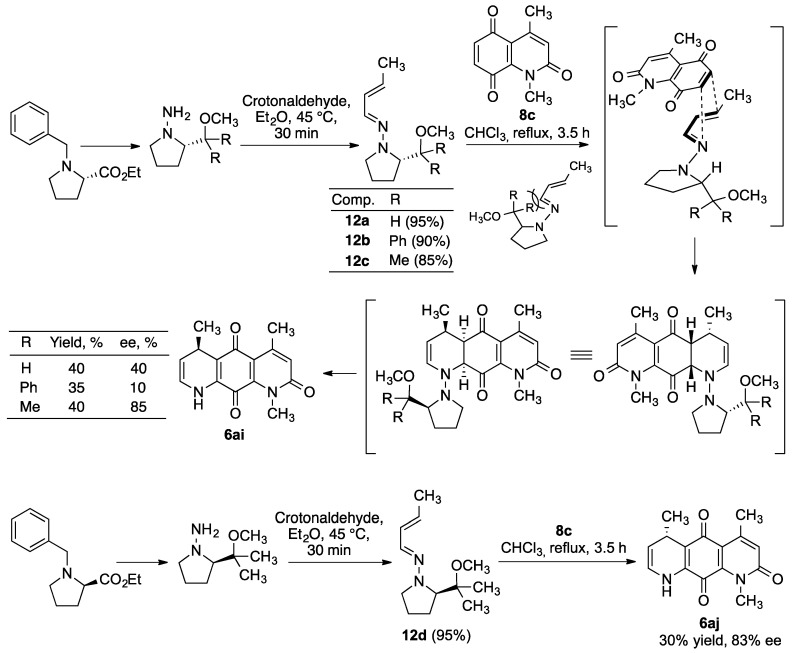
Synthesis of enantio-enriched compound **6ai** and its enantiomer **6aj**. The chiral 1-aminopyrrolidine derivatives were obtained according to reference [47].

The reaction of C_4_-substituted 1-dimethylamino-1-azadienes affords C_5_-substituted 5,8-dihydro-1,8-diazaanthracene-2,9,10-trione systems, which are chiral and contain a stereogenic center at C_5_. It was considered of interest to obtain one of the compounds of this series in enantiomerically pure or enriched form in order to evaluate the stereospecificity of the antitumor activity. To achieve asymmetric induction in the Diels–Alder reaction, it was decided to employ chiral auxiliaries attached to the azadiene structure, which were synthetized from chiral 1-aminopyrrolidine derivatives obtained from (*S*)-*N*-benzyl ethyl prolinate and (*R*)-*N*-benzyl ethyl prolinate using a route described in the literature [47]. The only chiral dimethylamino-1-azadiene previously studied as a Diels–Alder diene is an analogue of compound **12a**, containing an additional methyl at C-2 and derived from the Enders SAMP chiral auxiliary, which gave cycloadducts with maleic anhydride and maleimide derivatives in 76–98% enantiomeric excess (ee) [48]. Therefore, the reaction of **12a** with quinone **8c** was employed for optimizing our process. After some unsuccessful initial attempts at room temperature, we found that the reaction between **12a** and **8c** in refluxing chloroform for 3.5 h gave the desired adduct **6ai** although in moderate yield (40%) and enantioselectivity (40% ee). The more hindered diphenyl diene **12b** also gave poor results, but its dimethyl analogue **12c** afforded **6ai** in 40% yield and 85% ee. Similarly, the reaction of quinone **8c** with **12d**, the enantiomer of **12c**, under the same conditions gave **6aj** in 30% yield and 83% ee. The configuration of the C_5_ stereocenter in these compounds was proposed according to the *endo* transition state shown in Scheme 4, according to a literature proposal for this type of azadienes [48]. The level of stereoselection achieved with azadienes **12c** and **12d** was considered enough for a comparison of the activities of enantiomeric derivatives of the structure **6**.

**Scheme 5:**
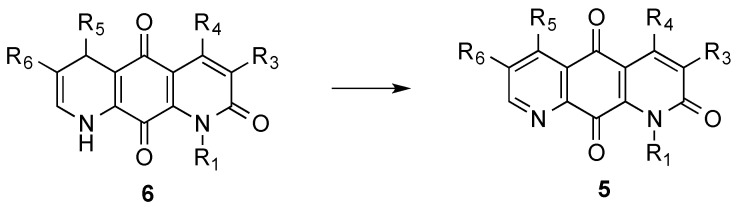
Dehydrogenation of dihydro derivatives **6**.

In order to complete the library of compounds **5**, the dehydrogenation of their dihydro derivatives **6** (Scheme 5) was examined by comparing two methods, namely treatment with manganese dioxide (method A), which afforded good to excellent yields under mild conditions and air oxidation at high temperature (method B), which was efficient but rather slow. In the case of compound **5r**, air oxidation was carried out in the presence of NaOH at room temperature (method C) (Table 4).

### 2.2. Preparation of 1-Azaanthracene-2,9,10-trione Derivatives ***7***

**Scheme 6:**
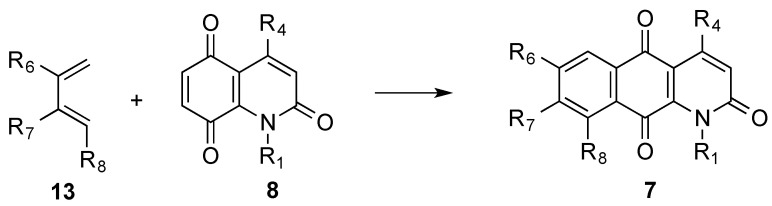
Diels–Alder reactions of dienes **13** and quinones **8**.

The deaza analogues **7** of the previously obtained compounds were prepared using standard Diels–Alder chemistry from quinones **8** and dienes **13** using thermal reaction conditions in sealed tubes [49] (Scheme 6 and Table 5). Compound **7f** is the natural product marcanine A [24].

**Scheme 7:**
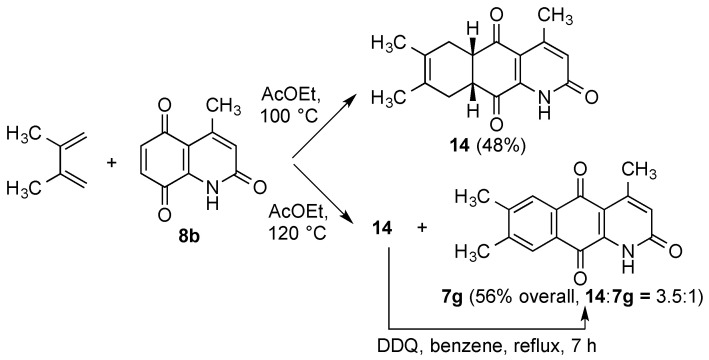
Synthesis of the 1-azaanthracenetrione derivative **7g**.

In some cases, it was not possible to obtain the aromatic compound **7** in a single step, although the level of oxidation of the obtained cycloadducts could be controlled by modifying the reaction conditions. Thus, the reaction between quinone **8b** and 2,3-dimethyl-1,3-butadiene in ethyl acetate at 100 °C led to the formation of the primary Diels–Alder adduct **14**. The reaction in the same solvent under increased reaction temperature (120 °C) gave a mixture of the aromatic derivative **7g** and **14**, which by reaction with DDQ in benzene at reflux for 7 h finally afforded the target compound **7g** as a single product in 56% overall yield (Scheme 7).

**Scheme 8:**
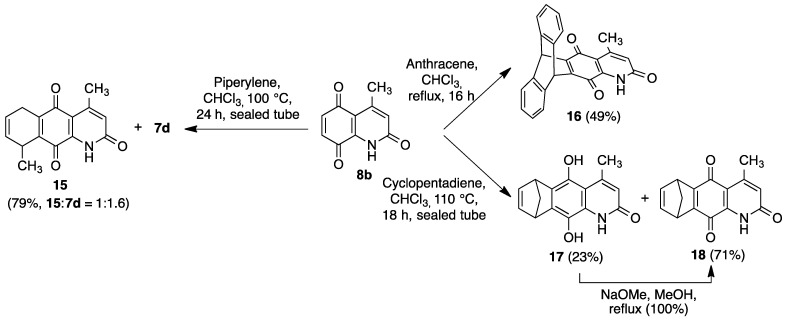
Synthesis of 5,8-dihydro-1-azaanthracenetrione derivatives.

In order to study the activity of some 5,8-dihydro derivatives of the 1-azaanthracenetrione framework, we resorted to modulating the reaction conditions to prevent the full aromatization of the primary Diels–Alder adduct. Thus, compound **15** was obtained, together with the corresponding aromatic derivative **7d**, as a minor product of the reaction of quinone **8b** with piperylene (1,3-pentadiene) in chloroform at 100 °C in a sealed tube for 24 h. Additional 5,8-dihydro-1-azaanthracenetrione derivatives were obtained using the diene anthracene (compound **16**) and cyclopentadiene. In the latter case, the target compound **18** was obtained together with the corresponding hydroquinone **17**, but this mixture was transformed into **18** by a simple reflux in methanolic sodium hydroxide (Scheme 8).

### 2.3. Biological Studies

**Figure 3:**
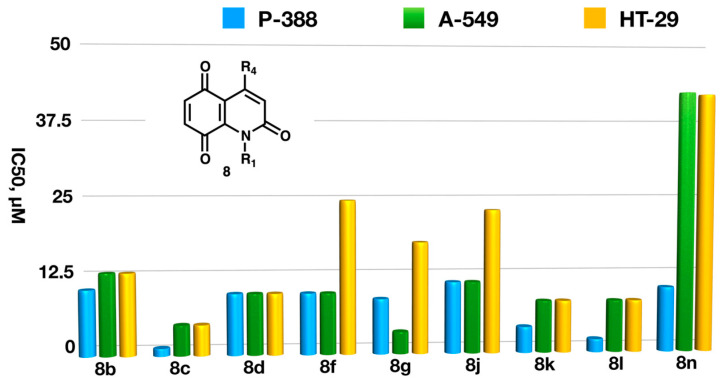
Cytotoxicity of quinolinetriones **8**. Numerical data can be found in Table S1 (Supporting Information).

**Figure 4:**
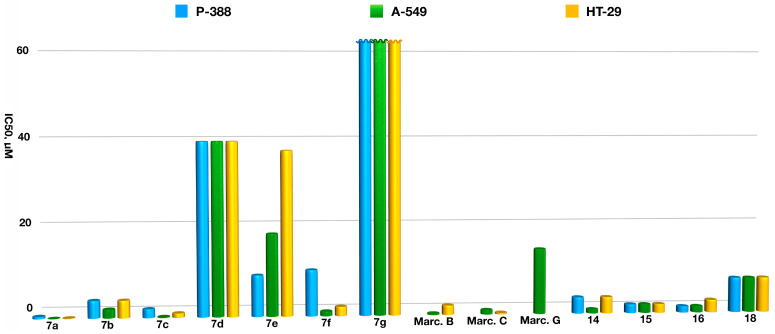
Cytotoxicity of 1-azaanthracenetrione derivatives **7**, the related non-aromatic intermediate **14** and 5,8-dihydro-1-azaanthracenetrione derivatives **15**, **16**, and **18**. Numerical data can be found in Tables S2 and S3 (Supporting Information).

**Figure 5:**
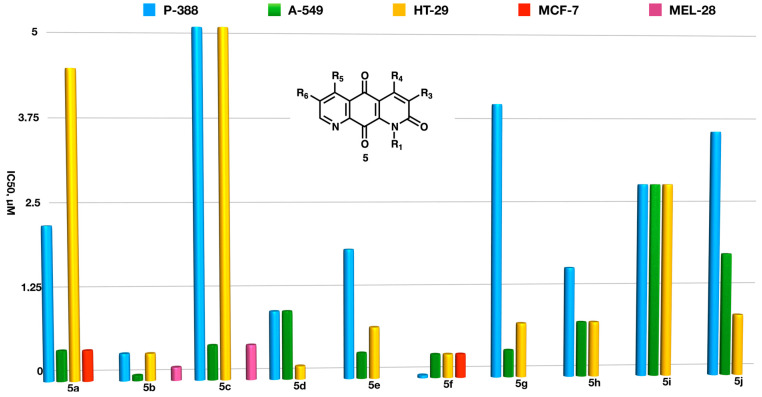
Cytotoxicity of 1,8-diazaanthracenetriones **5a**–**5j**. Numerical data can be found in Table S4 (Supporting Information).

**Figure 6:**
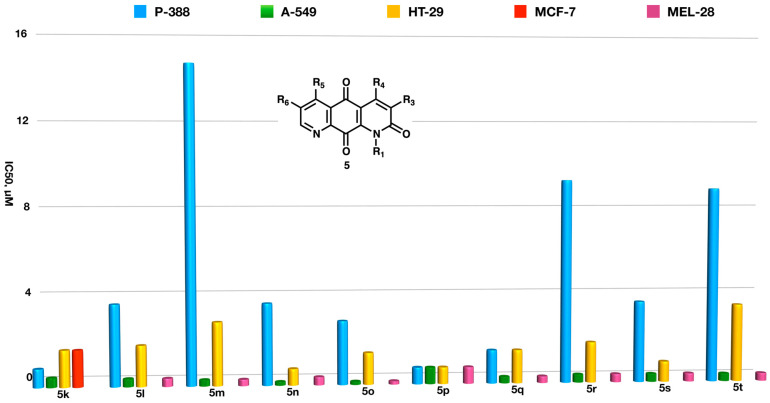
Cytotoxicity of 1,8-diazaanthracenetriones **5k**–**5t**. Numerical data can be found in Table S4 (Supporting Information).

**Figure 7:**
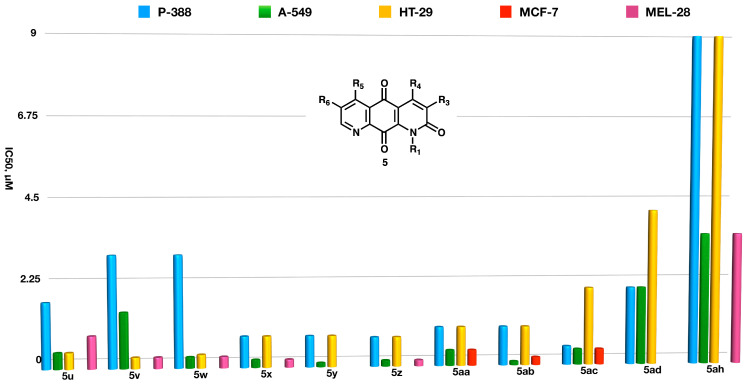
Cytotoxicity of 1,8-diazaanthracenetriones **5u**–**5ah**. Numerical data can be found in Table S4 (Supporting Information).

**Figure 8:**
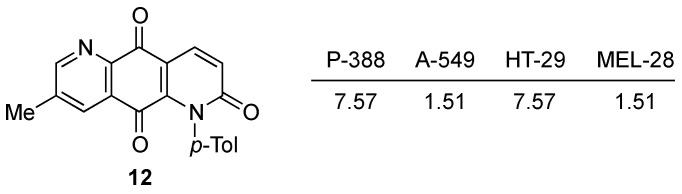
Cytotoxicity of compound **12** (IC_50_, µM).

**Figure 9:**
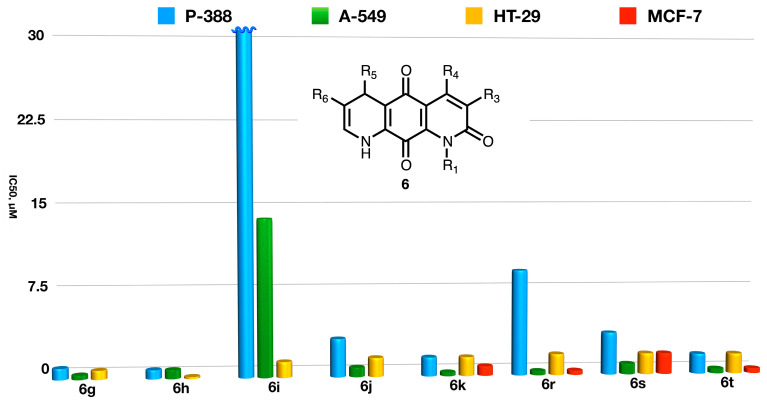
Cytotoxicity of 5,8-dihydro-1,8-diazaanthracenetriones **6g**–**6t**. Numerical data can be found in Table S5 (Supporting Information).

**Figure 10:**
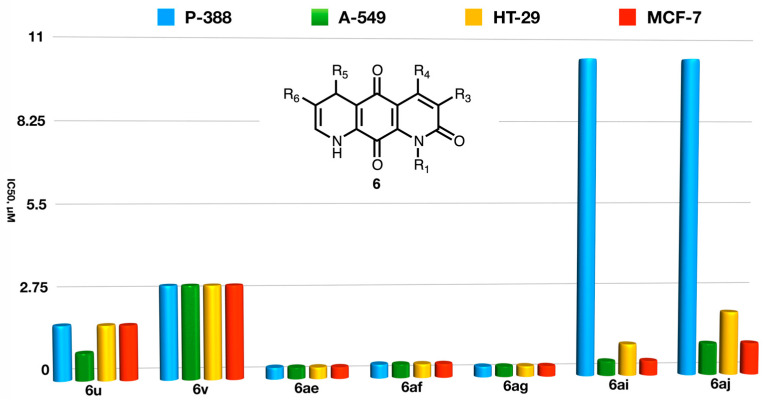
Cytotoxicity of 5,8-dihydro-1,8-diazaanthracenetriones **6u**–**6aj**. Numerical data can be found in Table S5 (Supporting Information).

**Figure 11:**
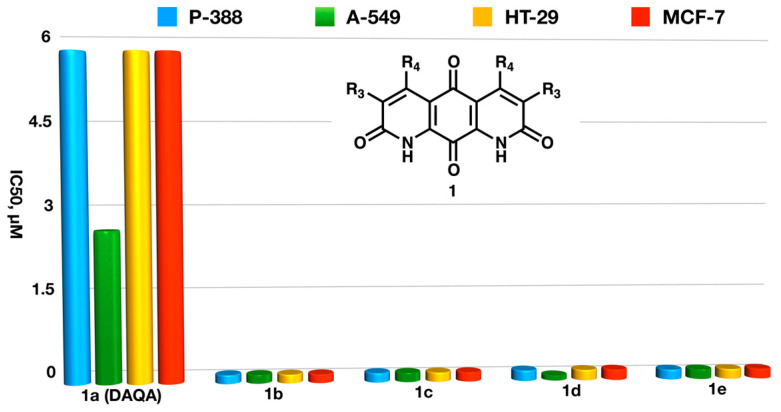
Cytotoxicity of diazaaquinomycins **1**. Numerical data can be found in Table S6 (Supporting Information).

The in vitro cytotoxicity of compounds derived from the structures **5**, **6**, **7**, and **8** was studied in the following cell lines: mouse lymphoma (P-388), human lung carcinoma (A-549), human colon carcinoma (HT-29), human breast carcinoma (MCF-7), and human melanoma (MEL-28). For comparison purposes, we also evaluated the activity of diazaquinomycin A and some related compounds derived from structure **1** in these cell lines to establish a structure–activity relationship as conclusive as possible and determine the pharmacophore group. The results are shown in Figure 3, Figure 4, Figure 5, Figure 6, Figure 7, Figure 8, Figure 9, Figure 10 and Figure 11 and Tables S1–S6 in the Supporting Information expressed as inhibitory concentration 50 values (IC_50_, µM).

We first assessed the activity of simplified structures derived from the quinoline-2,5,8(1*H*)-trione framework (starting quinones **8**), with the results shown in Figure 3 and Table S1. These compounds generally showed relatively modest activities and selectivities towards the tumors assayed, although three of the compounds (**8c**, **8k** and **8l**) had IC_50_ values against the P-388 leukemia cell line at the micromolar level, with some selectivity with respect to solid tumors. Nevertheless, the comparison of these activities with those shown below for the Diels–Alder and Diels–Alder adducts reveals the need for the tricyclic system in order to achieve good anticancer activity.

The unsubstituted 1-azaanthracenetrione derivative **7a** showed the best antitumor activity of this series, and also selectivity against the lung cancer cell line, with an IC_50_ in the ten nanomolar range. Interestingly, this activity was superior to that of the marcanine B, C, and G natural products [24,25]. The presence of substituents at C_7_ and C_8_ considerably decreases their activity, as shown by the data for compounds **7d**–**7g**. It is also relevant to note that the primary Diels–Alder adduct showed a higher activity than its aromatic counterpart **7g** (Figure 4 and Table S2). The dihydro derivatives of the 1-azaanthracenetrione framework (compounds **15–18**) substituted at C_5_ and C_8_ also maintained the anticancer activity, although with low selectivity (Figure 4 and Table S3).

Regarding the activity of derivatives of structure **5** (Figure 5, Figure 6 and Figure 7 and Table S4), our observations lead to the following conclusions:

(a) Aromatic substituents at N-1 lower both activity and selectivity against solid tumors, as shown by the comparison of the data for compounds **5d** and **5p**.
(b) Aromatic substituents at C-3 do not improve the activity on solid tumor cell lines with respect to alkyl groups when directly attached to the anthraquinone ring (**5q** vs. **5l** and **5w** vs. **5r**)
(c) On the other hand, when an aromatic ring is attached to C_3_ via a methylidene spacer, not only is considerable antitumor activity observed, but also a notable selectivity towards certain solid tumors. Thus, compound **5m** is ten times more active in lung carcinoma and melanoma than in colon carcinoma and ca. 50 times more active than on the P-388 lymphoma cells. This difference also exists but is not so marked in the derivatives, which only present alkyl substituents at C_3_ such as **5l**.
(d) The presence of simultaneous substituents at C_3_, C_4_, and C_5_, leading to a steric interaction between the C_10_=O groups with R_4_ and R_4_, increases the activity against P-388 compared to the same compounds when they only have substituents at C_4_ and C_5_ or C_3_ and C_4_ (**5u** vs. **5t**).
(e) An increase in the length of the R_5_ chain seems to be accompanied by a slight decrease in activity in lung cancer cells (compounds **5g**, **5j**, and **5k**). Aromatic substituents, especially when bearing an electron-releasing group, also lead to decreased activity in these cells (compounds **5h** and **5i**).
(f) The presence of alkyl substituents at R_6_ is generally favorable for activity on the human colorectal adenocarcinoma cell line, as shown by the comparison of the data for **5c** and **5d**–**f**.
(g) Electron-withdrawing groups decrease the activity when they are in the C_3_ and C_4_ positions with respect to their alkyl counterparts, as shown by the comparison between the compounds (**5y**, **5z**, and **5aa** vs. **5ad** and **5ah vs. 5d**).

The 1,5-diazaanthracenetrione framework seems to provide less active compounds than its 1,8-diaza isomer when substituted at C-7, as shown by the comparison of the activity data of compounds **5p** and **12** (Figure 6 and Figure 8 and Table S4).

For 5,8-dihydro derivatives **6** (Figure 9 and Figure 10 and Table S5), the following conclusions were obtained:

(a) Generally speaking, the aromatic compounds **5** and their 5,8-dihydro counterparts **6** show similar activities, but some exceptions to this rule were observed, as summarized below.
(b) The presence of aromatic derivatives at C-5 increases the activity and selectivity against colon cancer cells in the dihydro derivatives such as **6h** and **6i** compared to their aromatized counterparts **5h** and **5i**. For N-aryl substitution, dihydro derivatives seem less selective for solid tumors (**5v** vs. **6v**).
(c) On the other hand, for the case of alkyl substitution the selectivity trend is the opposite one, with the aromatic systems **5** showing a higher selectivity for solid tumors than the corresponding compounds **6** (**5s** vs. **6s**, **5t** vs. **6t**, **5u** vs. **6u**). Even though a comparison cannot be established in these cases, the lack of selectivity found in compounds **6ae**, **6af**, and **6ag** supports this conclusion.
(d) A comparison of the activities of the enantio-enriched compounds **6ai** and **6aj** leads to the conclusion that the configuration of the stereogenic center at C-5 does not greatly influence the anti-tumor activity.

Diazaquinomycins have been synthesized using double Knorr reactions, a strategy developed by Kelly [50] and Sun [51], or hetero Diels–Alder chemistry followed by N-oxidation and rearrangement, as described by us [44]. Diazaquinomycin A (**1a**) thus prepared exhibited a lower activity and selectivity than their corresponding derivative with a monolactam system (**5u**) and its 5,8-dihydro derivative (**6u**). Furthermore, additional structures derived from the diazaquinomycin framework (compounds **1b**–**e**) showed a higher activity than the natural product but still lacked selectivity in their cytotoxicity (Figure 11 and Table S6).

**Figure 12:**
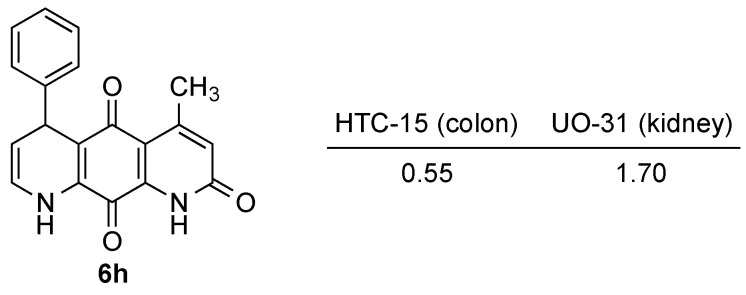
Activity of compound **6h** against MDR cell lines (IC_50_, μM).

Due to the importance of the multidrug resistance phenomenon (MDR) in cancer therapy, we also examined the effect of the representative compound **6h** on two cell lines showing multidrug resistance due to the overexpression of glycoprotein Pgp-170, namely the HTC-15 colon and the UO-21 kidney cancer lines. As shown in Figure 12, compound **6h** showed good activity in both cases.

Regarding the cytotoxicity mechanism of the compounds described here, compound **6h** gave negative results in the following potential anticancer targets: thymidylate synthase (the putative target of diazaquinomycin A [18,19]), dihydrofolate reductase, topoisomerases I and II, ADN polymerases and ARN polymerases. It also failed to block DNA, RNA, or protein synthesis. Moreover, no clear-cut connection can be established between the half-wave redox potential of the quinone moieties in our compounds [25] and their activities. Additional studies will be needed to gain insight into the mechanism of action of our compounds, and in this connection, studies on the ability of our compounds to block the cell cycle at a particular stage will perhaps be informative. Due to the facts that the formation of DNA-damaging anion-radicals often contributes to the anticancer activity of quinones [52,53] and that the deoxynyboquinones have been shown to generate reactive oxygen species via their activation by quinone reductase NQO1 [14], this possibility will also be explored.

## 3. Materials and Methods

### 3.1. General Information

All reagents and solvents were of commercial quality, purchased from Sigma-Aldrich (Madrid, Spain) and were used as received. Reactions were monitored by thin layer chromatography on aluminium plates coated with silica gel and fluorescent indicator. Separation by flash chromatography were performed on SDS 60 ACC, 230–400 mesh or Scharlau Ge 048 silica gel. Melting points were measured with Reichert 723 hot stage microscope (Vienna, Austria), or in open capillary tubes using a Büchi immersion instrument, and are uncorrected. Infrared spectra were recorded on a Perkin–Elmer Paragon 1000 spectrophotometer (Tres Cantos, Spain), with solid compounds compressed into KBr pellets and liquid compounds placed between two NaCl disks. NMR spectra data were obtained using Bruker spectrometers (Rivas-Vaciamadrid, Spain) maintained by the CAI de Resonancia Magnética, UCM, operating at 250 and 300 MHz for ^1^H NMR and, 63 and 75 MHz for ^13^C NMR; chemical shifts (δ) are given in parts per million and coupling constants (*J*) in Hertz. Elemental analyses were determined by the CAI de Microanálisis, Universidad Complutense, using a Leco CHNS-932 combustion microanalyzer (Tres Cantos, Spain). The enantiomeric excess of compounds **6ai** and **6aj** was assessed by ^1^H-NMR in the presence of 1.5 equivalents of (+) tris(3-heptafluoropropylhydroxymethylene) europium camphorate.

### 3.2. Preparation of Enantiomerically Pure 1-Azadienes—General Procedure

To a solution of crotonaldehyde in dry ethyl ether (5 mL) is added a catalytic amount of acetic acid (0.02 mL) and 1.00*–*3.10 mmol of suitable chiral auxiliary. The resulting solution is heated at reflux in a bath at 45 °C in the absence of moisture for 30 min. After checking by TLC that the reaction is complete, it is allowed to cool at room temperature and washed with saturated aqueous sodium bicarbonate solution (1 mL × 2). The organic phase is dried with anhydrous sodium sulfate and evaporated, obtaining the desirable products.

#### 3.2.1. (*S*)-*N*-(-But-2-en-1-ylidene)-2-(methoxymethyl)pyrrolidin-1-amine (**12a**)

This is obtained from 67 mg (0.96 mmol) of crotonaldehyde and 250 mg (1.90 mmol) of (*S*)-1-amino-methoxymethylpyrrolidine (SAMP), using the general procedure. Yield, 165 mg (95%) of compound **12a**.

^1^H NMR (CDCl_3_, 250 MHz) δ: 6.97 (d, 1H, *J* = 8.9 Hz, H-1′); 6.17 (ddd, 1H, *J* = 15.4, 8.9 and 1.6 Hz, H-2′); 5.74 (dq, 1H, *J* = 15.4 and 6.8 Hz, H-3′); 3.55 (m, 2H, CH_2_-OCH_3_); 3.42 (m, 1H, H-2); 3.36 (s, 3 H, OCH_3_); 2.83 (m, 2H, H-5); 1.95 (m, 4H, H-3.4); 1.77 (dd, 3H, *J* = 6.7 and 1.5 Hz, H-4′ ppm. ^13^C NMR (CDCl_3_, 63 MHz) δ: 138.76 (C-1′); 130.46 (C-2′); 129.38 (C-3′); 74.63 (CH_2_-O-CH_3_), 63.12 (C-2); 59.21 (OCH_3_): 49.55 (C-5); 26.67 (C-4); 22.22 (C-3); 18.22 (C-4′) ppm. These data were identical to those found in the literature [54].

#### 3.2.2. (*S*)-*N*-(But-2-en-1-ylidene)-2-(methoxydiphenylmethyl)pyrrolidin-1-amine (**12b**)

This is obtained from 124 mg (1.70 mmol) of crotonaldehyde and 500 mg (1.70 mmol) of (2*S*)-2-(1,1-diphenyl-1-methoxy)methylpirrolydine [47], using the general procedure. Yield, 534 mg (90%) of compound **12b**.

[Found: C, 79.34; H, 7.78; N, 8.25. C_22_H_26_N_2_O_,_ requires C, 79.00; H, 7.84; N, 8.38]. ^1^H NMR (CDCl_3_, 250 MHz) *δ*: 7.40*–*7.20 (m, 10H, 2 C_6_H_5_), 6.86 (d, 1H, *J* = 8.9 Hz, H-1′), 6.11 (ddd, 1 H, *J* = 15.4, 8.9 and 1.6 Hz, H-2′), 5.67 (dq, 1H, *J* = 15.4 and 6.6 Hz, H-3′), 3.18 (t, 1H, *J* = 6.5 Hz, H-2), 3.03 (s, 3H, OCH_3_), 2.84 (m, 2H, H-5), 2.0*–*1.82 (m, 4H, H-3,4), 1.75 (dd, 3H, *J* = 6.7 and 1.6 Hz, H-4′) ppm. ^13^C NMR (CDCl_3_, 63 MHz) δ: 142.02 (C-1″), 141.22 (C-1′), 134.35 (C-2′), 131.09 (C-3′), 130.15 and 129.75 (C-3″, 5″), 128.58 and 125.93 (C-4”), 127.12 and 126.99 (C-2″, 6″), 85.96 (C-OCH_3_), 67.50 (C-2), 51.45 (OCH_3_), 50.57 (C-5), 26.34 (C-4), 23.31 (C-3), 18.28 (C-4′) ppm.

#### 3.2.3. (*S*)-*N*-(2-(2-Methoxypropan-2-yl)pyrrolidin-1-yl)but-2-en-1-imine (**12c**)

Obtained from 221 mg of crotonaldehyde (3.10 mmol) and 500 mg (3.10 mmol) of (*S*)-2-(2-methoxypropan-2-yl)pyrrolidin-1-amine [47], using the general procedure. Yield, 541 mg (82%) of compound **12c**.

[Found: C, 68.82; H, 10.66; N, 13.25. C_12_H_22_N_2_O, requires C, 68.53; H, 10.54; N, 13.32]. ^1^H NMR (CDCl_3_, 250 MHz) δ: 6.95 (d, 1H, *J* = 8.9 Hz, H-1′), 6.15 (ddd, 1H, *J* = 15.4, 8.9 and 1.6 Hz, H-2′), 5.70 (dq, 1H, *J* = 15.4 and 6.7 Hz, H-3′), 3.65 (m, 1H, H-2), 3.33 (s, 3H, OCH_3_), 2.85 (m, 2H, H-5), 1.90 (m, 4H, H-3,4), 1.75 (dd, 3H, *J* = 6.7 and 1.6 Hz, H-4′), 1.15–1.05 (2 s, 6H, 2CH_3_). ^13^C NMR (CDCl_3_, 63 MHz) δ: 136.18 (C-1′), 130.86 (C-2′), 129.80 (C-3′), 72.03 (C-O-CH_3_), 65.02 (C-2), 51.34 (OCH_3_), 49.68 (C-5), 24.93 (C-4), 23.93 (C-3), 22.97 and 21.07 (2 CH_3_); 18.5 (C-4′).

#### 3.2.4. (*R*)-*N*-(2-(2-Methoxypropan-2-yl)pyrrolidin-1-yl)but-2-en-1-imine (**12d**)

Obtained from 79 mg (1.10 mmol) of crotonaldehyde and 180 mg (3.80 mmol) of (*R*)-2-(2-methoxypropan-2-yl)pyrrolidine-1-amine [47], using the general procedure. Yield, 140 mg (58%) of compound **12d**, whose spectroscopic data are identical to those found for its enantiomer **12c**. [Found: C, 68.88; H, 10.47; N, 13.46. C_12_H_22_N_2_O, requires C, 68.53; H, 10.54; N, 13.32].

### 3.3. Hetero Diels–Alder Reactions between Dimethylhydrazones ***9*** and Quinones ***8***—General Procedure

Method A: To a solution of quinones **8** (0.05 to 0.55 mmol) in chloroform or dry THF (5–20 mL) was added the suitable azadiene **9** (1.05 to 2 equiv.). The solution was stirred at room temperature (5 min–1 h), and the solvent was evaporated. The residue was chromatographed on silica gel eluting with a gradient from neat dichloromethane or ethyl ether to neat ethyl acetate to yield compounds **5** or **6** in the yields shown in Table 2.

Method B: To silica gel-supported suitable quinone **8** (0.06–0.55 mmol), the suitable dimethylhydrazone **9** was rapidly added (2 eq). The column was then eluted with a gradient from dichloromethane or ethyl ether to ethyl acetate, affording compounds **5** or **6** in the yields shown in Table 2.

Method C: A suspension of carboxy polystyrene resin, prepared from 2% cross-linked Merrifield resin (2–2.5 meq Cl/g, 200–400 mesh) using a literature method [55] (5.0 g) in dry toluene (20 mL) was stirred at room temperature under an argon atmosphere for 30 min. Thionyl chloride (20 mL) was added, and the suspension was refluxed for 24 h with vigorous stirring and then cooled. The chloroformyl polystyrene resin thus obtained was filtered and washed with toluene (2 × 25 mL) and ethyl ether (2 × 25 mL). If stored at −18 °C under argon, it could be used for 2–3 months without a significant loss of activity.

To a stirred suspension of the suitable 2,5,8(1*H*)-quinolinetrione **8** (0.25 mmol) and the chloroformyl polystyrene resin (1.25 g, 5 eq) in chloroform (30 mL) was added the suitable dimethylhydrazone **9** (0.60 mmol). The suspension was stirred at room temperature for 5 min and filtered. The filtrate was evaporated and then residue was chromatographed on silica gel, affording compounds **5** or **6** in the yields shown in Table 2.

Characterization data for new compounds follow. Exchangeable spectral assignments are marked with asterisks.

#### 3.3.1. 6-Butyl-4-methyl-1*H*-1,8-diazaanthracene-2,9,10-trione (**5f**)

[Found: C, 68.58; H, 5.23; N, 9.21. C_17_H_16_N_2_O_3_ requires C, 68.91; H, 5.44; N, 9.45]; Mp, 190–193 °C. ν*_max_* (KBr): 3431 (NH), 1673 and 1645 (C=O) cm^−1^. ^1^H NMR (CDCl_3_, 250 MHz) δ: 9.79 (br s, 1H, NH), 8.84 (s, 1H, C_7_-H), 8.37 (s, 1H, C_5_-H), 6.69 (s, 1H, C_3_-H), 2.81 (t, 2H, *J* = 7.7 Hz, C_6_-CH_2_), 2,68 (s, 3H, C_4_-CH_3_), 1.71–1.62 (m, 2H, C_6_-CH_2_-CH**_2_**), 1.43–1.34 (m, 2H, C_6_-(CH_2_)_2_-CH**_2_**), 0.94 (t, 3H, *J* = 7.1 Hz, C_6_-(CH_2_)_3_-CH**_3_**) ppm. ^13^C NMR (CDCl_3_, 63 MHz) δ: 181.04 (C-9), 176.51 (C-10), 160.28 (C-2), 155.23 (C-7), 151,86 (C-4), 145.43 (C-8a), 144.08 (C-6), 140.33 (C-9a), 134.50 (C-5), 130.52 (C-10a), 128.13 (C-3), 115.59 (C-4a), 33.26* (C_6_-CH_2_), 32.77* (C_6_-CH_2_-CH**_2_**), 22.79 (C_4_-CH_3_), 22.34 (C_6_-(CH_2_)_2_-CH**_2_**), 13.89 (C_6_-(CH_2_)_3_-CH**_3_**) ppm.

#### 3.3.2. 5,8-Dihydro-4,6-dimethyl-5-propyl-1*H*-1,8-diazaanthracene-2,9,10-trione (**6k**)

Method A. From 81 mg of **9i** (0.50 mmol) and 100 mg (0.50 mmol) of **8b**. Reaction time 10 min, affording 51 mg (32%) of **6k** and 49 mg (40%) of the corresponding quinone **10**. [Found: C, 68.09; H, 6.16; N, 9.15. C_17_H_18_N_2_O_3,_ requires C, 68.44; H, 6.08; N, 9.39]. Mp, 235–238 °C. ν*_max_* (KBr) 3420 (NH), 1654 (C=O) cm^−1^. ^1^H NMR (CDCl_3_, 250 MHz) δ: 6.85 (d, 1H, *J* = 1.2 Hz, C_3_-H), 6.70 (br s, 1H, N_8_-H), 6.11 (d, 1H, *J* = 1.2 Hz, C_7_-H), 3.57 (t, 1H, *J* = 4.7 Hz, C_5_-H), 2.56 (d, 3H, *J* = 1.2, C_4_-CH_3_), 1.71 (d, 3H, *J* = 1.2 Hz, C_6_-CH_3_), 1.46 (m, 2H, C5-CH_2_), 1.17 (m, 2H, C_5_-CH_2_-CH_2_-), 0.8 (t, 3H, *J* = 7.9, C5-CH_2_-CH_2_-CH_3_) ppm. ^13^C NMR (CDCl_3_, 63 MHz) δ: 183.24 (C-9), 176.06 (C-10), 161.67 (C-2), 152.34 (C-4), 137.11 (C-9a); 136.70 (C-8a); 127.70 (C-3), 119.47 (C-7), 116.13 (C6), 115.24 (C-4a), 112.04 (C-10a), 35.85 (C_5_-CH_2_-CH_2_-CH_3_), 35.55 (C-5), 22.75 (C_4_-CH_3_), 18.95 (C_5_-CH_2_-CH_2_-CH_3_), 18.69 (C_6_-CH_3_), 14.43 (C_5_-CH_2_-CH_2_-CH_3_) ppm.

#### 3.3.3. 3,4,6-Trimethyl-1*H*-1,8-diazaanthracene-2,9,10-trione (**5l**)

Method A. From 15 mg (0.07 mmol) of **8d** and 10 mg (0.09 mmol) of **9b**, affording 16 mg (76%) of **5l** and 2 mg (11%) of the corresponding quinone **10**. [Found: C, 66.86; H, 4.25; N, 10.45. C_15_H_12_N_2_O_3_ requires C, 67.16; H, 4.51; N, 10.44]; Mp, >300 °C. ν*_max_* (KBr): 3428 (NH), 1628 (C=O) cm^−1^. ^1^H NMR (CDCl_3_, 250 MHz) δ: 9.71 (br s, 1H, NH), 8.84 (d, 1H, *J* = 1.6 Hz C_7_-H), 8.30 (d, 1H, *J* = 1.6 Hz, C_5_-H), 2.67 (d, 3H, *J* = 0.6 Hz, C_4_-CH_3_), 2.55 (s, 3H, C_6_-CH_3_), 2.26 (d, 3H, *J* = 0.6 Hz, C_3_-CH_3_) ppm. ^13^C NMR (CDCl_3_, 63 MHz) δ: 182.95 (C-9), 178.31 (C-10), 162.92 (C-2), 155.69 (C-7), 148,31 (C-4), 146.07 (C-8a), 145.58 (C-6), 141.33 (C-9a), 140.42 (C-3), 128.23 (C-10a), 116.65 (C-4a), 19.88 (C_6_-CH_3_), 19.26 (C_4_-CH_3_), 13.74 (C_3_-CH_3_) ppm. C-5 was not observed.

#### 3.3.4. 3-Benzyl-4,6-dimethyl-1*H*-1,8-diazaanthracene-2,9,10-trione (**5m**)

Method B. From 18 mg (0.07 mmol) of **8e** and 15 mg (0.12 mmol) of **9b** to get 15 mg (68%) of **5m**. [Found: C, 73.09; H, 4.48; N, 7.95. C_21_H_16_N_2_O_3_ requires C, 73.24; H, 4.68; N, 8.13]. Mp, 260–262 °C. ν*_max_* (KBr): 3421 (NH), 1641 (C=O) cm^−1^. ^1^H NMR (CDCl_3_, 250 MHz) δ: 9.72 (br s, 1H, NH), 8.84 (s, 1H, C_7_-H), 8.29 (s, 1H, C_5_-H), 7.24 (br s, 5H, Ph-H), 4.14 (s, 2H, C_3_-CH_2_), 2.70 (s, 3H, C_4_-CH_3_), 2.55 (s, 3H, C_6_-CH_3_) ppm. ^13^C NMR (CDCl_3_, 63 MHz) δ: 181.26 (C-9), 176.33 (C-10), 160.59 (C-2), 155.28 (C-7), 147.63 (C-4), 143.63 (C-8a), 140.46 (C-6), 138.76 (C-1′), 138.33 (C-9a), 138.18 (C-3*), 135.22 (C-5), 130.89 (C-10a), 128.59 (C-2′, C-6′), 128.34 (C-3′, C-5′), 126.41 (C-4′) 116.05 (C-4a), 32.23 (C_3_-CH_2_), 19.21 (C_6_-CH_3_), 18.45 (C_4_-CH_3_) ppm.

#### 3.3.5. 4-Ethyl-6-methyl-1*H*-1,8-diazaanthracene-2,9,10-trione (**5n**)

Method A. From 50 mg (0.25 mmol) of **8f** and 33 mg (0.30 mmol) of **9b** to obtain 46 mg (70%) of **5m**, 6 mg (9%) of the corresponding quinone **10** and 5 mg (11%) of **5ah**. [Found: C, 66.86; H, 4.29; N, 10.03. C_15_H_12_N_2_O_3_ requires: C, 67.16; H, 4.51; N, 10.44]. Mp, 301–303 °C. ν*_max_* (KBr): 3460 (NH), 1640 (C=O) cm^−1^. ^1^HNMR (CDCl_3_, 250 MHz) δ: 9.71 (br s, 1H, NH), 8.87 (d, 1H, *J* = 1.7 Hz, C_7_-H), 8.33 (d, 1H, *J* = 1.7 Hz, C_5_-H), 6.75 (s, 1H, C_3_-H), 3.15 (q, 2H, *J* = 7.3 Hz, C_4_-CH_2_-CH_3_), 2.58 (s, 3H, C_6_-CH_3_), 1.28 (t, 3H, *J* = 7.3 Hz, C_4_-CH_2_-CH_3_) ppm. ^13^C NMR (d_5_-pyridine, 63 MHz) δ: 181.31 (C-9), 177.39 (C-10), 162.15 (C-2), 156.31 (C-4), 154.94 (C-7), 144.81 (C-8a), 143.75 (C-6), 139.66 (C-9a), 135.60 (C-5), 130.35 (C-10a), 125.64 (C-3), 114.71 (C-4a), 26.53 (CH_2_- CH_3_), 16.89 (C_6_-CH_3_), 12.40 (CH_2_-CH_3_) ppm.

#### 3.3.6. 4-Acetyl-6-methyl-1*H*-1,8-diazaanthracene-2,9,10-trione (**5ah**)

Isolated as a side product of the preparation of **5n**. [Found: C, 63.94; H, 3.46; N, 9.42. C_15_H_10_N_2_O_4_ requires: C, 63.83; H, 3.57; N, 9.93]. Mp >300 °C. ν*_max_* (KBr): 3570 (NH), 1715 (CO-CH_3_), 1650 (C=O, *p*-quinone) cm^−1^. ^1^H NMR (CDCl_3_, 250 MHz) δ: 9.83 (br s, 1H, NH), 8.92 (br s, 1H, C_7_-H), 8.33 (br s, 1H, C_5_-H), 6.66 (s, 1H, C_3_-H), 2,59 (s, 3H, C_6_-CH_3_), 2.57 (s, 3H, C_4_-CO-CH_3_) ppm. ^13^C NMR (d_5_-pyridine, 63 MHz) δ: 200.58 (CO-CH_3_), 180.03 (C-9), 176.56 (C-10), 162.49 (C-2), 155.33 (C-7), 152.16 (C-4), 145.40 (C-8a), 143.47 (C-6), 139.70 (C-9a), 134.90 (C-5), 129.05 (C-10a), 122.31 (C-3), 113.47 (C-4a), 30.06 (CO-CH_3_), 18.21 (C_6_-CH_3_) ppm.

#### 3.3.7. 6-Methyl-4-(2-phenylethyl)-1*H*-1,8-diazaanthracene-2,9,10-trione (**5o**)

Method A: from 20 mg (0.07 mmol) of **8g** and 9 mg (0.08 mmol) of **9b** to get 49% of **5o** and 15% of the corresponding quinone **10**. Method B: From 40 mg (0.14 mmol) of **8g** and 32 mg (0.29 mmol) of **9b** to yield 82% of **5o**. [Found: C, 72.82; H, 4.73; N, 8.22. C_21_H_16_N_2_O_3_ requires: C, 73.24; H, 4.68; N, 8.13]. Mp, 262–264 °C. ν*_max_* (KBr): 3416 (NH), 1662 (C=O) cm^−1^. ^1^HNMR (CDCl_3_, 250 MHz) δ: 9.75 (br s, 1H, NH), 8.90 (s, 1H, C_7_-H), 8.39 (s, 1H, C_5_-H), 7.32 (br s, 5H, Ph-H), 6.67 (s, 1H, C_3_-H), 3.40 (t deform, 2H, *J* = 7.6 and 8.1 Hz, C_4_-CH_2_), 2.92 (dd, 2H, *J* = 7.2 and 8.6 Hz, Ph-CH_2_), 2.60 (s, 3H, C_6_-CH_3_) ppm. ^13^C NMR (d_6_-DMSO, 63 MHz) δ: 181.29 (C-9), 176.59 (C-10), 160.94 (C-2), 154.65 (C-4), 153.67 (C-7), 144.23 (C-8a), 142.98 (C-6), 141.10 (C-1′), 139.26 (C-9a), 134.58 (C-5), 130.21 (C-10a), 128.48 (C2′, C6′), 128.29 (C3′, C5′), 128.20 (C-4′), 126.00 (C-3), 114.48 (C-4a), 36.45 (C_4_-CH_2_), 35.56 (Ph-CH_2_), 18.51 (C_6_-CH_3_) ppm.

#### 3.3.8. 6-Methyl-1-(4-tolyl)-1,8-diazaanthracene-2,9,10-trione (**5p**)

After carrying out the Diels Alder reaction on 19 mg (0.07 mmol) of **8h** and 16 mg (0.13 mmol) of **9b** according to method B, the reaction crude was dissolved in 10 mL of dichloromethane and 62 mg (0.72 mmol) of 85% activated manganese oxide was added. The solution was stirred at room temperature for 24 h and filtered through a layer of celite which was washed twice with 30 mL chloroform. The combined organic phases were dried over anhydrous sodium sulphate, evaporated and purified by silica gel column chromatography eluting with a gradient from dichloromethane to ethyl acetate, yielding 12 mg (52%) of **5p**, 6 mg (26%) of **11** and 2 mg (9%) of the corresponding quinone **10**.

[Found: C, 72.58; H, 3.96; N, 8.12. C_20_H_14_N_2_O_3_ requires: C, 72.72; H, 4.27; N, 8.48]. Mp, >300 °C. ν*_max_* (KBr): 1684 (CO, p-quinone), 1665 (HN-CO) cm^−1^. ^1^H NMR (CDCl_3_, 250 MHz) δ: 8.86 (d, 1H, *J* = 1.8 Hz, C7-H), 8.37 (d, 1H, J = 9.7 Hz, C_4_-H), 8.04 (d, 1H, *J* = 1.7 Hz, C_5_-H), 7.33 (d, 2H, *J* = 8.1 Hz, C_3′,5′_-H), 7.05 (d, 2H, *J* = 8.3 Hz, C_2′,6′_-H), 7.01 (d, 1H, *J* = 9.7 Hz, C3-H), 2.46 (s, 6H, C_4_′-CH_3_ and C_6_-CH_3_) ppm. 13C NMR (CDCl_3_, 63 MHz) δ: 179.06 (C-9), 177.88 (C-10), 162.30 (C-2), 155.91 (C-7), 144.34 (C-8a), 140.58 (C-6), 138.90* (C-9a), 138.74* (C-1′), 135.86 (C-4′), 135.72* (C-5), 135.00* (C-4), 130.10 (C-3′, C5′), 129.23 (C-10a), 127.03 (C-3), 126.64 (C-2′, C-6′), 119.33 (C-4a), 21.34 (C_4′_-CH_3_), 18.78 (C_6_-CH_3_) ppm.

#### 3.3.9. 7-Methyl-1-(4-tolyl)-1,5-diazaanthracene-2,9,10-trione (**11**)

[Found: C, 72.63; H, 4.02; N, 8.57. C_20_H_14_N_2_O_3_ requires C, 72.72; H, 4.27; N, 8.48]. Mp, 300–302 °C. ν*_max_* (KBr): 1694 (CO, p-quinone), 1657 (HN-CO) cm^−1^. ^1^H NMR (CDCl_3_, 250 MHz) δ: 8.76 (br s, 1H, C_6_-H), 8.27 (br s, 1H, C_8_-H), 8.26 (d, 1H, *J* = 9.6 Hz, C_4_-H), 7.29 (d, 2H, *J* = 8.1 Hz, C_3′,5′_-H), 7.04 (d, 2H, *J* = 8.6 Hz, C_2′,6′_-H), 7.00 (d, 1H, *J* = 9.8 Hz, C_3_-H), 2.51 (s, 3H, C_7_-CH_3_), 2.41 (s, 3H, C_4′_-CH_3_) ppm. 13C NMR (CDCl_3_, 63 MHz) δ: 180.17 (C-9), 176.31 (C-10), 162.38 (C-2), 155.60 (C-6), 145.80 (C-10a), 141.90 (C-7), 139.29 (C-9a), 138.67 (C-1′), 135.60 (C-4′), 135.16 (C-8), 134.04 (C-4), 130.09 (C-3′, C5′), 127.48 (C-8a), 126.82 (C-3), 126.55 (C-2′, C6′), 118.30 (C-4a), 21.42 (C_4′_-CH_3_), 19.07 (C_7_-CH_3_) ppm.

#### 3.3.10. 6-Methyl-3-(*p*-tolyl)-1,8-diazaanthracene-2,9,10-trione (**5q**)

Method B: using 14 mg (0.05 mmol) of **8i** and 12 mg (0.10 mmol) of **9b** to afford 16 mg (94% of **5q**. [Found: C, 72.63; H, 3.90; N, 8.69. C_20_H_14_N_2_O_3_ requires C, 72.72; H, 4.27; N, 8.48]. Mp, 298–300 °C. ν*_max_* (KBr): 3421 (NH), 1684 (CO, quinone), 1654 (HN-CO) cm^−1^; ^1^H NMR (CDCl_3_, 250 MHz) δ: 9.70 (s, 1H, NH), 8.88 (d, 1H, *J* = 1.7 Hz, C_7_-H), 8.35 (d, 1H, *J* = 1.7 Hz, C_5_-H), 8.23 (s, 1H, C_4_-H), 7.27 (d, 2H, *J* = 8.3 Hz, C_2′,6′_-H), 7.22 (d, 2H, *J* = 8.2 Hz, C_3′,5′_-H), 2.57 (s, 3H, C_4′_-CH_3_), 2.39 (s, 3H, C_6_-CH_3_) ppm. ^13^C NMR (CDCl_3_, 63 MHz) δ: 179.75 (C-9), 175.84 (C-10), 160.32 (C-2), 155.43 (C-7), 144.94 (C-8a), 140.18 (C-6), 139.99 (C-9a), 139.28 (C-3), 137.41 (C-4′), 134.95 (C-5), 131.77 (C-4), 131.48 (C-1′), 129.48 (C-10a), 129.25 (C-3′, C5′), 128.58 (C-2′, C6′), 116.52 (C-4a), 21.39 (C_4′_-CH_3_), 19.16 (C_6_-CH_3_) ppm.

#### 3.3.11. 5,8-Dihydro-3,4,5-trimethyl-1*H*-1,8-diazaanthracene-2,9,10-trione (**6r**)

Method A: from 72 mg (0.36 mmol of **8d** and 44 mg (0.39 mmol) of **9e** to afford 30 mg (32%) of **6r** and 37 mg (42%) of the corresponding quinone **10**. Method B: from 45 mg (0.22 mmol of **8d** and 50 mg (0.44 mmol of **9e** to afford 42 mg (70%) of **6r** and 3 mg (6%) of the corresponding quinone **10**. [Found: C, 62.23; H, 5.18; N, 10.27. C15H14N2O3 requires C, 66.66; H, 5.22; N, 10.36]. Mp > 300 °C. ν*_max_* (KBr): 3426, 3285 (NH), 1648, 1630 (C=O) cm^−1^; ^1^H NMR (d_5_-pyridine, 250 MHz) δ: 9.99 (br s, 1H, NH), 6.63 (dd, 1H, *J* = 7.6 and 4.5 Hz, C_7_-H), 5.14 (t, 1H, *J* = 6.1 Hz, C_6_-H), 4.09 (m, 1H, C_5_-H), 2.80 (s, 3H, C_4_-CH_3_), 2.47 (s, 3H, C_3_-CH_3_), 1.49 (d, 3H, *J* = 6.5 Hz, C_5_-CH_3_) ppm. 13C NMR (d5-pyridine, 63 MHz) δ: 184.28 (C-9), 177.11 (C-10), 161.90 (C-2), 145.39 (C-4), 138.18 (C-8a), 134.47 (C-9a), 124.07 (C-7), 115.04 (C-4a), 114.26 (C-10a), 107.88 (C-6), 26.22 (C-5), 24.63 (C_5_-CH_3_), 17.59 (C_4_-CH_3_), 12.92 (C_3_-CH_3_) ppm. The C_3_ signal was overlapped with others.

#### 3.3.12. 5,8-Dihydro-4-ethyl-5-methyl-1*H*-1,8-diazaanthracene-2,9,10-trione (**6s**)

Method A: from 90 mg (0.44 mmol of **8f** and 55 mg (0.49 mmol) of **9e** to afford 34 mg (29%) of **6s** and 64 mg (59%) of the corresponding quinone **10**. Method B: from 60 mg (0.29 mmol of **8f** and 66 mg (0.59 mmol of **9e** to afford 66 mg (84%) of **6s**. [Found: C, 66.37; H, 5.24; N, 9.97. C_15_H_14_N_2_O_3_ requires C, 66.65; H, 5.22; N, 10.36]. Mp, > 295 °C. ν*_max_* (KBr): 3580 (NH), 1655, 1645 (C=O) cm^−1^; ^1^H NMR (d_5_-pyridine, 250 MHz) δ: 10.12 (br s, 1H, NH), 6.98 (s, 1H, C_3_-H), 6.65 (dd, 1H, *J* = 7.6 and 4.5 Hz, C_7_-H), 5.13 (dd, 1H, *J* = 7.5 and 4.8 Hz, C_6_-H), 4.09 (qd, 1H, *J* = 6.5 and 6.4 Hz C_5_-H), 3.29 (m, 2H, C_4_-CH_2_-CH_3_), 1.46 (d, 3H, *J* = 6.5 Hz, C_4_-CH_2_-CH_3_), 1.34 (t, 3H, *J* = 7.3 Hz, C_4_-CH_2_-CH_3_ ppm. ^13^C NMR (d_5_-pyridine, 63 MHz) δ: 183.65 (C-9), 177.32 (C-10), 162.43 (C-2), 156.42 (C-4), 138.22 (C-8a), 135.74 (C-9a), 125.38 (C-3), 124.24 (C-7), 114.43 (C-4a), 114.35 (C-10a), 108.00 (C-6), 27.95 (CH_2_-CH_3_), 26.98 (C-5), 24.73 (C_5_-CH_3_), 14.30 (CH_2_-CH_3_) ppm.

#### 3.3.13. 5,8-Dihydro-5-methyl-4-propyl-1*H*-1,8-diazaanthracene-2,9,10-trione (**6t**)

Method A: from 120 mg (0.55 mmol of **8j** and 65 mg (0.58 mmol) of **9e** to afford 50 mg (32%) of **6t** and 72 mg (50%) of the corresponding quinone **10**. Method B: from 25 mg (0.09) mmol of **8j** and 20 mg (0.18 mmol of **9e** to afford 30 mg (92%) of **6t**. [Found: C, 67.80; H, 5.56; N, 10.06. C_16_H_16_N_2_O_3_ requires C, 67.59; H, 5.67; N, 9.85]. Mp, 234–236 °C. ν*_max_* (KBr): 3401 (NH), 1660, 1625 (C=O) cm^−1^; ^1^H NMR (d_5_-pyridine, 250 MHz) δ: 10.10 (br s, 1H, NH), 6.98 (s, 1H, C_3_-H), 6.64 (dd, 1H, *J* = 7.6 and 4.5 Hz, C_7_-H), 5.13 (m, 1H, C_6_-H), 4.08 (qd, 1H, *J* = 6.5 and 6.4 Hz C_5_-H), 3.24 (m, 2H, C_4_-CH_2_-CH_3_), 1.79 (sext, 2 H, *J* = 7.4 Hz, C_4_-CH_2_-CH_3_), 1.47 (d, 3H, *J* = 6.6 Hz, C_5_-CH_3_), 1.15 (t, 3H, *J* = 7.3 Hz, C_4_-CH_2_-CH_3_) ppm. ^13^C NMR (d_5_-pyridine, 63 MHz) δ: 183.48 (C-9), 179.45 (C-10), 162.15 (C-2), 154.70 (C-4), 139.93 (C-8a), 138.07 (C-9a), 126.13 (C-3), 124.07 (C-7), 114.26 (C-4a and C-10a), 107.00 (C-6), 36.60 (C4-CH_2_), 26.13 (C-5), 24.56 (C_5_-CH_3_), 23.28 (C4-CH_2_-CH_2_), 14.30 (C4-CH_2_-CH_3_) ppm.

#### 3.3.14. 5,8-Dihydro-5-methyl-1-(4-tolyl)-1,8-diazaanthracene-2,9,10-trione (**6v**)

Method B: from 19 mg (0.07 mmol) of **8h** and 16 mg (0.14 mmol) of **9e** to yield 19 mg (80%) of **6v**. [Found: C, 72.21; H, 4.96; N, 8.22. C_20_H_16_N_2_O_3_ requires C, 72.28; H, 4.85; N, 8.43]. Mp, 136–138 °C. ν*_max_* (KBr): 1641 (C=O) cm^−1^; ^1^H NMR (CDCl_3_, 250 MHz) δ: 8.01 (d, 1H, *J* = 9.6 Hz, C_4_-H), 7.28 (d, 2H, *J* = 7.9 Hz, C_2′,6′_-H), 6.97 (d, 2H, *J* = 7.3 Hz, C_3′,5′_-H), 6.79 (d, 2H, *J* = 9.6 Hz, C_3_-H y NH), 6.10 (dd, 1H, *J* = 7.7 and 4.4 Hz, C_7_-H), 4.92 (m, 1H, C_6_-H), 3.48–3.43 (m, 1H, C_5_-H), 2.42 (s, 3H, C_4′_-CH_3_), 0.98 (d, 3H, *J* = 6.6 Hz, C_5_-CH_3_) ppm. ^13^C NMR (CDCl_3_, 63 MHz) δ: 178.26 (C-9), 177.12 (C-10), 162.96 (C-2), 138.13 (C-1′), 137.58 (C-9a), 136.40 (C-8a), 134.50 (C-4′), 129.91 (C-3′, C-5′), 126.51 (C-4), 126.20 (C-2′, 6′), 123.48 (C-3), 122.02 (C-7), 114.07 (C-4a), 113.91 (C-10a), 110.72 (C-6), 26.06 (C-5), 23.88 (C_5_-CH_3_), 21.56 (C_4′_-CH_3_) ppm.

#### 3.3.15. 5-Methyl-3-(4-tolyl)-1*H*-1,8-diazaanthracene-2,9,10-trione (**5w**)

Method B: from 10 mg (0.04 mmol) of **8i** and 9 mg (0.10 mmol) of **9e** to yield 8 mg (64%) of **5w**. Found: C, 72.51; H, 4.37; N, 8.20. C_20_H_14_N_2_O_3_ requires C, 72.72; H, 4.27; N, 8.48]. ^1^HNMR (CDCl_3_, 250 MHz) δ: 9.62 (br s, 1H, NH), 8.85 (d, 1H, *J* = 4.9 Hz, C_7_-H), 8.22 (s, 1H, C_4_-H), 7.72 (d, 2H, *J* = 8.2 Hz, C_2′,6′_-H), 7.51 (d, 1H, *J* = 5.3 Hz, C_6_-H), 7.26 (d, 2H, *J* = 8.5 Hz, C_3′,5′_-H), 2.88 (s, 3H, C_5_-CH_3_), 2.39 (s, 3H, C_4′_-CH_3_) ppm.

#### 3.3.16. 5,8-Dihydro-5-ethyl-4-methyl-1,8-diazaanthracene-2,9,10-trione (**6ae**)

[Found: C, 66.30; H, 5.30; N, 10.14. C_15_H_14_N_2_O_3_ requires C, 66.66; H, 5.22; N, 10.36]. Mp, 215–218 °C. ν*_max_* (KBr) 3403 (NH), 1658 (C=O) cm^−1^. ^1^H NMR (*d*_6_-DMSO, 250 MHz) δ: 9.72 (br s, 1H, NH), 6.99–6.88 (m, 1H, C_3_-H), 6.54–6.34 (m, 1H, C_7_-H), 6.36–6.33 (m, 1H, C_6_-H), 4.73 (br. s, 1H, C_5_-H), 2.40 (s, 3H, C_4_-CH_3_), 1.4–1.2 (m, 2H, C_5_-CH_2_), 0.75 (t, 3H, *J* = 7.5 Hz, C_5_-CH_2_-CH_3_) ppm. ^13^C NMR (*d*_5_-pyridine, 63 MHz) δ: 183.97 (C-9), 177.21 (C-10), 162.24 (C-2), 150.86 (C-4), 139.67 (C-8a), 139.20 (C-9a), 127.08 (C-3), 125.33 (C-7), 112.81 (C-4a and C-10a), 106.52 (C-6), 32.36 (C-5), 30,68 (C_5_-CH_2_), 22.59 (C_4_-CH_3_), 9.57 (C_5_-CH_2_-CH_3_) ppm.

#### 3.3.17. 5,8-Dihydro-4-methyl-5-propyl-1,8-diazaanthracene-2,9,10-trione (**6af**)

[Found: C, 67.13; H, 5.36; N, 9.45. C_16_H_16_N_2_O_3_ requires C, 67.59; H, 5.67; N, 9.85]. Mp, 190–192 °C. ν*_max_* (KBr) 3394 (NH), 1652 (C=O) cm^−1^. ^1^H NMR (*d*_5_-pyridine, 250 MHz) δ: 10.11 (br s, 1H, NH), 6.91 (s, 1H, C_3_-H), 6.71–6.69 (m, 1H, C_7_-H), 5.14–5.13 (m, 1H, C_6_-H), 4.13–4.00 (m, 1H, C_5_-H), 3.02 (br s, 2H, C_5_-CH_2_-), 2.77 (s, 3H, C_4_-CH_3_), 2.8–1.8 (m, 2H, C_5_-CH_2_-CH_2_), 1.05 (t, 3H, *J* = 7.1 Hz, C_5_-(CH_2_)_2_-CH_3_) ppm. ^13^C NMR (*d*_5_-pyridine, 63 MHz) δ: 184.62 (C-9), 177.10 (C-10), 162.00 (C-2), 150.58 (C-4), 139.37 (C-8a), 138.70 (C-9a), 126.75 (C-3), 124.80 (C-7), 116.50 and 113.96 (C-4a and C-10a), 107.04 (C-6), 41.28 (C_5_-CH_2_), 31.60 (C-5), 23.26 (C_4_-CH_3_), 19.18 (C_5_-CH_2_-CH_2_-), 15.04 (C_5_-(CH_2_)_2_-CH_3_) ppm.

#### 3.3.18. 5,8-Dihydro-5-butyl-4-methyl-1*H*-1,8-diazaanthracene-2,9,10-trione (**6ag**)

[Found: C, 68.12; H, 5.79; N, 9.02. C_17_H_18_N_2_O_3_ requires C, 68.44; H, 6.08; N, 9.39]. Mp, 190–191 °C. ν*_max_* (KBr) 3390 (NH), 1657 (C=O) cm^−1^. ^1^H NMR (*d*_6_-DMSO, 250 MHz) δ: 11.80 (br s, 1H, NH), 6.52 (s, 1H, C_3_-H), 6.19–6.17 (m, 1H, C_7_-H), 4.9–4.8 (m, 1H, C_6_-H), 3.51–3.48 (m, 1H, C_5_-H), 2.74 (s, 3H, C_4_-CH_3_), 1.4–1.0 (m, 9H, C_5_-(CH_2_)_2_-CH_3_) ppm. ^13^C NMR (*d*_5_-pyridine, 63 MHz) δ: 183.66 (C-9), 177.00 (C-10), 161.92 (C-2), 150.56 (C-4), 139.42 (C-8a), 138.73 (C-9a), 127.82 (C-3), 126.75 (C-7), 116.50 and 113.02 (C-4a and C-10a), 106.10 (C-6), 37.80 (C_5_-CH_2_), 30.73 (C-5), 27.12 (C_5_-CH_2_-CH_2_-), 22.75 (C_5_-(CH_2_)_2_-CH_2_-), 22.28 (C_4_-CH_3_), 13.75 (C_5_-(CH_2_)_3_-CH_3_) ppm.

### 3.4. Diels–Alder Reaction with Chiral 1-Dialkylamino-1-Azadienes—General Procedure

A solution of quinone **8c** (50 mg, 0.02 mmol) and suitable azadiene **12c** or **12b** (1.1 eq.) in chloroform (5 mL) was refluxed for 3.5 h. The solution was evaporated, and the residue was chromatographed on silica gel eluting with a gradient from dichloromethane to dichloromethane-ethyl acetate mixture (6:4) yielding products **6ai** (40%, 85% ee) and **6aj** (30%, 83% ee).

#### 3.4.1. (5*R*)-1,4,5-Trimethyl-5,8 Dihydro-1*H*-1,8-diazaanthracene-2,9,10-trione (**6ai**)

[Found: C, 66.83; H, 5.43; N, 9.96. C_15_H_14_N_2_O_3_ requires: C, 66.66; H, 5.22; N, 10.36]. Mp, 165–167 °C. ν*_max_* (KBr): 3247.7 (NH), 1664.1 (C=O) cm^−1^. ^1^H NMR (CDCl_3_, 250 MHz) δ: 6.67 (s, 1H, C_3_-H), 6.49 (br s, 1H, C_8_-H), 6.13 (dd, 1H, *J* = 7.7 and 4.4 Hz, C_7_-H), 4.89 (t, 1H, *J* = 6.7 Hz, C_6_-H), 3.84 (s, 3H, N_1_- CH_3_), 3.64 (m, 1H, C_5_-H), 2.57 (s, 3H, C_4_-CH_3_), 1.12 (d, 3H, *J* = 6.6 Hz, C_5_-CH_3_) ppm. ^13^C NMR (CDCl_3_, 63 MHz) δ: 183.39 (C-9), 177.92 (C-10), 161.97 (C-2), 149.56 (C-4), 139.14 (C-8a), 137.48 (C-9a), 127.02 (C-3), 122.86 (C-7), 118.41 (C-4a), 114.22 (C-10a), 108.88 (C-6), 34.20 (N_1_-CH_3_), 26.29 (C-5), 25.78 (C_4_-CH_3_), 24.39 (C_5_-CH_3_) ppm.

#### 3.4.2. (5*S*)-1,4,5-Trimethyl-5,8 Dihydro-1*H*-1,8-diazaanthracene-2,9,10-trione (**6aj**)

The spectroscopic data are identical to those of the compound **6ai**.

### 3.5. Dehydrogenation of Dihydro Derivatives ***6*** to 1,8-Diazaanthracene-2,9,10-triones ***5***—General Procedures

**Method A:** To a solution of the suitable dihydro derivative **6** (0.01–0.20 mmol) in dichloromethane (1–10 mL) was added activated 85% MnO_2_ (5 eq–10 eq), the solution was stirred (10 min-24 h) at r.t. and then it was filtered through celite pad and washed twice with 30 mL of dichloromethane. The solvent was evaporated and, when necessary, chromatographed on silica gel, eluting with ethyl acetate, to afford compounds **6**.

**Method B:** A solution of the suitable dihydro derivate **6** (0.06 to 0.30 mmol) in xylene (50–60 mL) was heated to reflux while a stream of air was bubbled through the solution. Xylene was added periodically to avoid complete evaporation of the solution. After the time indicated in each case, the solution was evaporated, and the crude was chromatographed on silica gel eluting with ethyl acetate to afford compounds **6**.

**Method C:** A solution of NaOH 10% (2 mL) was added to the suitable dihydro derivative **6** (0.10 mmol). The suspension was stirred for 30 min at r.t., then it was diluted with 10 mL of water and extracted with ethyl acetate (3 × 20 mL). The resulting organic layer was evaporated and purified by chromatography on silica gel, eluting with ethyl acetate. The solid thus obtained was washed with ethyl ether to afford compounds **6**.

#### 3.5.1. 5-(*p*-(Dimethylamino)phenyl)-4-methyl-1*H*-1,8-diazaanthracene-2,9,10-trione (**5i**)

Method B: from 25 mg (0.07 mmol of **6i** was obtained 20 mg (80%) of **5i**. Reaction time 16 h;. [Found: C, 70.02; H, 4.72; N, 11.43. C_21_H_17_N_3_O_3_ requires C, 70.18; H, 4.77; N, 11.69]. Mp, >300 °C. ν*_max_* (KBr): 3440 (NH), 1676, 1664, 1656 (CO) cm^−1^. ^1^HNMR (CDCl_3_, 250 MHz) δ: 8.88 (d, 1H, *J* = 4.8 Hz, C_7_-H), 7.56 (d, 1H, *J* = 4.8 Hz, C_6_-H), 7.22 (d, 2H, *J* = 8.8 Hz, C_2′,6′_-H), 6.77 (d, 2H, *J* = 8.6 Hz, C_3′,5′_-H), 6.70 (d, 1H, *J* = 1.2 Hz, C_3_-H), 3.06 (s, 6H, N-CH_3_), 2.56 (d, 3H, *J* = 1.2 Hz, C_4_-CH_3_) ppm. ^13^C RMN (CDCl_3_, 63 MHz) δ: 182.21 (C-9), 177.14 (C-10), 167.94 (C-2), 160.39 (C-8a), 152.90 (C-7), 151.68 (C-5), 150.89 (C-4), 147.59 (C-4′), 138.64 (C-9a), 132.58 (C-6), 131.06 (C-1′), 129.56 (C-2′,C-6′), 128.96* (C-3), 128.58 (C-10a), 117.51 (C-4a), 111.84 (C-3′, C-5′), 40.35 (N-(CH_3_)_2_), 22.56 (C_4_-CH_3_) ppm.

#### 3.5.2. 4,6-Dimethyl-5-propyl-1*H*-1,8-diazaanthracene-2,9,10-trione (**5k**)

Method A: from 20 mg of **6k** (0.07 mmol) was obtained 18 mg (94%) of **5k**. Reaction time: 5 min. [Found: C, 68.69; H, 5.32; N, 9.43. C_17_H_16_N_2_O_3,_ requires C, 68.91; H, 5.44; N, 9.45]; Mp > 300 °C; ν*_max_* (KBr): 3421 (NH), 1654 (CO) cm^−1^; ^1^H NMR (CDCl_3_, 250 MHz) δ: 8,74 (s, 1H, C_7_-H), 6.70 (s, 1H, C_3_-H), 3.14 (t, 2H, *J* = 7.9, C_5_-CH_2_-CH_2_-CH_3_), 2.66 (s, 3H, C_4_-CH_3_), 2.49 (s, 3H, C_6_-CH_3_), 1.62 (m, 2H, C_5_-CH_2_-CH_2_-CH_3_), 1.10 (t, 3H, *J* = 7.3, C_5_-CH_2_-CH_2_-CH_3_) ppm. ^13^C RMN (CDCl_3_, 63 MHz) δ: 185.64 (C-9), 178.00 (C-10), 163.33 (C-2), 154.12 (C-5), 152.30 (C-4), 151.15 (C-7), 146.67 (C-8a), 140.67 (C-6), 136.13 (C-9a), 129.28 (C-3), 129.10 (C-10a), 117.95 (C-4a), 32.51 (C_5_-CH_2_), 30.10 (C_6_-CH_3_), 23.16 C_5_-CH_2_-CH_2_), 18.03 (C_4_-CH_3_), 15.28 (C_5_-CH_2_-CH_2_-CH_3_) ppm.

#### 3.5.3. 3,4,5-Trimethyl-1*H*-1,8-diazaanthracene-2,9,10-trione (**5r**)

Method C: From 20 mg of **6r** and 2 mL of NaOH 10% solution was obtained 74% of **5r**. Reaction time: 5 min. [Found: C, 67.23; H, 4.34; N, 10.79. C_15_H_12_N_2_O_3_ requires C, 67.16; H, 4.51; N, 10.44]. Mp > 300 °C; ν*_max_* (KBr): 3432 (NH), 1633 (CO) cm^−1^; ^1^H NMR (CDCl_3_, 250 MHz) δ: 9,72 (br s, 1H, NH), 8.81 (d, 1H, *J* = 4.9 Hz, C_7_-H), 7.49 (d, 1H, *J* = 4.9 Hz, C_6_-H), 2.82 (s, 3H, C_5_-CH_3_), 2.63 (s, 3H, C_4′_-CH_3_), 2.26 (s, 3H, C_3_-CH_3_) ppm. ^13^C RMN (CDCl_3_, 63 MHz) δ: 183.88 (C-9), 172.58 (C-10), 160.63 (C-2), 152.91 (C-7), 151.01 (C-5), 146.86 (C-8a), 145.91 (C-4), 136.99 (C-9a), 136.05 (C-3), 132.38 (C-6), 129.62 (C-10a), 117.70 (C-4a), 22.55 (C_5_-CH_3_), 18.00 (C_4_-CH_3_), 13.18 (C_3_-CH_3_) ppm.

#### 3.5.4. 4-Ethyl-5-methyl-1*H*-1,8-diazaanthracene-2,9,10-trione (**5s**)

Method B: From 16 mg of **6r** was obtained 82% of **5s**. Reaction time: 3 h. [Found: C, 67.12; H, 4.32; N, 10.43. C_15_H_12_N_2_O_3_ requires C, 67.16; H, 4.51; N, 10.44]. Mp, 222–224 °C (ethyl ether/ethanol, 8:2). ν*_max_* (KBr): 3428 (NH), 1546 (CO) cm^−1^; ^1^H NMR (CDCl_3_, 250 MHz) δ: 9,86 (br s, 1H, NH), 8.85 (d, 1H, *J* = 4.9 Hz, C_7_-H), 7.54 (d, 1H, *J* = 4.9 Hz, C_6_-H), 6.77 (s, 1H, C_3_-H), 3.14 (c, 2H, *J* = 7.3 Hz, C_4_-CH_2_-CH_3_), 2.86 (s, 3H, C_5_-CH_3_), 1.29 (t, 3H, *J* = 7.3 Hz, C_4_-CH_2_-CH_3_) ppm. ^13^C RMN (CDCl_3_, 63 MHz) δ: 183.19 (C-9), 177.08 (C-10), 160.53 (C-2), 157.44 (C-4), 153.19 (C-7), 151.44 (C-5), 146.98 (C-8a), 139.13 (C-9a), 132.76 (C-6), 129.45 (C-10a), 126.96 (C-3), 116.57 (C-4a), 27.90 (CH_2_-CH_3_), 22.94 (C_5_-CH_3_), 13.79 (CH_2_-CH_3_).

#### 3.5.5. 5-Methyl-4-propyl-1*H*-1,8-diazaanthracene-2,9,10-trione (**5t**)

Method A: from 55 mg (0.19 mmol) of **6t** and 99 mg (0.97 mmol) of activated 85% MnO_2_ was obtained 92% of **5t**. Reaction time: 30 min. [Found: C, 67.88; H, 4.86; N, 9.75. C_16_H_14_N_2_O_3_ requires C, 68.08; H, 5.00; N, 9.92]. Mp: 236–238 °C. ν*_max_* (KBr): 3431 (NH), 1648 (CO) cm^−1^. ^1^H NMR (CDCl_3_, 250 MHz) δ: 9.86 (br s, 1H, NH), 8.84 (d, 1H, *J* = 4.9 Hz, C_7_-H), 7.53 (d, 1H, *J* = 4.8 Hz, C_6_-H), 6.73 (s, 1H, C_3_-H), 3.06 (t, 2H, *J* = 7.4 Hz, C_4_-CH_2_), 2.86 (s, 3H, C_5_-CH_3_), 1.66 (m, 2H, *J* = 7.4 Hz, C_4_-CH_2_-CH_2_), 1.07 (t, 3H, *J* = 7.4 Hz, C_4_-CH_2_-CH_2_-CH_3_) ppm. ^13^C RMN (CDCl_3_, 63 MHz) δ: 183.08 (C-9), 176.92 (C-10), 160.61 (C-2), 155.80 (C-4), 153.08 (C-7), 151.38 (C-5), 147.07 (C-8a), 139.26 (C-9a), 132.61 (C-6), C-10a was not observed, 127.78 (C-3), 116.61 (C-4a), 36.50 (C_4_-CH_2_), 22.84 (C_5_-CH_3_), 22.76 (C_4_-CH_2_-CH_2_), 13.99 (C_4_-CH_2_-CH_2_-CH_3_) ppm.

#### 3.5.6. 5-Methyl-1-(4-tolyl)-1,8-diazaanthracene-2,9,10-trione (**5v**)

Method A: from 30 mg 0.09 mmol) of **6v** and 10 mg (0.09 mmol) of activated 85% MnO_2_ was obtained 3 mg (94%) of **5v** Reaction time: 24 h. Yield, 94%. [Found: C, 72.42; H, 4.36; N, 8.21. C_20_H_14_N_2_O_3_ requires C, 72.72; H, 4.27; N, 8.48]. Mp, 260–262 °C; ν*_max_* (KBr) 1662 (CO) cm^−1^. ^1^H NMR (CDCl_3_, 250 MHz) δ: 8.83 (d, 1H, *J* = 4.9 Hz, C_7_-H), 8.29 (d, 1H, *J* = 9.6 Hz, C_4_-H), 7.20 (d, 1H, *J* = 5.0 Hz, C_6_-H), 7.31 (d, 2H, *J* = 8.0 Hz, C_3′,5′_-H), 7.08 (d, 2H, *J* = 8.3 Hz, C_2′,6′_-H), 6.96 (d, 1H, *J* = 9.6 Hz, C_3_-H), 2.48 (s, 3H, C_5_-CH_3_), 2.45 (s, 3H, C_4′_-CH_3_) ppm. ^13^C RMN (CDCl_3_, 63 MHz) δ: 179.87 (C-9), 179.30 (C-10), 162.16 (C-2), 153.49 (C-7), 150.23 (C-5), 147.64 (C-8a), 138.73 (C-1′), 135.63 (C-9a), 135.26 (C-4), 130.78 (C-4′), 130.70 (C-6), 129.87 (C-3′, C-5′), 128.69 (C-10a), 126.87 (C-2′,C-6′), 126.42 (C-3), 117.81 (C-4a), 22.88 (C-5-CH_3_), 21.54 (C_4′_-CH_3_) ppm.

### 3.6. Diels–Alder Reaction of Quinone ***8b*** and 2,3-Dimethyl-1,3-butadiene

Method A: A solution of quinone **8b** (223 mg, 1.20 mmol) and 2,3-dimethyl-1,3-butadiene (0.15 mL, 1.30 mmol) in ethyl acetate was heated at 100 °C for 12 h in a sealed tube. The solution was cooled and evaporate under vacuum. The residue was chromatographed on silica gel eluting with dichloromethane/ethyl acetate (6:4), to afford 153 mg of (±)-(5aS*,9aR*)-4,7,8-trimethyl-5a,6,9,9a-tetrahydrobenzo[*g*]quinoline-2,5,10(1*H*)-trione **14** (48%). [Found: C, 70.21; H, 6.15; N, 5.07. C_16_H_17_NO_3_ requires: C, 70.83; H, 6.32; N, 5.16]. Mp, 285–288 °C (AcOEt). ν*_max_* (KBr): 3200–2800 (NH), 1660 (CO) cm^−1^. ^1^H NMR (CDCl_3_, 300 MHz) δ: 9.80 (br s, 1H, NH), 6.64 (d, 1H, *J* = 1.2 Hz, H-3); 3.33 (m, 2H, C_8a_-H and C_10a_-H), 2.53 (d, 3 H, *J* = 1.2 Hz, C_4_-CH_3_), 2.42 (m, 2H, C_5_-H_ax_ and C_8_-H_ax_), 2.15 (m, 2H, C_5_-H_eq_ and C_8_-H_eq_), 1.64 (s, 6H, C_6,7_-CH_3_) ppm. ^13^C NMR (CDCl_3_, 75 MHz) δ: 196.18 (C-9), 192.68 (C-10), 160.18 (C-2), 152.15 (C-4), 140.18 (C-9a), 127.86 (C-3), 123.68 (C-6), 123.18 (C-7), 118.20 (C-4a), 47.76 (C-8a), 46.09 (C-9a), 30.70 (C-5), 30.46 (C-8), 21.98 (C_4_-CH_3_), 18.82 (C_6_-CH_3_ and C_7_-CH_3_) ppm.

Method B: A solution of quinone **8b** (245 mg, 1.30 mmol) and 2,3-dimethyl-1,3-butadiene (0.16 mL, 1.40 mmol) in ethyl acetate was heated at 120 °C for 88 h in a sealed tube. The solution was cooled and evaporated under vacuum. The residue was chromatographed on silica gel eluting with a gradient from dichloromethane to dichloromethane/ethyl acetate (6:4), to afford a mixture of compounds **14** and **7g** (**14**:**7g** = 3.5:1). A solution of this mixture (90 mg, 0.22 mmol) and DDQ (340 mg, 1.50 mmol) in dry benzene (130 mL) was refluxed under nitrogen atmosphere for 7 h. The reaction mixture was evaporated, and the crude was chromatographed on silica gel eluting with dichloromethane/ethyl acetate (1:1) yielding 70 mg of compound **7g** (68%). [Found: C, 71.50; H, 4.97; N, 5.25. C_16_H_13_NO_3_ requires: C, 71.90; H, 4.90; N, 5.24]. Mp > 310 °C (AcOEt/CH_2_Cl_2_ 1:1). ν*_max_* (KBr): 3320, 3630–3070 (NH), 1685, 1670, 1660 (CO) cm^−1^. ^1^H NMR (CDCl_3_, 250 MHz) δ: 8.81 (s, 1H, C_8_-H), 8.09 (s, 1H, C_5_-H), 6.67 (d, 1H, *J* = 1. Hz, C_3_-H), 2.70 (d, 3 H, *J* = 1 Hz, C_4_-CH_3_), 2.45 (s, 3 H, C_6_- CH_3_), 2.43 (s, 3 H, C_7_-CH_3_) ppm.

### 3.7. Diels–Alder Reaction of Quinone ***8b*** and Anthracene—Synthesis of 4-Methyl-6,11-[1,2]Benzenonaphtho[2,3-g]Quinoline-2,5,12(1H,6H,11H)-trione ***16***

A solution of quinone **8b** (17 mg, 0.94 mmol) and anthracene (184 mg, 1.03 mmol) in chloroform (100 mL) was refluxed for 16 h. The solution was cooled and evaporated under vacuum. The residue was chromatographed on silica gel eluting with a gradient from dichloromethane to dichloromethane/ethyl acetate (7:3), to yield 163 mg (49%) of compound **16**. [Found: C, 78.54; H, 4.21; N, 3.88. C_24_H_15_NO_3_ requires: C, 78.89; H, 4.14; N, 3.83]. Mp, 306–308 °C (AcOEt). ν*_max_* (KBr): 1650, 1630 (CO) cm^−1^. ^1^H NMR (d_6_-DMSO, 300 MHz) δ: 10.86 (br s, 1H, NH), 7.52 (m, 4H, anthracene), 7.05 (m, 4H, anthracene), 6.46 (d, 1H, *J* = 1.2 Hz, H-3); 5.96 and 5.94 (2s, 2H, C_9′_-H and C_10′_-H), 2.46 (d, 3H, *J* = 1.2 Hz, C_4_-CH_3_) ppm. ^13^C NMR (d_6_-DMSO, 75 MHz) δ: 180.45 (C-8), 175.97 (C-5), 161.92 (C-2), 155.46 (C-6), 151.64 (C-4), 149.46 (C-7), 143.08 (C-4a′, C-8a′, C-9a′, C-10a′), 138.58 (C-8a), 126.39 (C-3), 125.78 and 125.71 (C1′, C-4′, C-5′, C-8′), 124.55 and 124.39 (C-2, C-3, C-6, C-7), 113.25 (C-4a), 47.72 (C-9′), 47.05 (C-10′), 22.25 (C_4_-CH_3_) ppm.

### 3.8. Diels–Alder Reaction of Quinone ***8b*** and Cyclopentadiene

A solution of quinone **8b** (370 mg, 1.90 mmol) and cyclopentadiene (0.32 mL, 3.90 mmol) in chloroform (130 mL) was heating at 110 °C for 18 h in a sealed tube. The solution was cooled and evaporated under vacuum. The residue was chromatographed on silica gel eluting with a gradient from dichloromethane to dichloromethane/ethyl acetate (1:1), to yield 40 mg of compound **17** (23%) and 118 mg of compound **18** (71%).

#### 3.8.1. 9,10-Dihydroxy-5,8-methano-4-methyl-5,8-dihydro-1*H*-1-azaanthracen-2-one (**17**)

[Found: C, 70.29; H, 4.98; N, 5.25. C_15_H_13_NO_3_ requires C, 70.58; H, 5.13; N, 5.49]. Mp, 323–326 °C (AcOEt). ν*_max_* (KBr): 3325, 3300–2600 (OH), 1625 (CO) cm^−1^. ^1^H NMR (d_6_-DMSO, 300 MHz) δ: 9.68 (br s, 1H, OH), 9.21 (br s, 1H, OH), 8.81 (br s, 1H, NH), 6.72 (m, 2H, H-6 and H-7), 6.11 (s, 1H, H-3), 4.30 (d, 2H, *J* = 10 Hz, H-5, H-8); 2.40 (s, 3H, C_4_-CH_3_), 2.00 (m, 2H, C_5_-CH_2_-C_8_) ppm. ^13^C NMR (d_6_-DMSO, 75 MHz) δ: 160.84 (C-2), 150.21 (C-10), 142.82 (C-6), 141.69 (C-9), 141.32 (C-7), 137.79 (C-8a), 130.76 (C-4), 129.16 (C-10a), 127.85 (C-9a), 119.29 (C-3), 108.86 (C-4a), 66.38 (C_5_-CH_2_-C_8_), 46.80 (C-5), 45.96 (C-8), 24.39 (C_4_-CH_3_) ppm.

#### 3.8.2. 5,8-Methano-4-methyl-5,8-dihydro-1*H*-1-azaanthracen-2,9,10-trione (**18**)

[Found: C, 70.89; H, 4.18; N, 5.35. C_15_H_11_NO_3_ requires C, 71.14; H, 4.38; N, 5.53]. Mp, 323–236 °C (AcOEt). ν*_max_* (KBr): 3200–2800 (NH), 1655, 1650, 1640 (CO) cm^−1^. ^1^H NMR (CDCl_3_, 300 MHz) δ: 10.36 (br s, 1H, NH), 6.88 (m, 2H, H-6 and H-7), 6.59 (d, 1H, *J* = 1.2 Hz, H-3), 4.20 (br s, 2H, H-5, H-8); 2.58 (d, 3H, *J* = 1.2 Hz, C_4_-CH_3_), 2.36 (m, 2H, C_5_-CH_2_-C_8_) ppm. ^13^C NMR (CDCl_3_, 75 MHz) δ: 181.02 (C-9), 175.37 (C-10), 165.58 (C-10a), 161.07 (C-2), 157.09 (C-8a), 151.08 (C-4), 142.37 (C-6), 142.31 (C-7), 139.25 (C-9a), 125.69 (C-3), 113.42 (C-4a), 73.41 (C_5_-CH_2_-C_8_), 48.98 (C-5), 48.20 (C-8), 22.09 (C_4_-CH_3_) ppm.

### 3.9. Bioassays

Cells (Sigma-Aldrich, Madrid, Spain or ATCC, Manassas, Virginia, USA) were maintained in logarithmic growth phase in Eagle’s essential medium with Earle’s balanced salts, 2.0 mM L-glutamine, non-essential amino acids and no sodium bicarbonate (EMEM/NEAA), supplemented with 10% sodium bicarbonate 10–2 fetal calf serum (FCS) and 0.1 g/L penicillin G + streptomycin sulphate.

To determine and compare the antitumor activity of these compounds, simple screening procedures were performed using an adapted form of the method described by Bergerom et al. [56,57] The tumor cells used were P-388 (BDA/2 mouse lymphoid neoplasm suspension culture), A-549 (human lung carcinoma monolayer culture), HT-29 (human colon carcinoma monolayer culture), MEL-29 (human melanoma monolayer culture).

P-388 cells were seeded in 16 mm wells at 1 × 10^4^ cells per well in aliquots to 1 mL of 5FCS MEM containing the indicated drug concentration. Separately, a batch of drug-free cultures was seeded as a growth control to ensure that the cells remained in log phase of growth. All determinations were performed in triplicate. After three days of incubation at 37 °C, 10% CO_2_ in an atmosphere of 98% humidity, the approximate IC_50_ was determined by comparing growth in the drug wells and growth in the control wells.

A-549, HT-29 and MEL-28 cells were seeded in 16 mm wells at 2 × 10^4^ cells per well in 1 mL aliquots of MEM 10FCS containing the indicated drug concentration. Separately, a drug-free culture batch was seeded as a growth control to ensure that the cells remained in log phase of growth. All determinations were performed in duplicate. After three days of incubation at 37 °C, 10% CO_2_ in an atmosphere of 98% humidity, the wells were stained with 0.1% Crystal Violet. The IC_50_ value was determined by comparing the growth in the drug wells and the growth in the control wells.

## 4. Conclusions

The synthesis of libraries of derivatives of the 1,8-diazaanthracene-2,9,10-trione, including their 5,8-dihydro derivatives, 1,8-diazaanthracene-2,7,9,10-tetraone and 1-azaanthracene-2,9,10-trione frameworks, structurally related to the diazaquinomycin and marcanine families of natural products, was achieved using Diels–Alder strategies. Many of them were found to have potent and selective cytotoxicity against some solid tumors. In particular, 1,8-diazaanthracene-2,9,10-triones and their 5,8-dihydro derivatives were particularly active against a human lung cancer cell line. The use of a 1-azadiene comprising a SAMP-related chiral auxiliary allowed the enantioselective synthesis of two representatives of chiral 5-substituted 1,8-diazaanthracene-2,9,10-triones, and their study showed that their cytotoxicity was not enantiospecific. The following targets and processes were discarded as explanations of the mechanism of action of our compounds: thymidylate synthase, dihydrofolate reductase, topoisomerases I and II, ADN polymerases, ARN polymerases, DNA, RNA and protein synthesis. Further mechanistic work will be needed to characterize the mechanism of action of these interesting compounds.

## Figures and Tables

**Table 1:** Structures of 1-azadienes **9**.

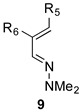					
**Comp.**	**R_5_**	**R_6_**	**Comp.**	**R_5_**	**R_6_**
**a**	H	H	**f**	Ph	H
**b**	H	Me	**g**	4-Me_2_NC_6_H_4_	H
**c**	H	Et	**h**	Et	Me
**d**	H	*n*Bu	**i**	*n*Pr	Me
**e**	Me	H	**j**	*n*Bu	Me

**Table 2:** Structures of quinones **8**.

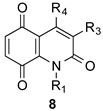							
**Comp.**	**R_1_**	**R_3_**	**R_4_**	**Comp.**	**R_1_**	**R_3_**	**R_4_**
**a**	H	H	H	**h**	*p-*Tol	H	H
**b**	H	H	Me	**i**	H	*p*-Tol	H
**c**	Me	H	Me	**j**	H	H	*n*Pr
**d**	H	Me	Me	**k**	H	Me	H
**e**	H	Bn	Me	**l**	H	Et	H
**f**	H	H	Et	**m**	H	Ph	H
**g**	H	H	(CH_2_)_2_Ph	**n**	H	CO_2_Et	H

**Table 3:** Scope and yields of the hetero Diels–Alder reactions.

Comp.	R_1_	R_3_	R_4_	R_5_	R_6_	Method *^a^*	Yield, %		
							5	6	10
**a**	H	H	H	H	H	A	81	0	8 *^b^*
**b**	H	H	H	H	Et	A	70	0	0 *^b^*
**c**	H	H	Me	H	H	A	70	0	14 *^b^*
**d**	H	H	Me	H	Me	A (B)	48 (88)	0	20 *^b^ *(0) * ^c^*
**e**	H	H	Me	H	Et	A	42	0	33 *^b^*
**f**	H	H	Me	H	*n*Bu	A (C)	30 (68)	0	50 (8) *^f^*
**g**	H	H	Me	Me	H	A (C)	0	51 (53)	35* ^b^* (15) * ^f^*
**h**	H	H	Me	Ph	H	A	0	34	65 *^b^*
**i**	H	H	Me	4-Me_2_NC_6_H_4_	H	A	0	11	61 *^e^*
**j**	H	H	Me	Et	Me	A	0	47	45 *^b^*
**k**	H	H	Me	*n*Pr	Me	A	0	32	40
**l**	H	Me	Me	H	Me	A	76	0	11
**m**	H	Bn	Me	H	Me	B	68	0	0
**n**	H	H	Et	H	Me	A	70	0	9
**o**	H	H	(CH_2_)_2_Ph	H	Me	A (B)	49 (82)	0 (0)	15 (0) *^c^*
**p**	*p*-Tol	H	H	H	Me	D	52	0	9
**q**	H	*p*-Tol	H	H	Me	B	94	0	0
**r**	H	Me	Me	Me	H	A (B)	0	32 (70)	42 (6) *^c^*
**s**	H	H	Et	Me	H	A (B)	0	29 (84)	59 (0) *^c^*
**t**	H	H	*n*Pr	Me	H	A(B)	0	32 (92)	50 (0) *^c^*
**u**	H	Me	*n*Pr	*n*Pr	Me	B	24	46	0 *^g^*
**v**	*p*-Tol	H	H	Me	H	B	0	80	0
**w**	H	*p-*Tol	H	Me	H	B	64	0	0
**x**	H	Me	H	H	Me	A	50	0	0 *^d^*
**y**	H	Et	H	H	Me	A	45	0	0 *^d^*
**z**	H	Ph	H	H	Me	A	50	0	0 *^d^*
**aa**	H	Me	H	Me	H	D	46	6	0 *^d^*
**ab**	H	Et	H	Me	H	A (B)	18 (44)	27 (0)	7 (0) *^d^*
**ac**	H	Ph	H	Me	H	D	40	0	20 *^d^*
**ad**	H	CO_2_Et	H	H	Me	A	59	0	0 *^d^*
**ae**	H	H	Me	Et	H	A (C)	0	28 (70)	25 (8) *^f^*
**af**	H	H	Me	nPr	H	A (C)	0	27 (62)	65 (8) *^f^*
**ag**	H	H	Me	nBu	H	A (C)	0	30 (65)	50 (8) *^f^*

*^a^* Method A: Reaction in chloroform at room temperature. Method B: Compound **8** was supported on silica gel, which was eluted with a chloroform solution of compound **9**. Method C: Reaction in chloroform in the presence of a chloroformyl polystyrene resin. Method D: reaction according to method B, followed by oxidation with activated manganese dioxide. *^b^* Data obtained from reference [33]. *^c^* Data obtained from reference [34]. *^d^* Data obtained from reference [36]. *^e^* Data obtained from reference [46]. *^f^* Data obtained from reference [35]. *^g^*  Data obtained from reference [44].

**Table 4:** Scope and yields obtained in the dehydrogenation of compounds **6**.

Comp.	R_1_	R_3_	R_4_	R_5_	R_6_	Method *^a^*	Yield, %
**5g**	H	H	Me	Me	H	A (B)	88 (90) *^b^*
**5h**	H	H	Me	Ph	H	A (B)	83 (70) *^b^*
**5i**	H	H	Me	4-Me_2_NC_6_H_4_	H	B	80
**5j**	H	H	Me	Et	Me	A (B)	94 (95) *^b^*
**5k**	H	H	Me	*n*Pr	Me	A	94
**5r**	H	Me	Me	Me	H	C	74 *^c^*
**5s**	H	H	Et	Me	H	B	82
**5t**	H	H	*n*Pr	Me	H	A	92
**5u**	H	Me	*n*Pr	*n*Pr	Me	A	63 *^c^*
**5v**	*p*-Tol	H	H	Me	H	A	94

*^a^* Method A: MnO_2_, CHCl_3_, rt, 10 min. Method B: Xylene, reflux, 60–120 h. Method C: 10% aq. NaOH, rt. *^b^* Data obtained from reference [33]. *^c^* Data obtained from reference [44].

**Table 5:** Structures and yields of compounds **7**.

Comp.	R_1_	R_4_	R_6_	R_7_	R_8_	Conditions	Yield %
**7a**	H	H	H	H	H	CHCl_3_, 95 °C, 2 h (sealed tube)	79 *^a,d^*
**7b**	Me	Me	H	H	H	AcOEt, 100 °C, 24 h (sealed tube)	81 *^a^*
**7c**	H	Me	Me	H	H	AcOEt, 120 °C, 24 h (sealed tube)	64 *^a,c^*
**7d**	H	Me	H	H	Me	AcOEt, 120 °C, 14 h (sealed tube)	60 *^c^*
**7e**	H	Me	Me	H	Me	AcOEt, 120 °C, 1 h, then xylene, 150 °C, 36 h	57 *^a^*
**7f** (marcanine A)	H	Me	H	H	H	AcOEt, 120 °C, 24 h (sealed tube)	81 *^b^*

*^a^* Data obtained from reference [49]. *^b^* Using 1-methoxy-1,3-butadiene as diene. *^c^* Isolated as a 3:1 mixture of **7c** and its 7-Me regioisomer, from which a sample for biology was obtained by slow column chromatography on silica gel, eluting with 6:4 dichloromethane-ethyl acetate. *^d^* Using 1-trimethylsilyloxy-1,3-butadiene as the diene.

## Data Availability

Data are contained within the article and supplementary materials.

## Supplementary Materials

The following supporting information can be downloaded at: https://www.mdpi.com/article/10.3390/molecules29020489/s1, Table S1: Cytotoxicity of quinolinetriones **8**. Table S2: Cytotoxicity of 1-azaanthracenetrione derivatives **7** and the related non-aromatic intermediate **14**. Table S3: Cytotoxicity of 5,8-dihydro-1-azaanthracenetrione derivatives **15**, **16** and **18**. Table S4: Cytotoxicity of compounds **5**. Table S5: Cytotoxicity of compounds **6**. Table S6: Cytotoxicity of the diazoquinomycin derivatives **1**. Copies of representative NMR spectra.
