# Supplementary material for: Structure-Antitumor Activity Relationships of Aza- and Diaza-Anthracene-2,9,10-Triones and Their Partially Saturated Derivatives
Source: Molecules. 2024 Jan 18;29(2):489. doi: 10.3390/molecules29020489 (PMC10819329; doi:10.3390/molecules29020489)
Supplement: Supplementary file 1 [file molecules-29-00489-s001.zip › molecules-2825391-supplementary.pdf]

Supporting Information

Structure-antitumour activity relationships of aza- and diaza- anthracene-2,9,10-triones and their partially saturated derivatives

Carmen Avendaño[1,*], Pilar López-Alvarado[1], José María Pérez[1], Miguel Ángel Alonso[1], Eva Pascual-Alfonso[1], Miriam Ruiz-Serrano[1], and J. Carlos Menéndez[1,*]

Unidad de Química Orgánica y Farmacéutica, Departamento de Química en Ciencias Farmacéuticas, Facultad de Farmacia, Universidad Complutense. 28040 Madrid, Spain. avendano@ucm.es (C.A.); alvarado@ucm.es (P.L-A); jmpnunez@gmail.con (J.M.P.); alonrizal@hotmail.com (M.Á.A.); epascual24@hotmail.com (E.P.-A.); miriamruizserrano@ucm.es (M.R.).; josecm@ucm.es (J.C.M.).

Table of contents

1. Cytotoxicity numerical data S2
2. Copies of selected spectra S8

1. Cytotoxicity numerical data

Table S1: Cytotoxicity of quinolinetriones **8**

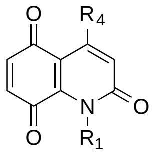

8

Comp.	R_1	R_3	R_4	IC_{50} (μM)		
				**P-388**	**A-549**	**HT-29**
**8b**	H	H	Me	10.58	13.22	13.22
**8c**	Me	H	Me	1.23	4.92	4.92
**8d**	H	Me	Me	9.85	9.85	9.85
**8f**	H	H	Et	9.85	9.85	24.63
**8g**	H	H	$(CH_2)_2Ph$	8.87	3.54	17.92
**8j**	H	H	*n*Pr	11.51	11.52	23.04
**8k**	H	Me	H	4.18	8.37	8.37
**8l**	H	Et	H	2.09	8.37	8.37
**8n**	H	CO_2Et	H	10.46	41.84	41.84

Table S2: Cytotoxicity of 1-azaanthracenetrione derivatives **7** and the related non-aromatic intermediate **14**

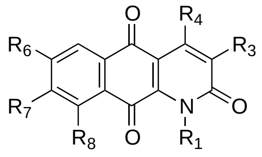

7

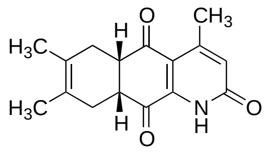

14

**Comp.**	**R_1**	**R_3**	**R_4**	**R_6**	**R_7**	**R_8**	**IC_{50} (μM)**			
							**P-388**	**A-549**	**HT-29**	**MEL-28**
**7a**	H	H	H	H	H	H	0.50	0.01	0.10	--
**7b**	Me	H	Me	H	H	H	3.95	1.97	3.95	4.74
**7c**	H	H	Me	Me	H	H	1.97	0.19	0.98	--
**7d**	H	H	Me	H	H	Me	39.52	39.52	39.52	--
**7e**	H	H	Me	Me	H	Me	9.36	18.72	37.44	--
**7f (marcanine A)**	H	H	Me	H	H	H	10.46	1.04	2.09	--
**7g**	H	H	Me	Me	Me	H	>75	>75	>75	--
**Marcanine B**	Me	OMe	Me	H	H	H	--	0.35	2.12	-- [1]
**Marcanine C**	Me	OMe	CH_2OH	H	H	H	--	1.00	0.33	-- [1]
**Marcanine G**	Me	OMe	Ac	H	H	H	--	14.87	--	-- [2]
**14**	H	H	Me	Me	Me	H	3.69	0.92	3.69	3.69

[1] Soonthornchareonnon, N.; Suwanborirux, K.; Bavovada, R.; Patarapanich, C.; Cassady, J.M., New cytotoxic 1-azaanthraquinones and 3-aminonapthoquinone from the stem bark of *Goniothalamus marcanii*. *J. Nat. Prod.* **1999**, *62*, 1390-1394.

[2] Thanuphol, P.; Asami, Y.; Shiomi, K.; Wongnoppavich, A-; Tuchinda, P.; Soonthornchareonnon, N. Marcanine G, a new cytotoxic 1-azaanthraquinone from the stem bark of *Goniothalamus marcanii* Craib. *Nat. Prod. Res.* **2018**, *32*, 1682-1689.

Table S3: Cytotoxicity of 5,8-dihydro-1-azaanthracenetrione derivatives **15**, **16** and **18**

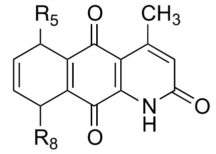

**Comp.**	**R5**	**R8**	**IC50 (μM)**			
			**P-388**	**A-549**	**HT-29**	**MEL-28**
**15**	H	Me	1.96	1.96	1.96	1.96
**16**	1,2-Phenylene		1.41	1.41	2.83	--
**18**	CH_2		7.90	7.90	7.90	--

Table S4: Cytotoxicity of compounds **5**

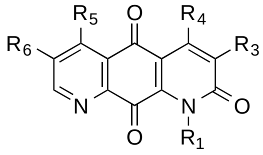

5

Comp.	R1	R3	R4	R5	R6	IC50 (μM)				
						**P-388**	**A-549**	**HT-29**	**MCF-7**	**MEL-28**
**a**	H	H	H	H	H	2.21	0.44	4.42	0.44	-
**b**	H	H	H	H	Et	0.39	0.08	0.39	-	0.19
**c**	H	H	Me	H	H	5.0	0.5	5.0	-	0.5
**d**	H	H	Me	H	Me	0.98	0.98	0.19	-	-
**e**	H	H	Me	H	Et	1.86	0.37	0.74	-	-
**f**	H	H	Me	H	*n*Bu	0.04	0.34	0.34	0.34	-
**g**	H	H	Me	Me	H	3.93	0.39	0.78	-	-
**h**	H	H	Me	Ph	H	1.58	0.79	0.79	-	-
**i**	H	H	Me	4-$Me_2NC_6H_4$	H	2.78	2.78	2.78	-	-
**j**	H	H	Me	Et	Me	3.54	1.77	0.88	-	-
**k**	H	H	Me	*n*Pr	Me	0.84	0.84	1.68	1.68	-
**l**	H	Me	Me	H	Me	3.73	0.37	1.87	-	0.37
**m**	H	Bn	Me	H	Me	14.50	0.29	2.90	-	0.29
**n**	H	H	Et	H	Me	3.73	0.17	0.75	-	0.37
**o**	H	H	$(CH_2)_2Ph$	H	Me	2.91	0.15	1.45	-	0.15
**p**	*p*-Tol	H	H	H	Me	0.76	0.76	0.76	-	0.76
**q**	H	*p*-Tol	H	H	Me	1.52	0.30	1.52	-	0.30
**r**	H	Me	Me	Me	H	9.26	0.37	1.85	-	0.37
**s**	H	H	Et	Me	H	3.70	0.37	0.93	-	0.37
**t**	H	H	*n*Pr	Me	H	8.87	0.35	3.54	-	0.35
**u**	H	Me	*n*Pr	*n*Pr	Me	1.78	0.44	0.44	-	0.88
**v**	*p*-Tol	H	H	Me	H	3.03	1.51	0.30	-	0.30
**w**	H	*p*-Tol	H	Me	H	3.03	0.30	0.36	-	0.30
**x**	H	Me	H	H	Me	0.84	0.21	0.84	-	0.21
**y**	H	Et	H	H	Me	0.84	0.11	0.84	-	-
**z**	H	Ph	H	H	Me	0.79	0.16	0.79	-	0.16
**aa**	H	Me	H	Me	H	1.05	0.42	1.05	0.42	-
**ab**	H	Et	H	Me	H	1.05	0.10	1.05	0.21	-
**ac**	H	Ph	H	Me	H	0.50	0.42	2.09	0.42	-
**ad**	H	CO_2Et	H	H	Me	2.09	2.09	4.18	-	-
**ah**	H	H	Ac	H	Me	8.87	3.54	8.87	-	3.54

Table S5: Cytotoxicity of compounds **6**

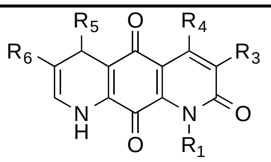

6

Comp.	R1	R3	R4	R5	R6	IC50 (μM)			
						**P-388**	**A-549**	**HT-29**	**MEL-28**
**g**	H	H	Me	Me	H	0.97	0.39	0.78	-
**h**	H	H	Me	Ph	H	0.78	0.78	0.16	-
**i**	H	H	Me	4-$(Me_2N)C_6H_4$	H	55.40	13.85	1.38	-
**j**	H	H	Me	Et	Me	3.35	0.83	1.67	-
**k**	H	H	Me	*n*Pr	Me	1.67	0.33	1.67	0.83
**r**	H	Me	Me	Me	H	9.26	0.37	1.85	0.37
**s**	H	H	Et	Me	H	3.70	0.93	1.85	1.85
**t**	H	H	*n*Pr	Me	H	1.76	0.42	1.76	0.42
**u**	H	Me	*n*Pr	*n*Pr	Me	1.76	0.88	1.76	1.76
**v**	*p*-Tol	H	H	Me	H	3.01	3.01	3.01	3.01
**ae**	H	H	Me	Et	H	0.37	0.37	0.37	0.37
**af**	H	H	Me	nPr	H	0.44	0.44	0.44	0.44
**ag**	H	H	Me	nBu	H	0.34	0.34	0.34	0.34
**ai**	Me	H	Me	(*R*)-Me	H	10.19	0.47	1.02	0.47
**aj**	Me	H	Me	(*S*)-Me	H	10.19	1.02	2.04	1.02

Table 6: Biological activity of the diazoquinomycin derivatives **1**

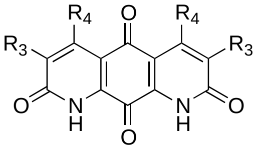

1

**Comp.**	**R$_3$**	**R$_4$**	**IC$_{50}$ (μM)**			
			**P-388**	**A-549**	**HT-29**	**MEL-28**
**1a (DAQA)**	Me	*n*Pr	5.65	2.62	5.65	5.65
**1b**	Me	Et	0.15	0.15	0.15	0.15
**1c**	H	Me	0.16	0.16	0.16	0.16
**1d**	Me	H	0.18	0.09	0.18	0.18
**1e**	Et	H	0.17	0.17	0.17	0.17

2. Copies of selected spectra

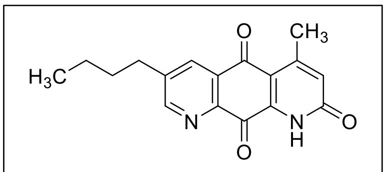
5f

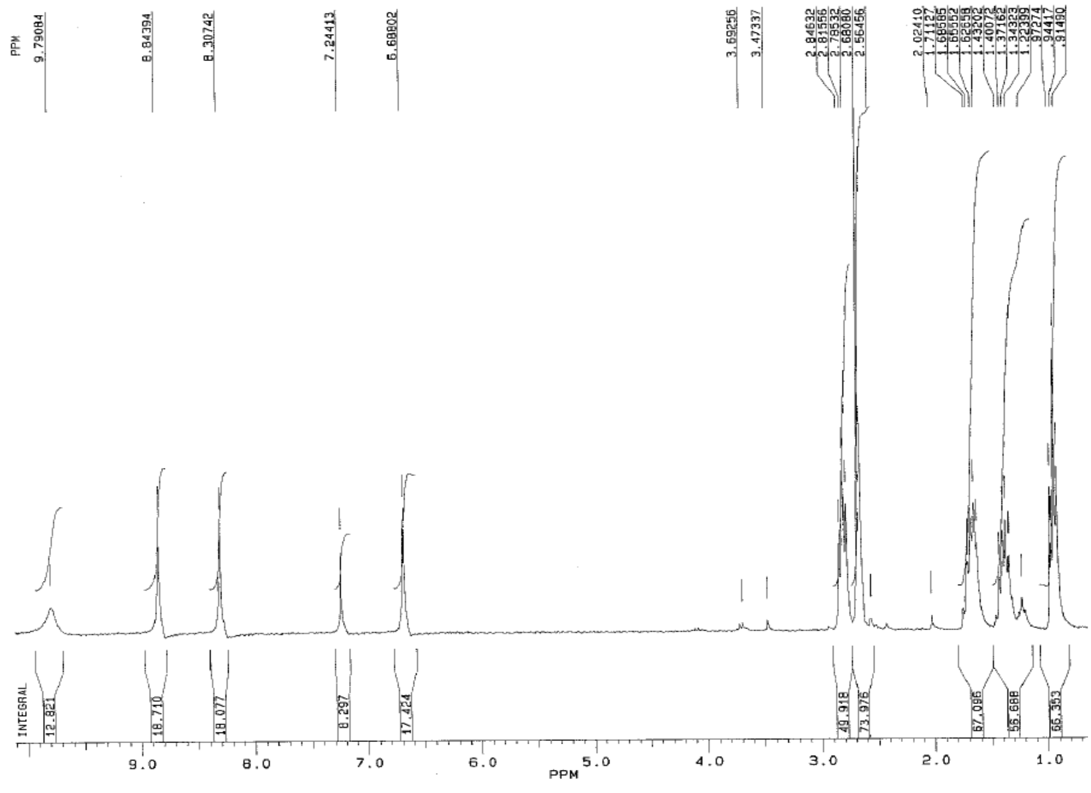

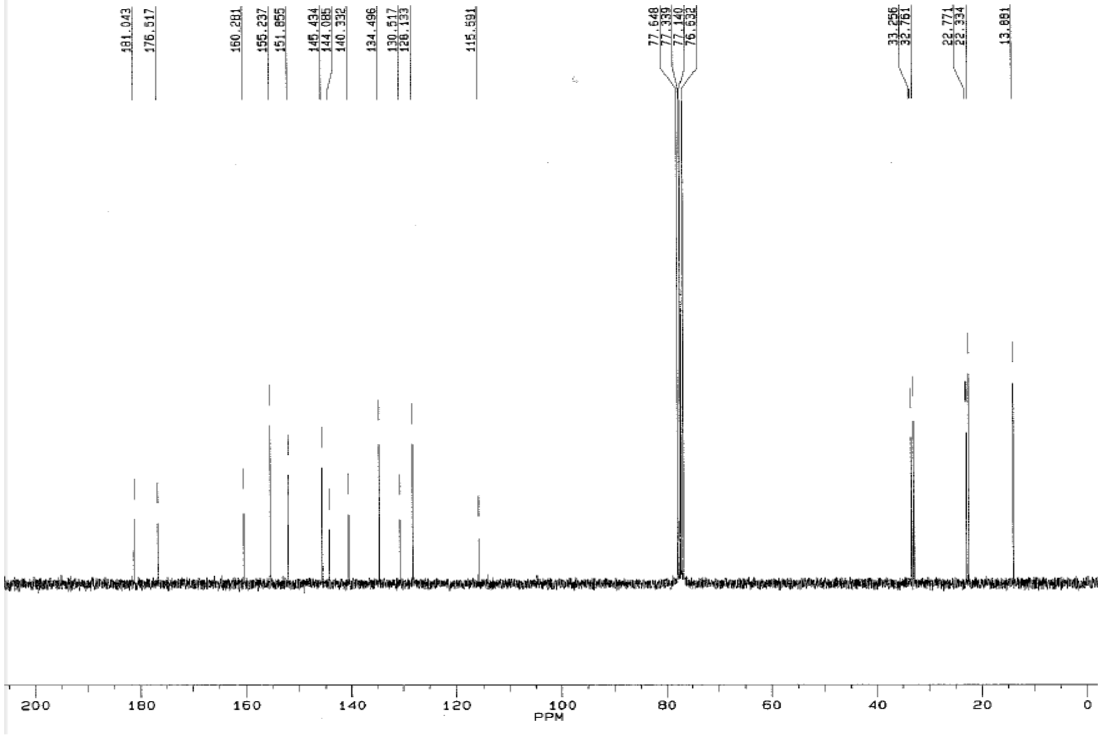

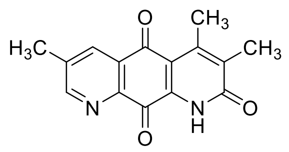
O
CH3
H3C
CH3
N
NH
O
O

5l

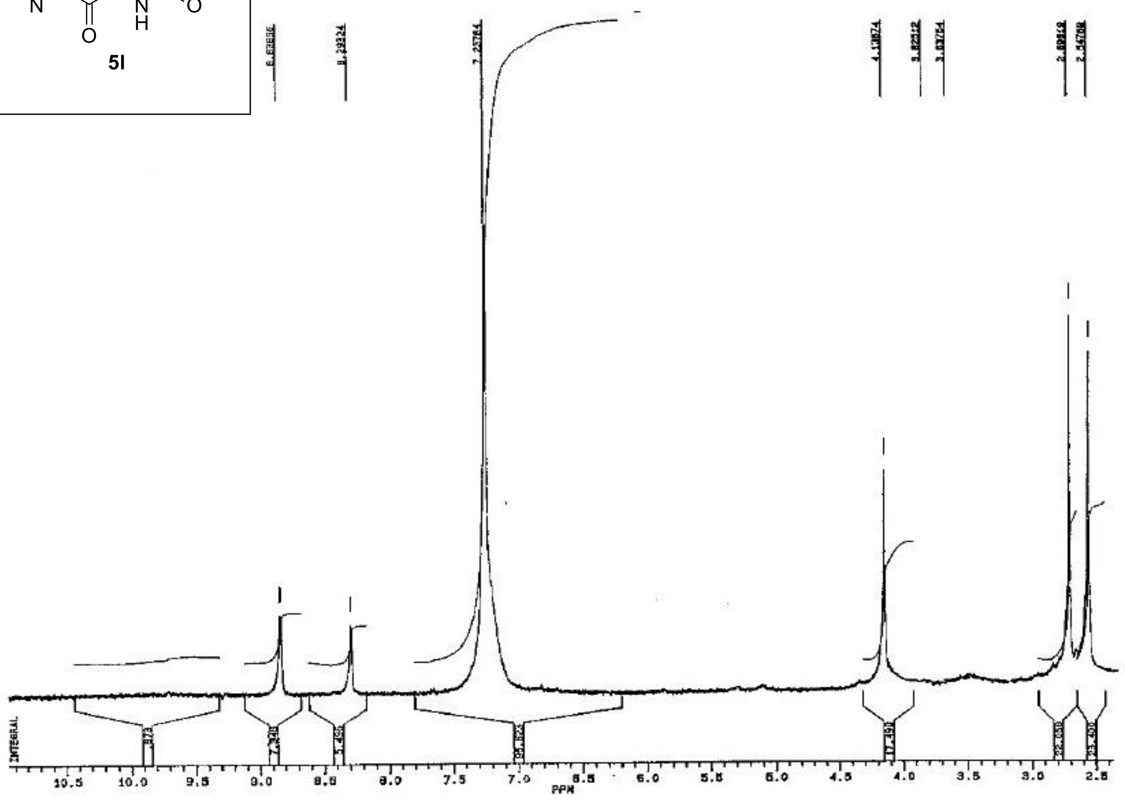
INTEGRAL
10.5
10.0
9.5
9.0
8.5
8.0
7.5
7.0
6.5
6.0
5.5
5.0
4.5
4.0
3.5
3.0
2.5
PPM

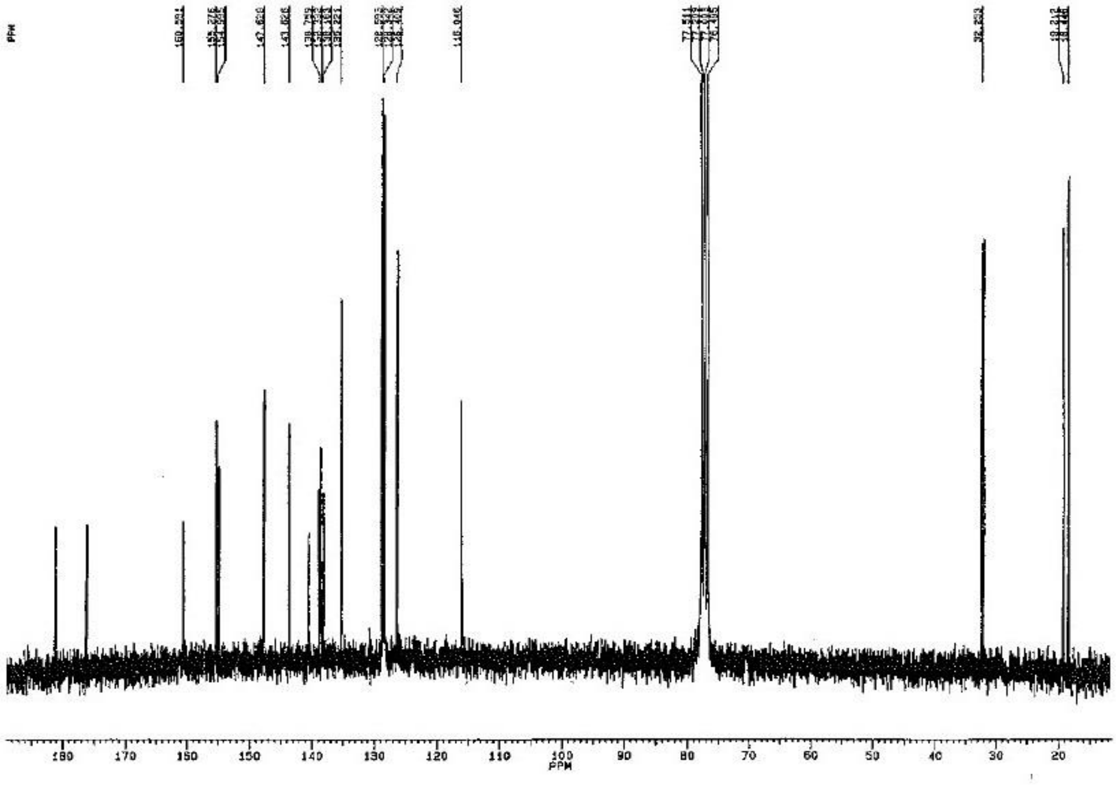
PPM
180
170
160
150
140
130
120
110
100
90
80
70
60
50
40
30
20
PPM

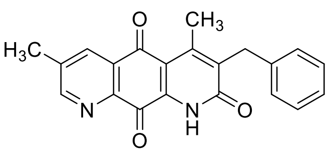
H3C
O
CH3
N
O
H
N
O

5m

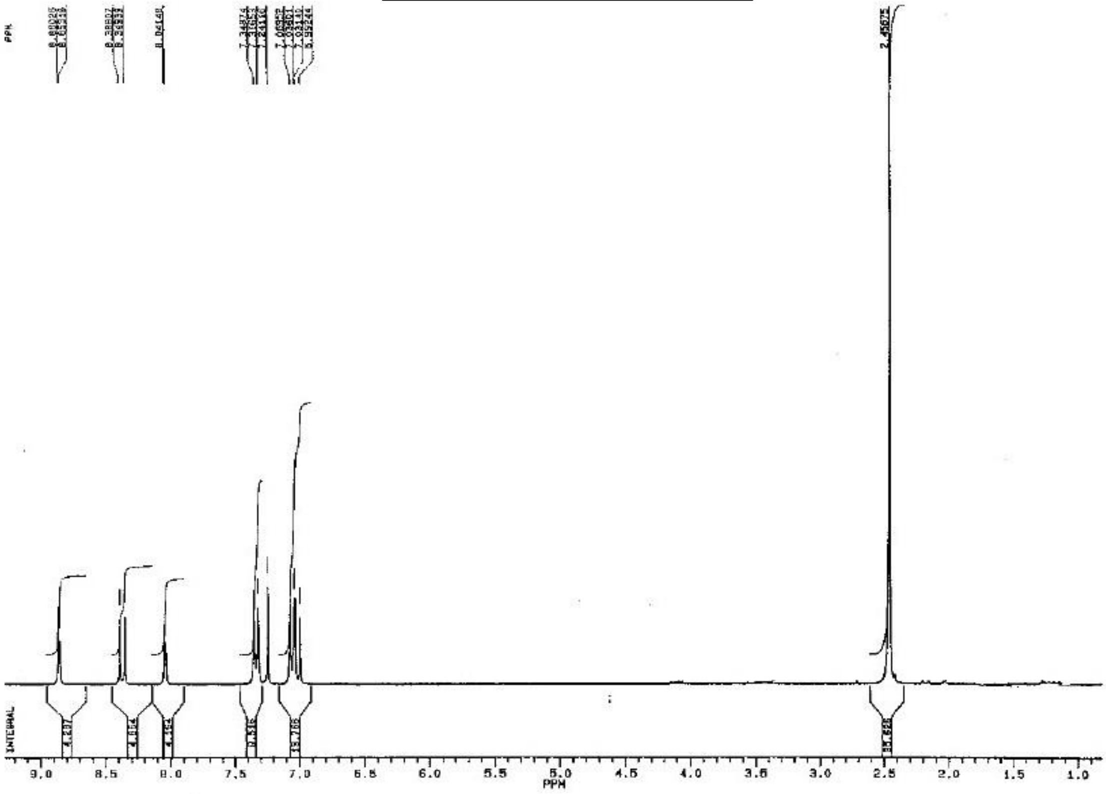
PPM
INTERNAL
2.45675
9.0
8.5
8.0
7.5
7.0
6.5
6.0
5.5
5.0
4.5
4.0
3.5
3.0
2.5
2.0
1.5
1.0
PPM

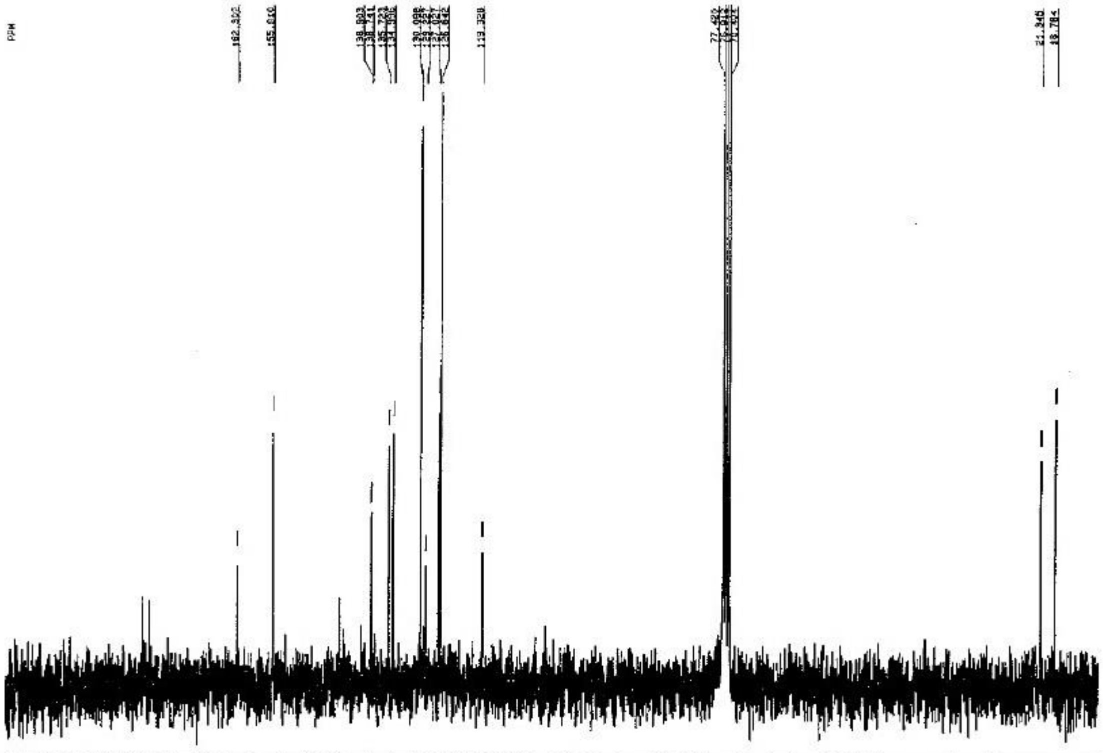
PPM
162.302
155.610
119.328
21.348
18.784
200
190
180
170
160
150
140
130
120
110
100
90
80
70
60
50
40
30
20
PPM

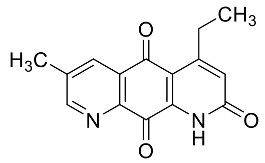

5n

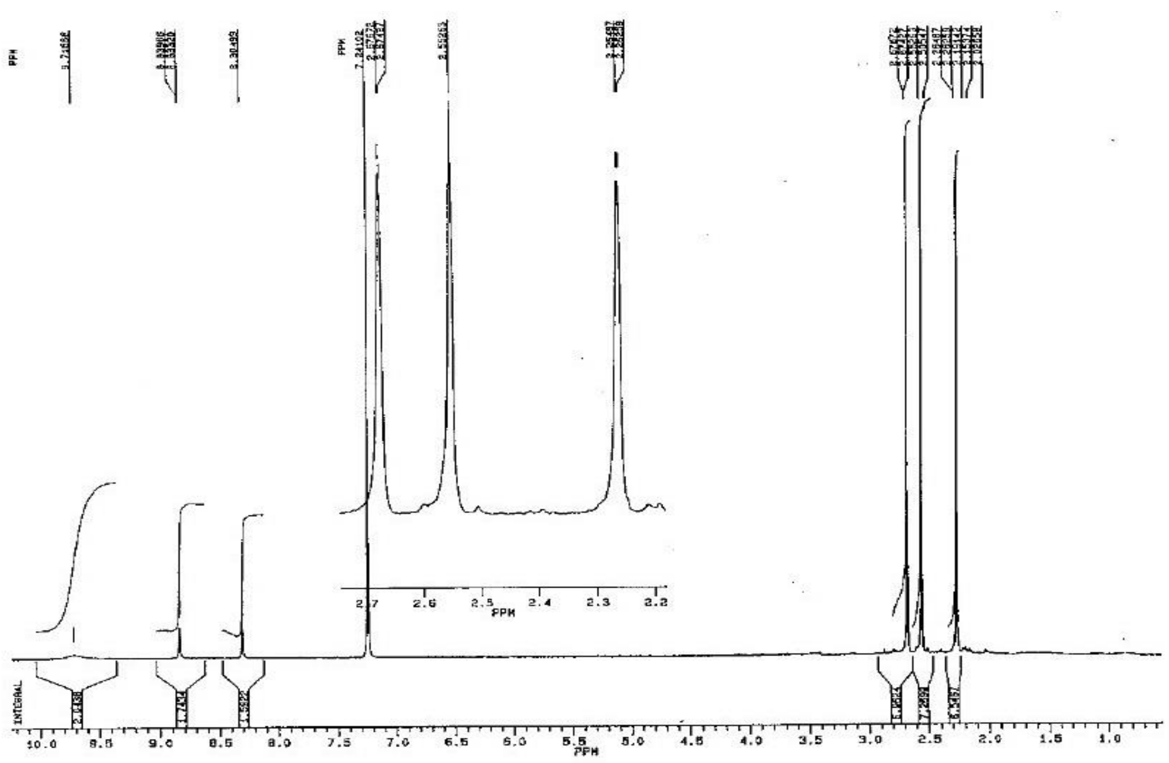

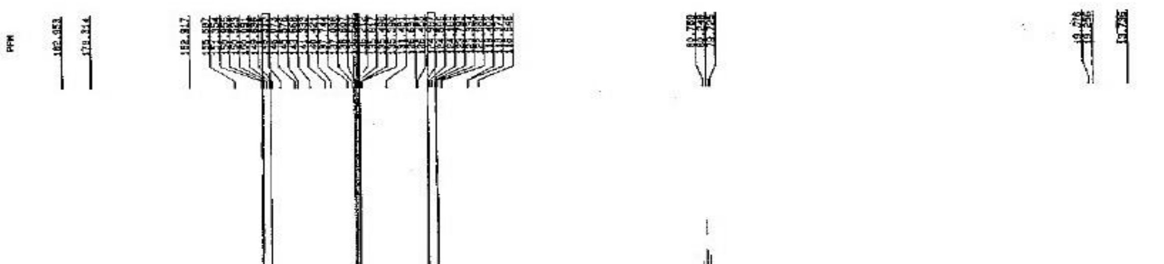

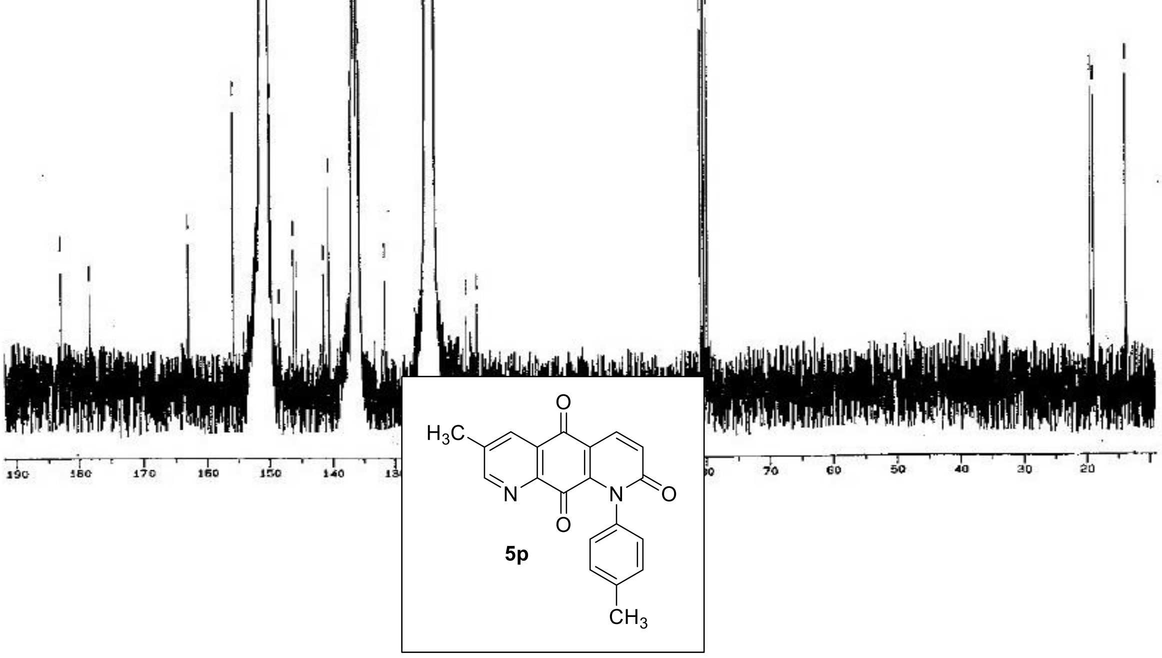

5p

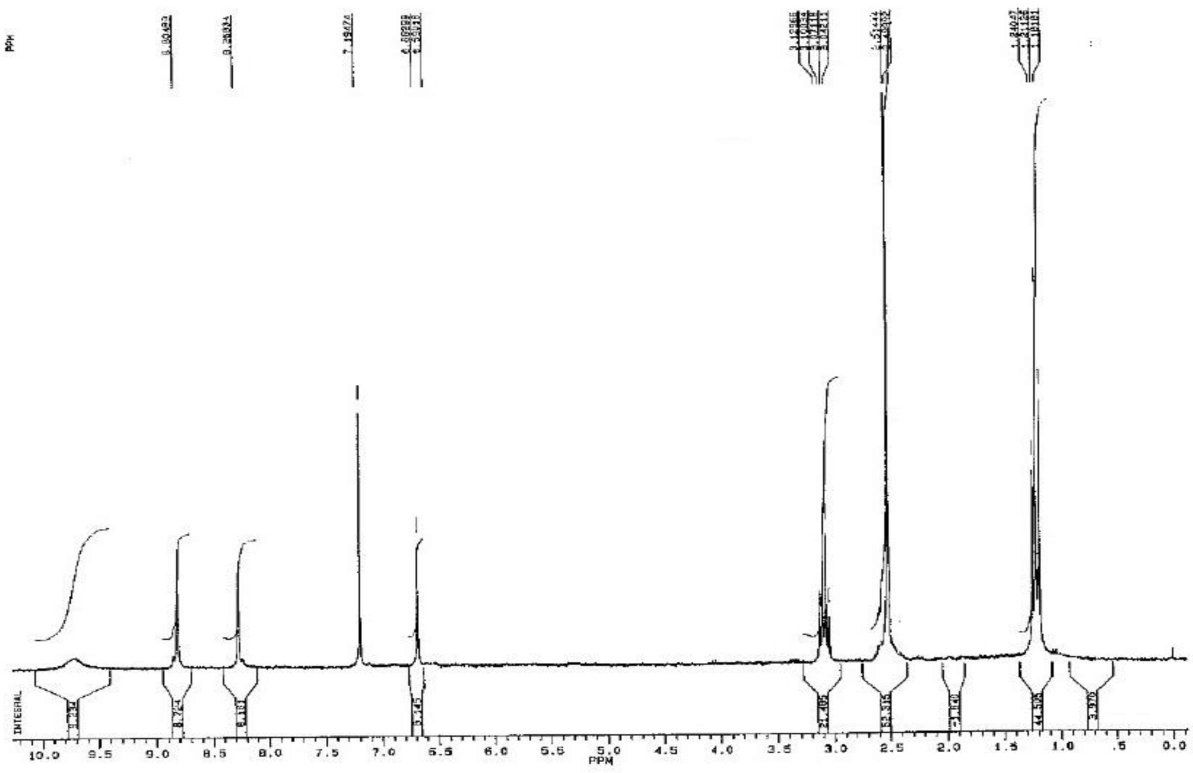

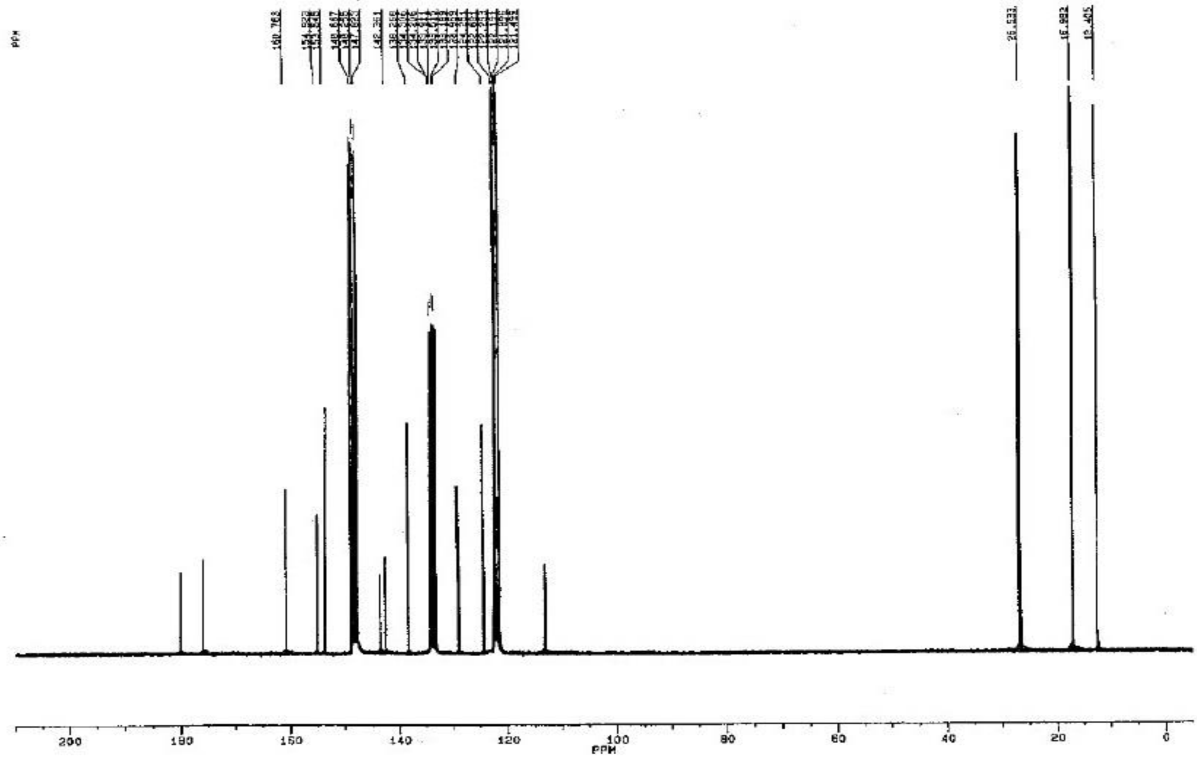

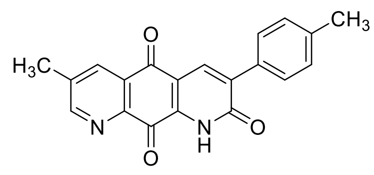

5q

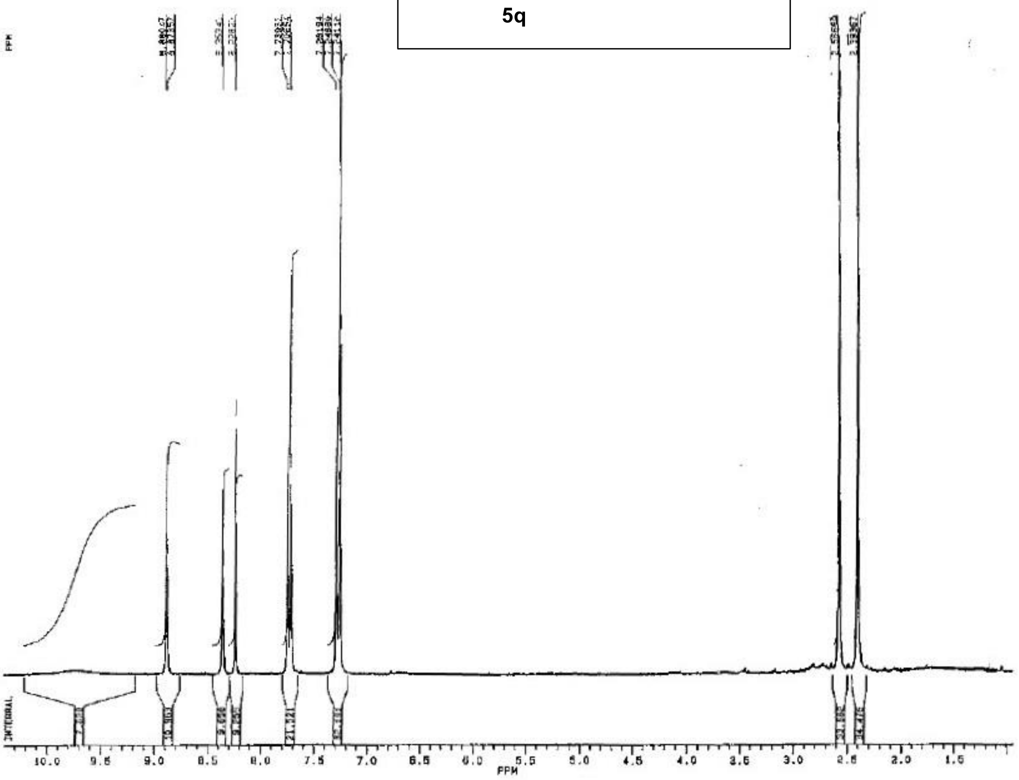

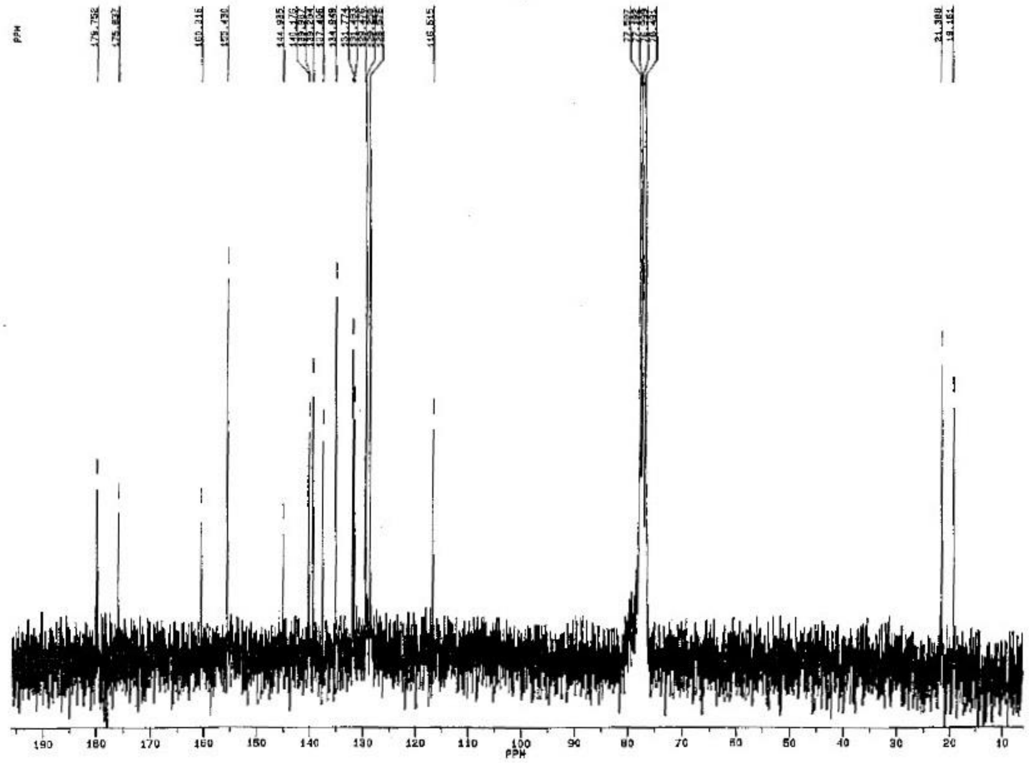

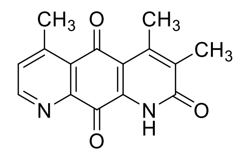

CH3
O
CH3
CH3
N
H
O
O
N

5r

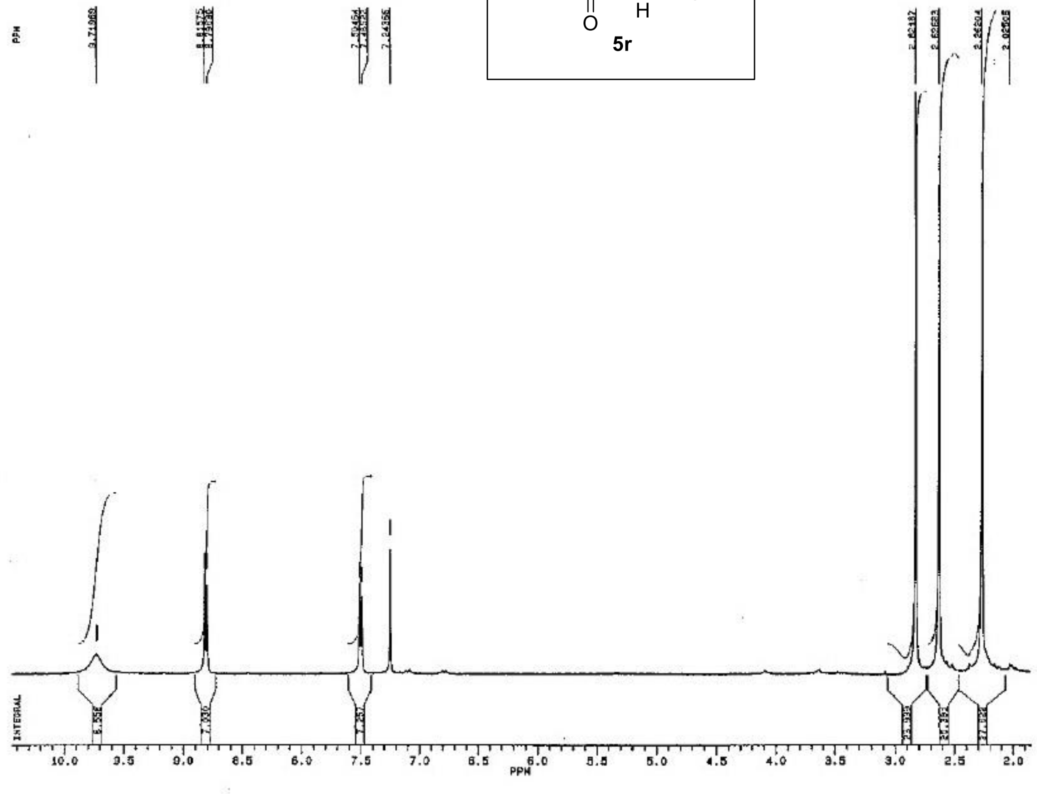

PPM
INTEGRAL
10.0
9.5
9.0
8.5
8.0
7.5
7.0
6.5
6.0
5.5
5.0
4.5
4.0
3.5
3.0
2.5
2.0
PPM

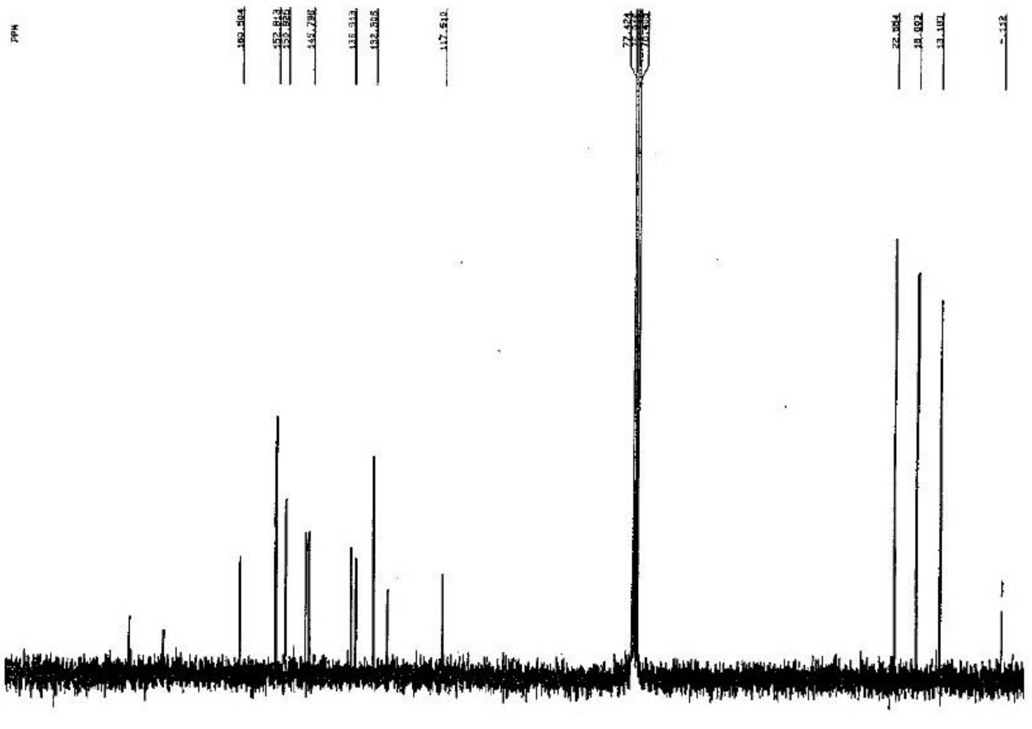

PPM
200
180
160
140
120
100
80
60
40
20
0
PPM

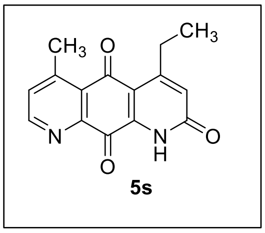

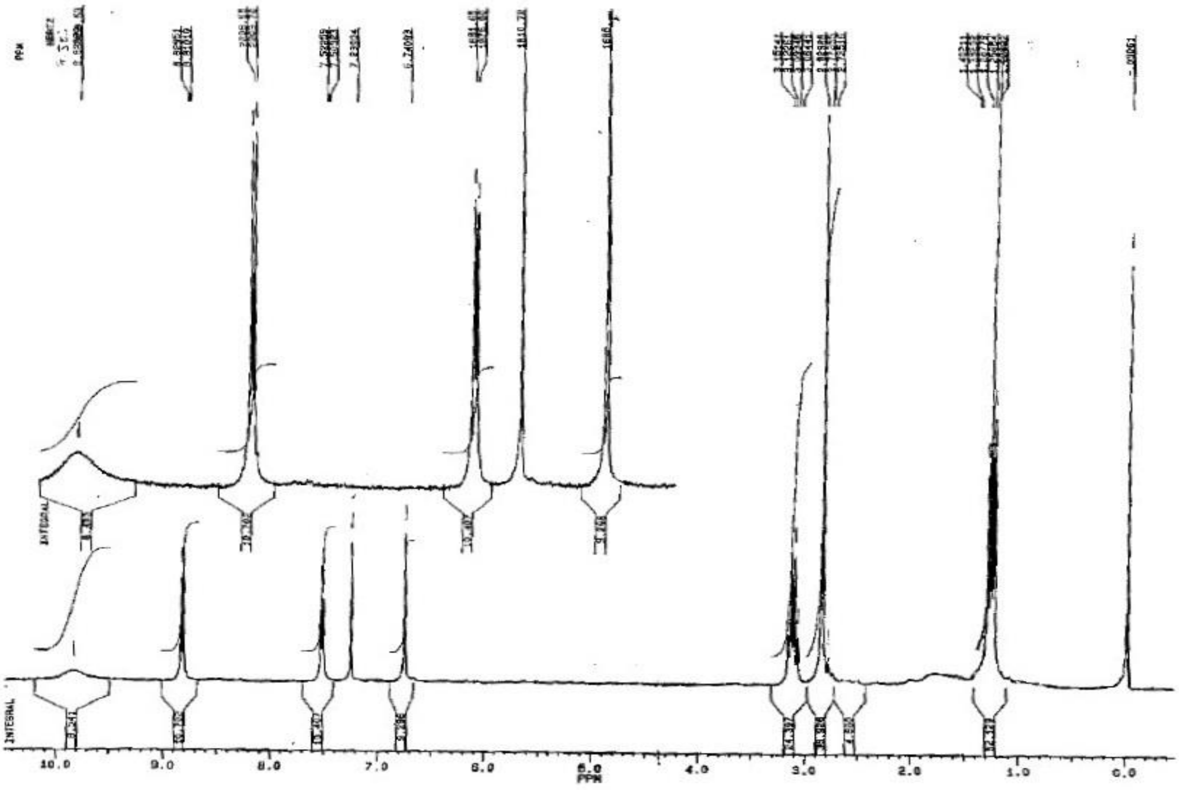

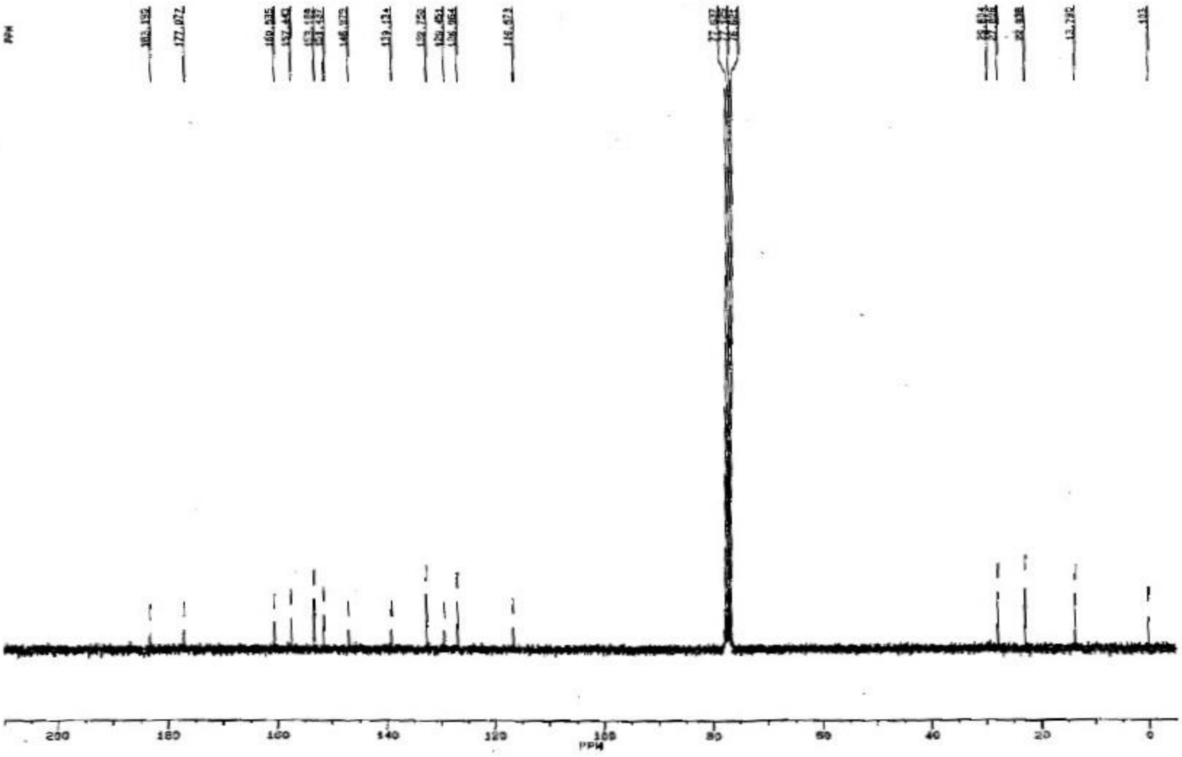

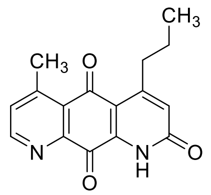

5t

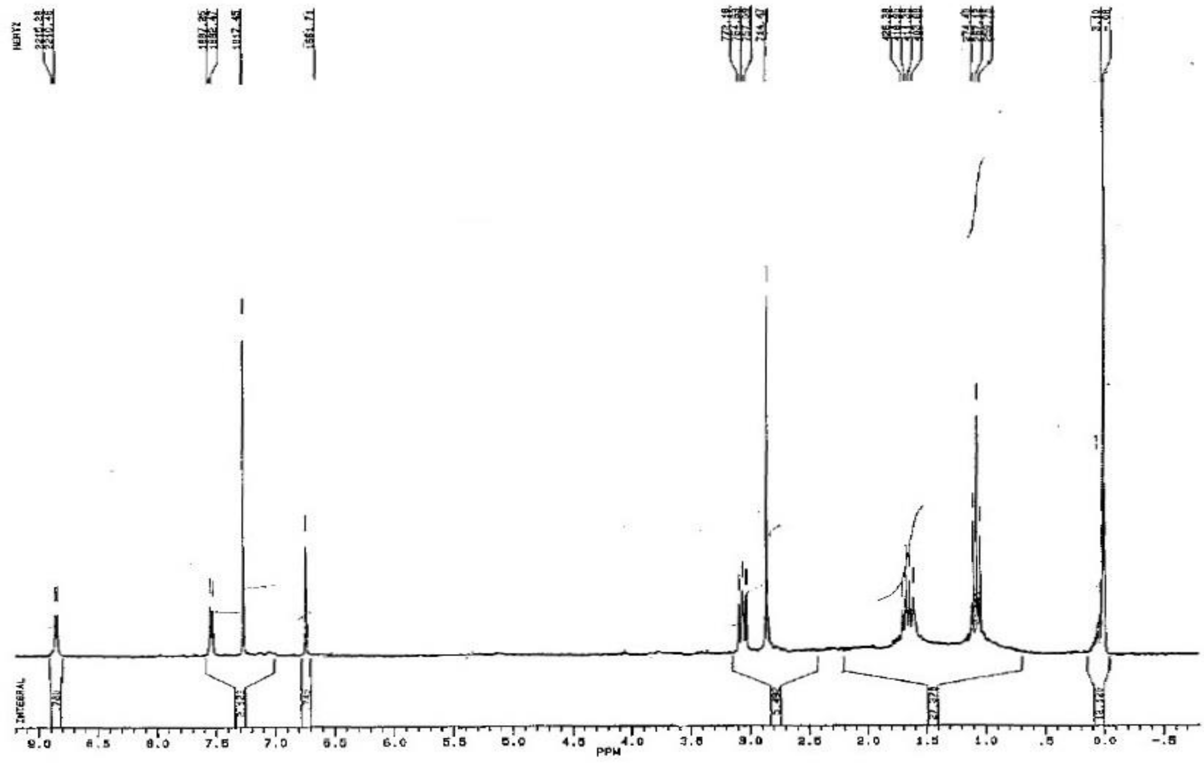

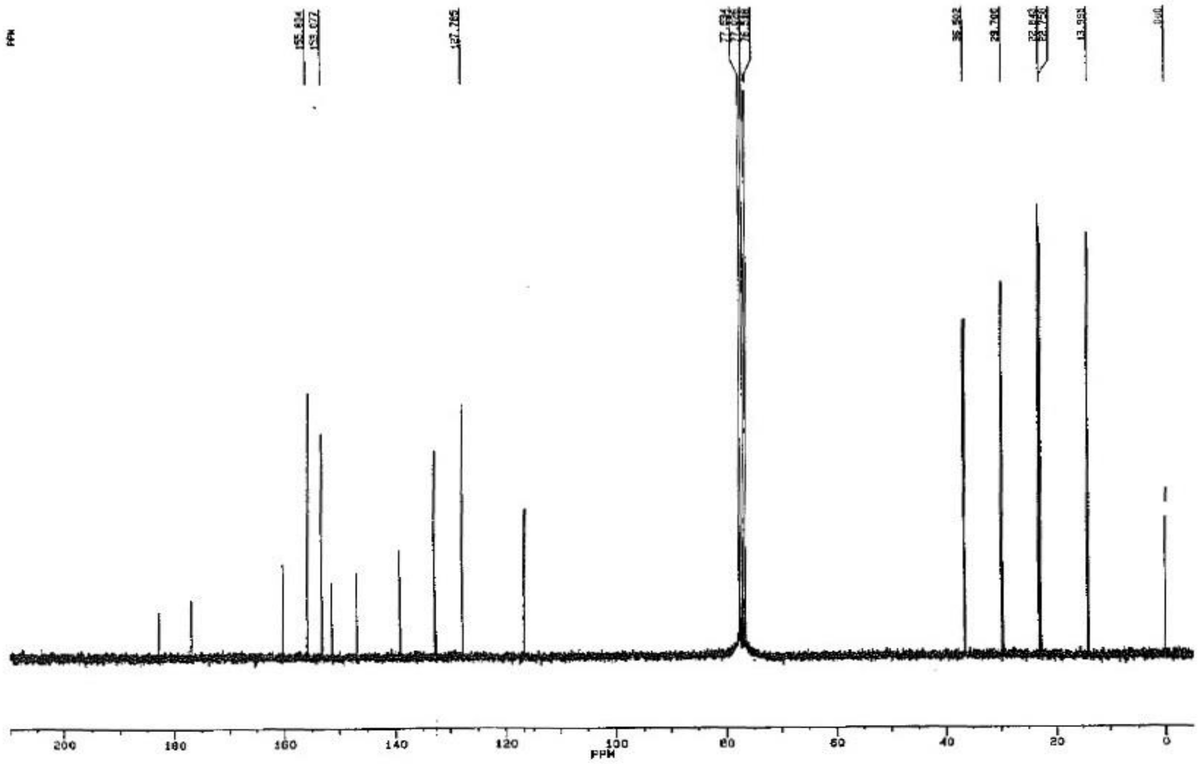

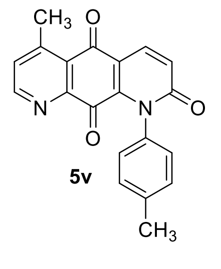
CH3
O
N
N
O
O
CH3

5v

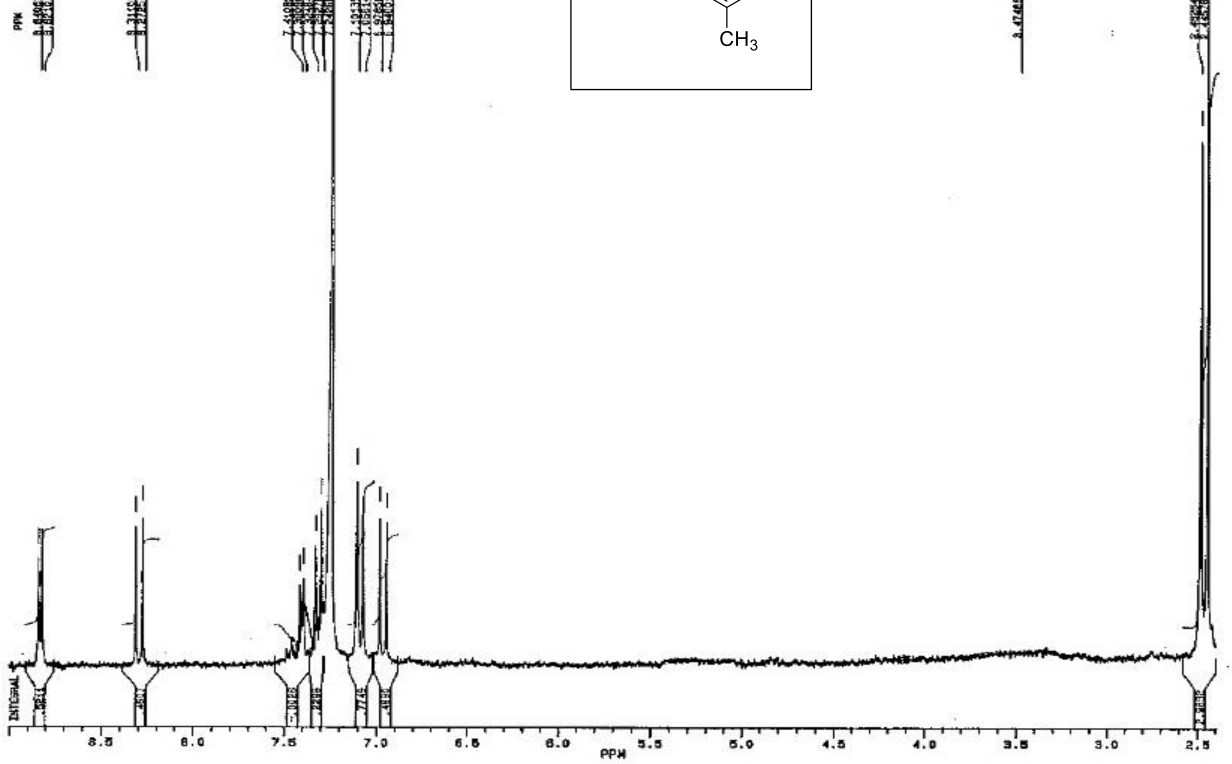
PPM
INTEGRAL
8.5
8.0
7.5
7.0
6.5
6.0
5.5
5.0
4.5
4.0
3.5
3.0
2.5
PPM

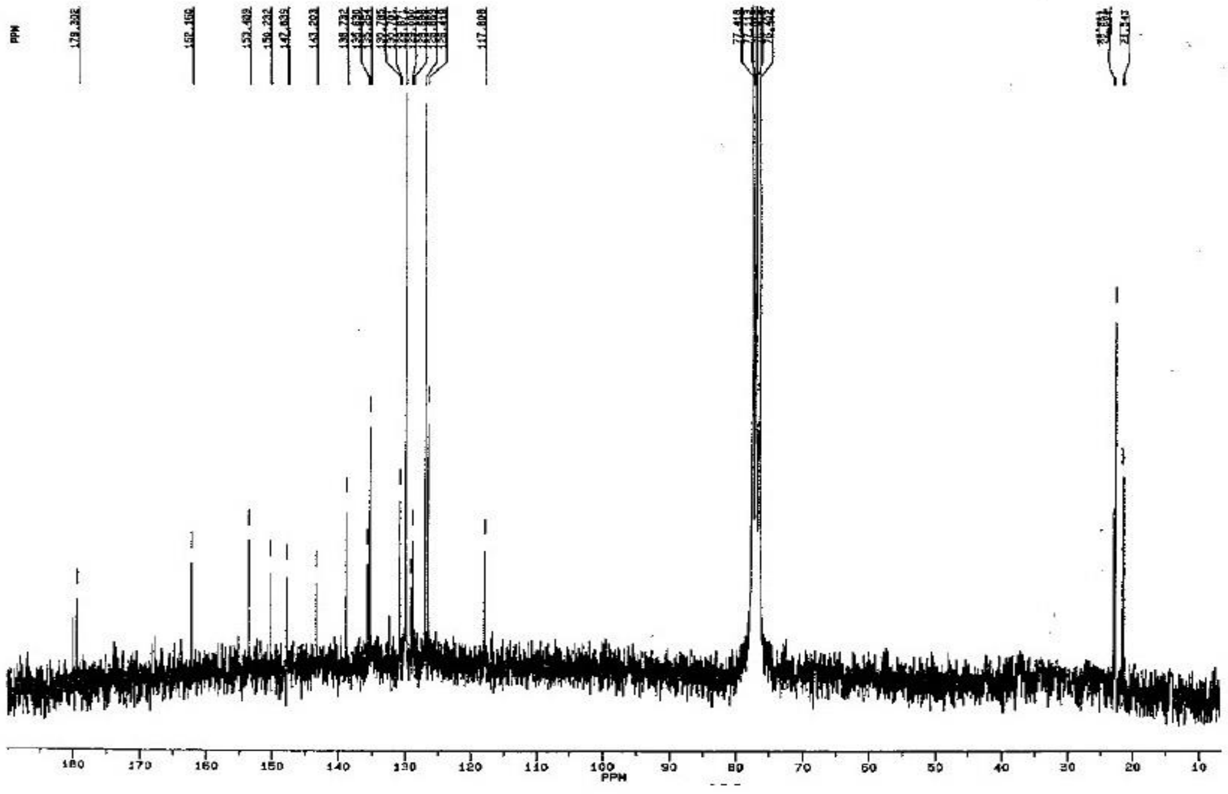
PPM
179.302
162.160
153.489
150.232
147.632
143.203
138.732
117.808
180
170
160
150
140
130
120
110
100
90
80
70
60
50
40
30
20
10
PPM

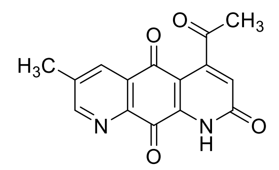

5ah

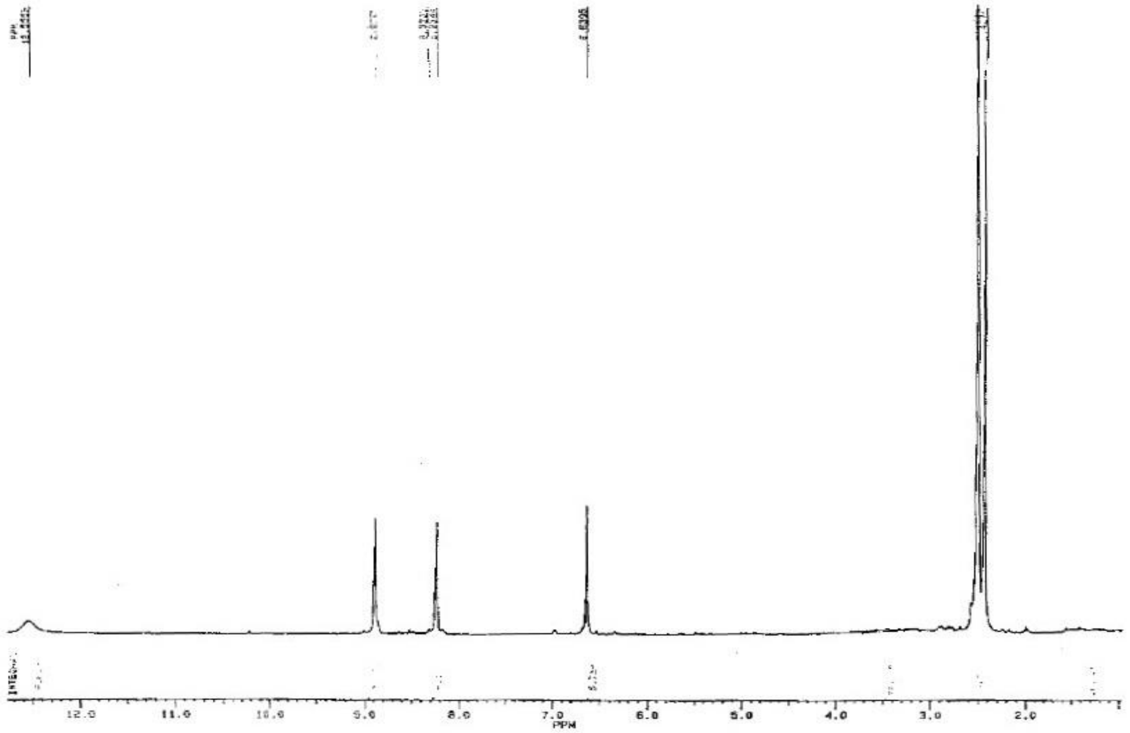

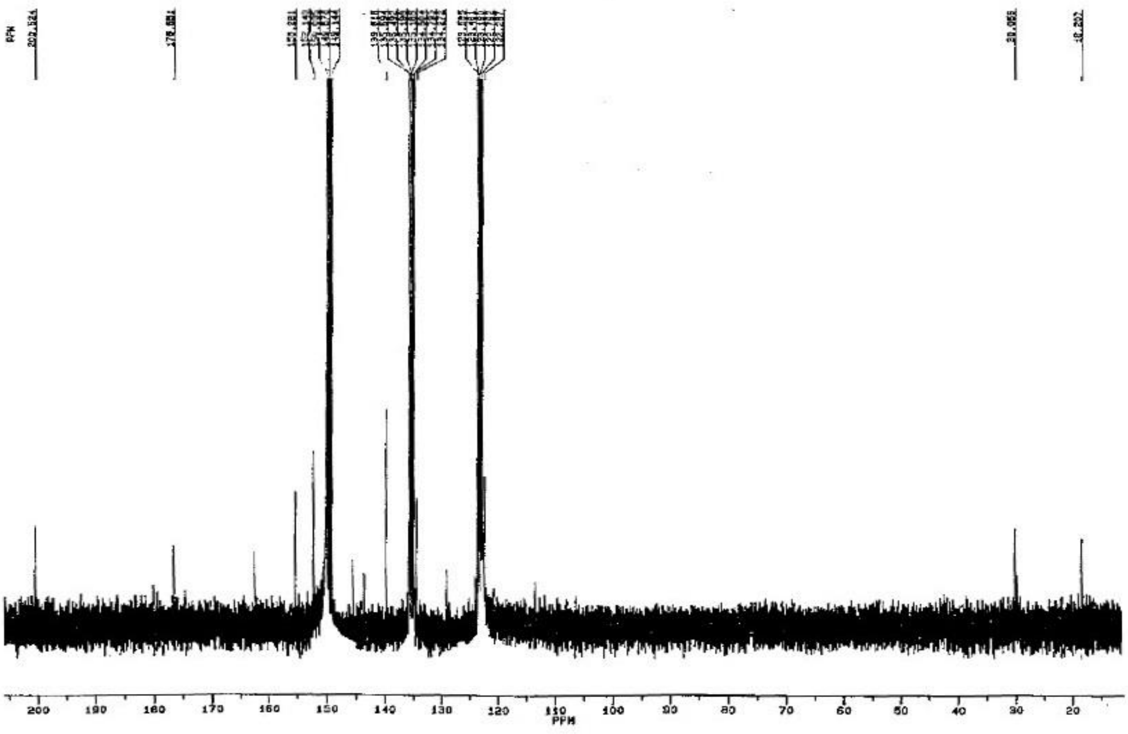

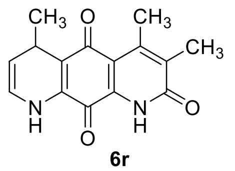

6r

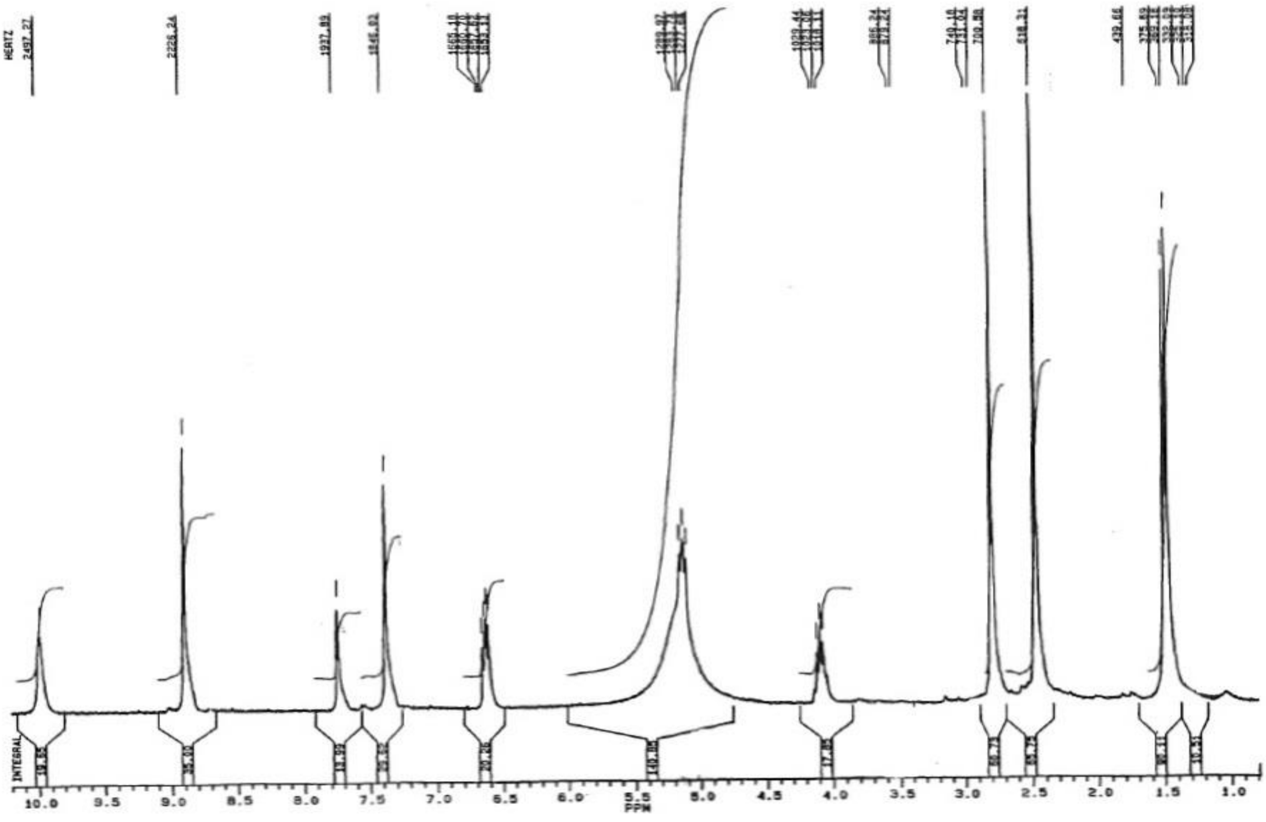

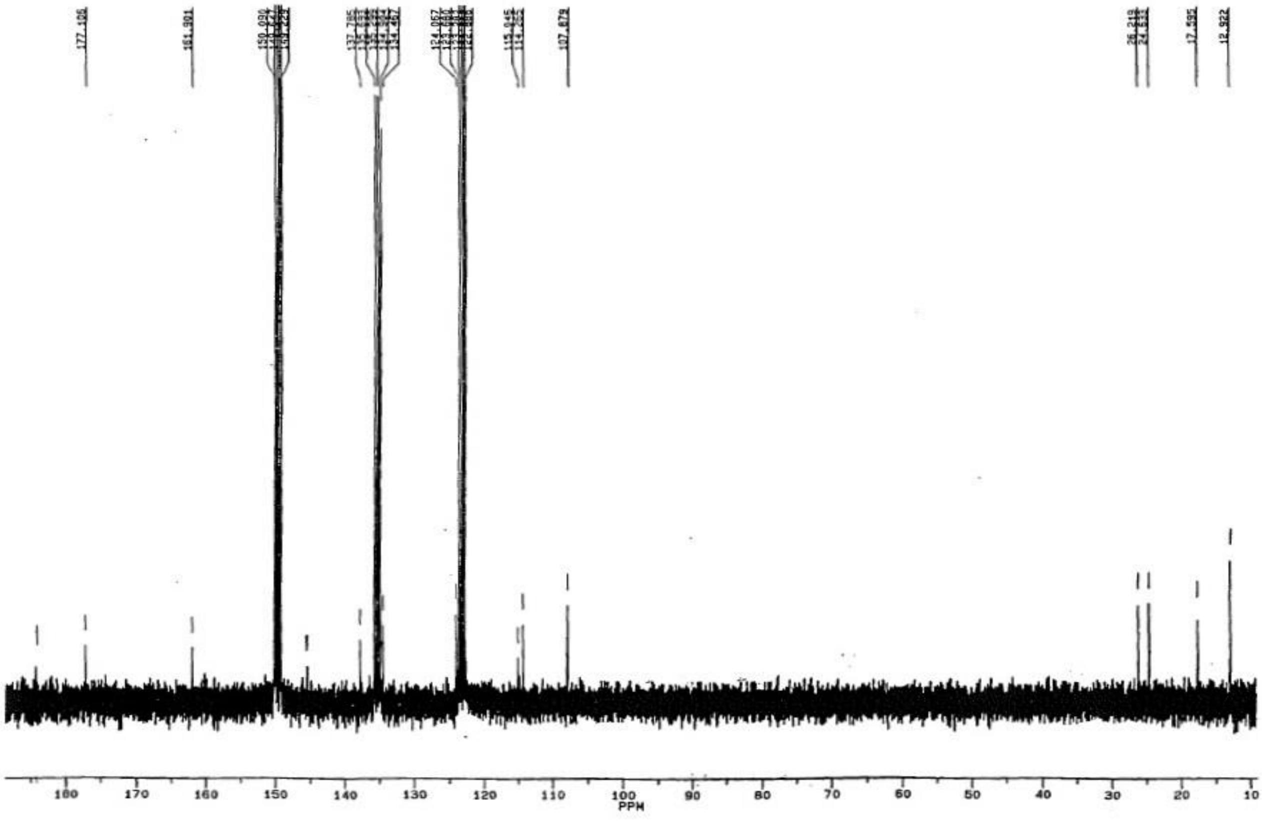

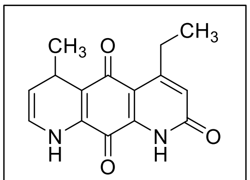

6s

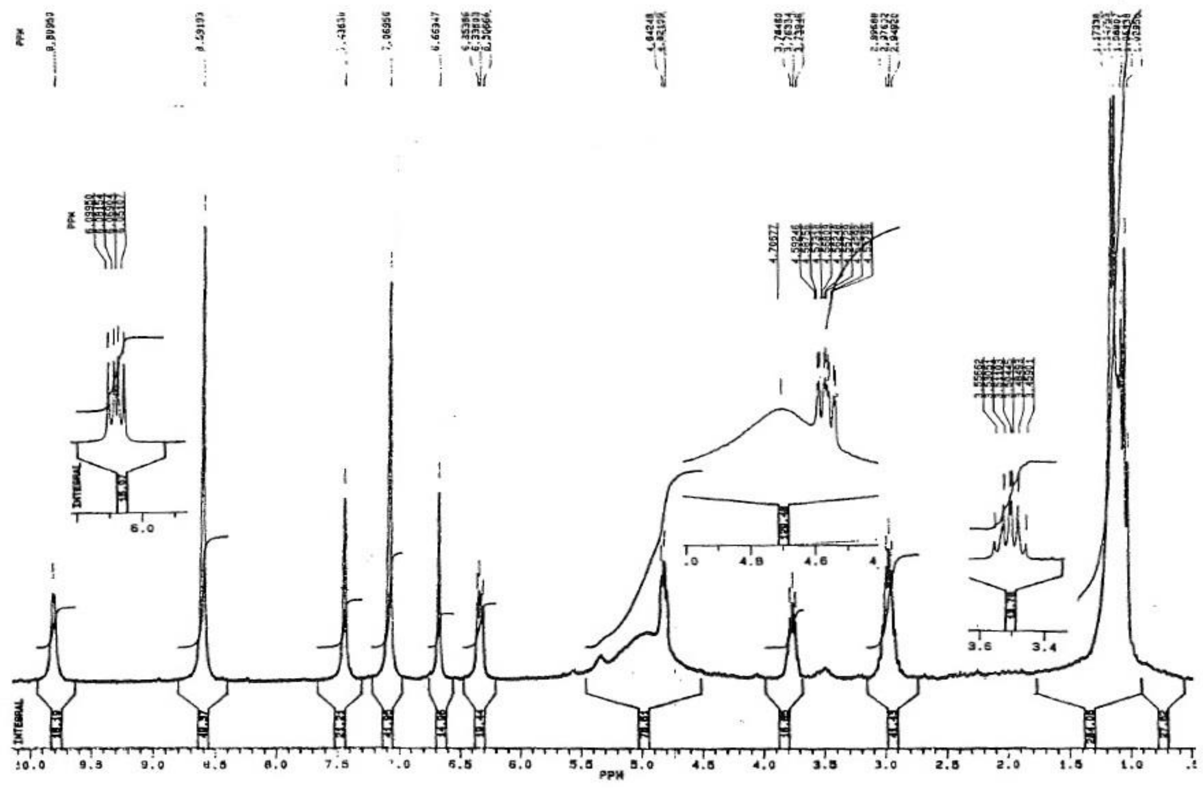

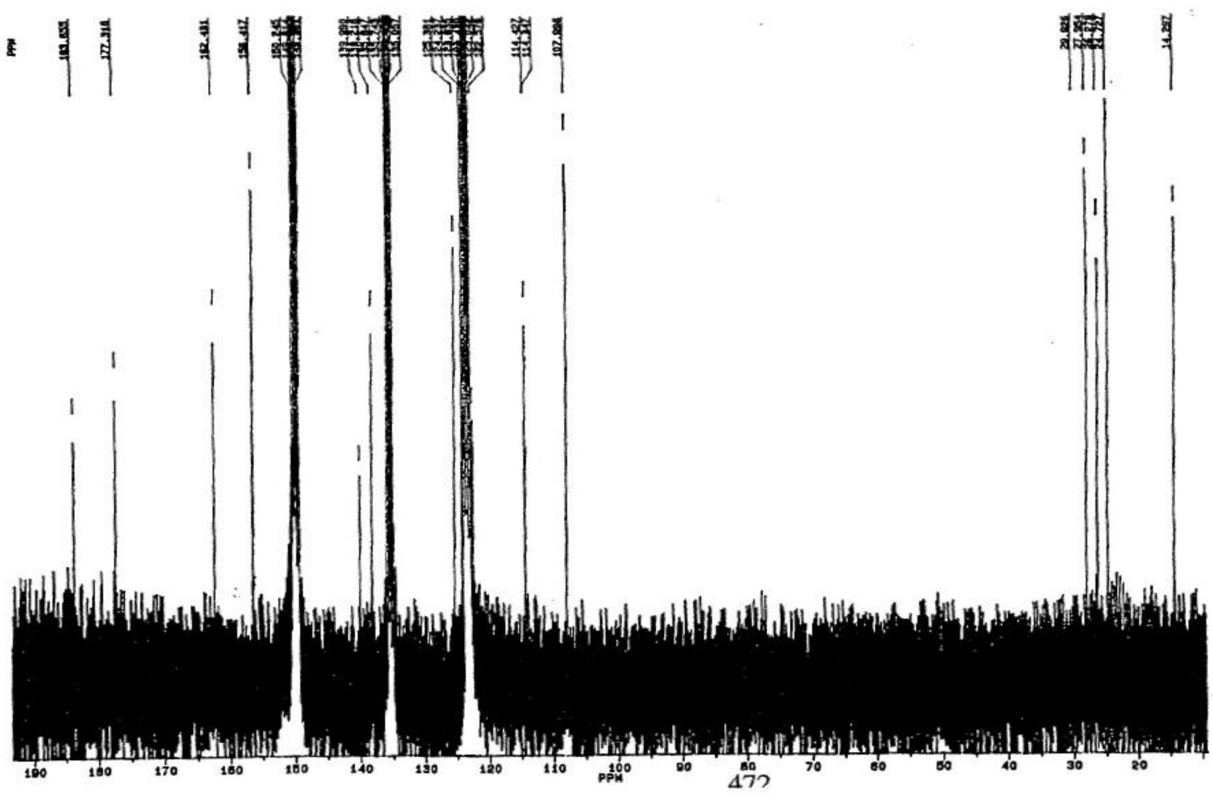

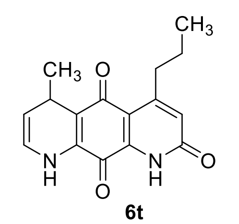

6t

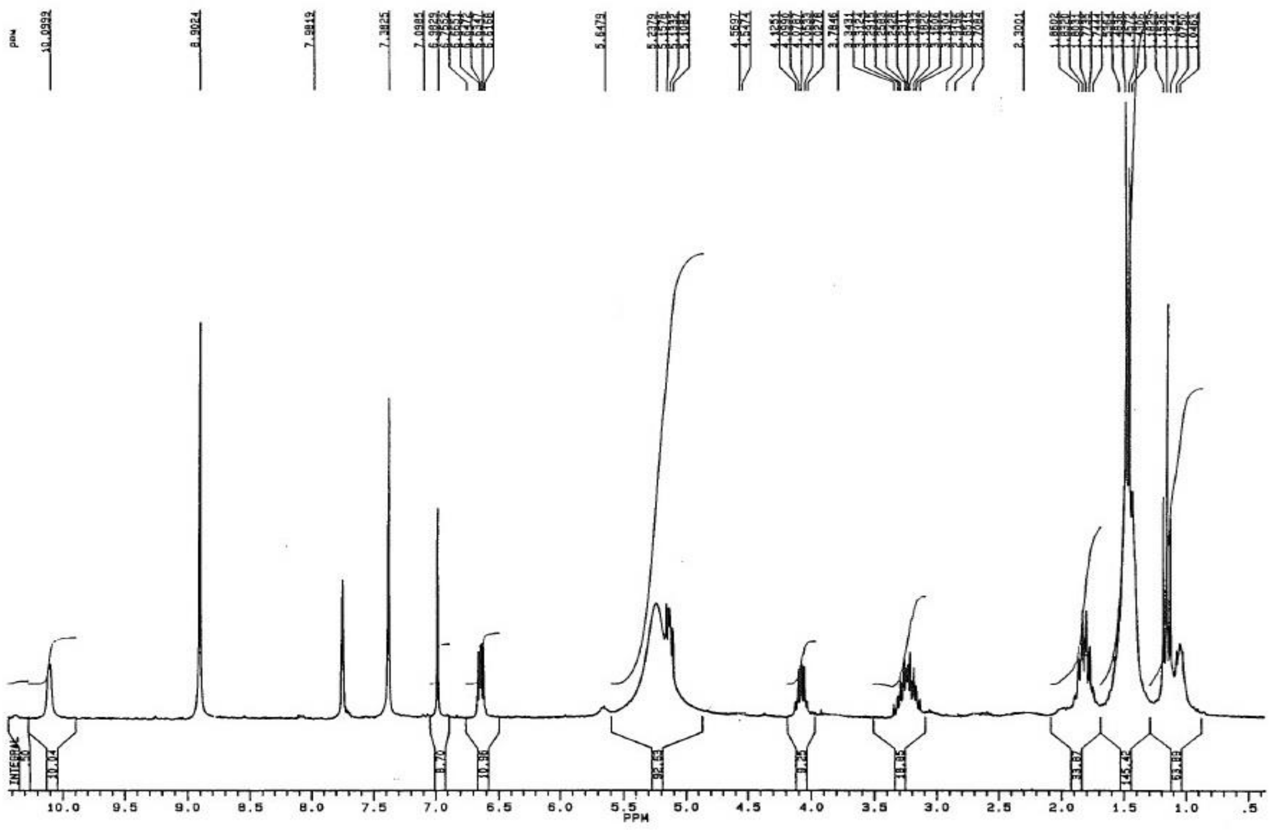

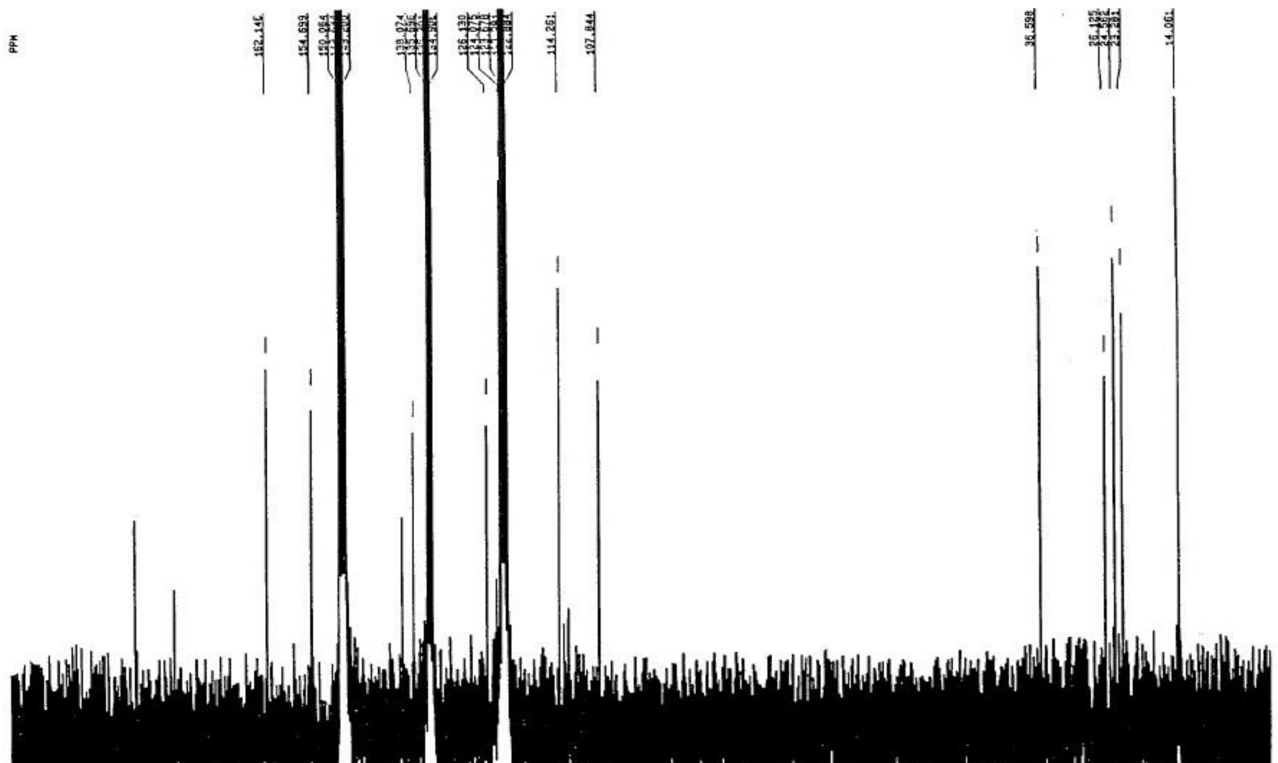

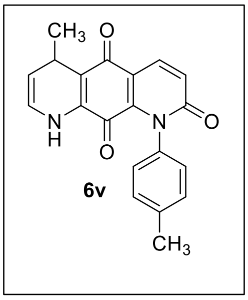
6v

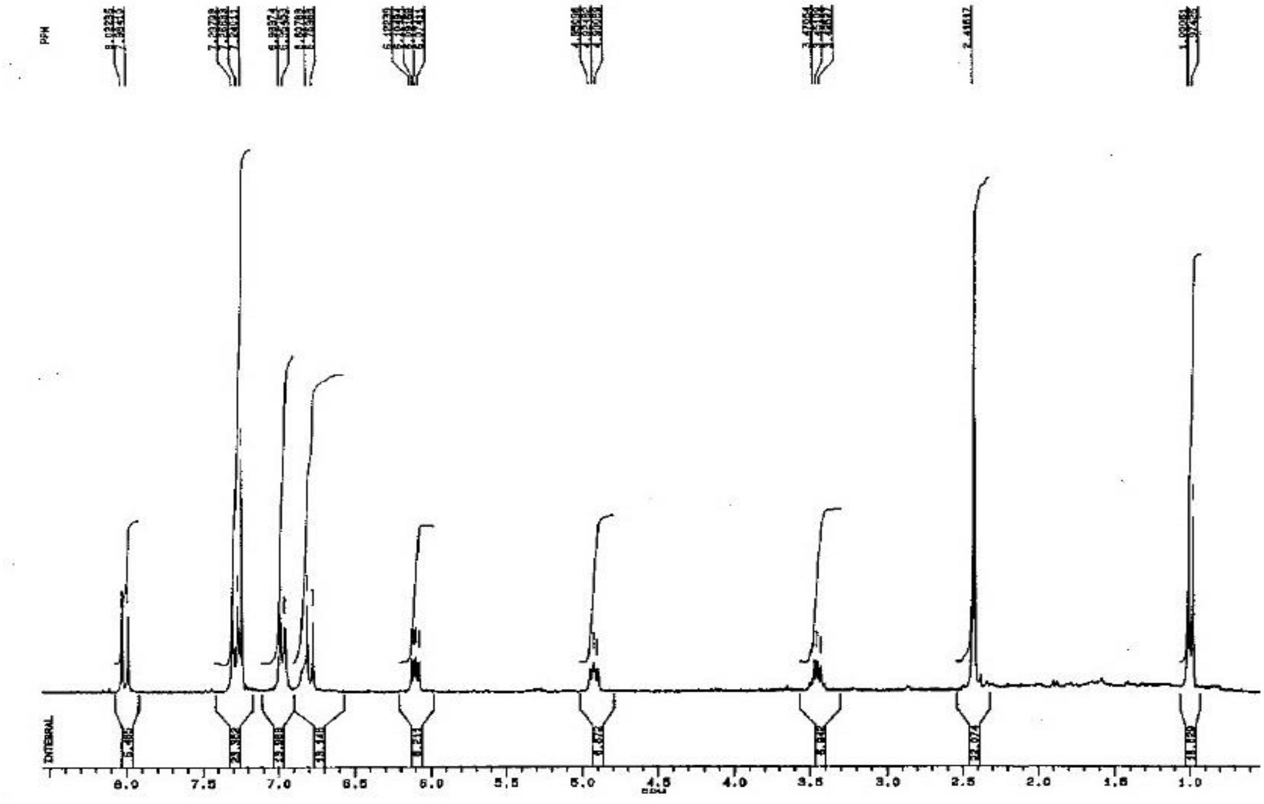

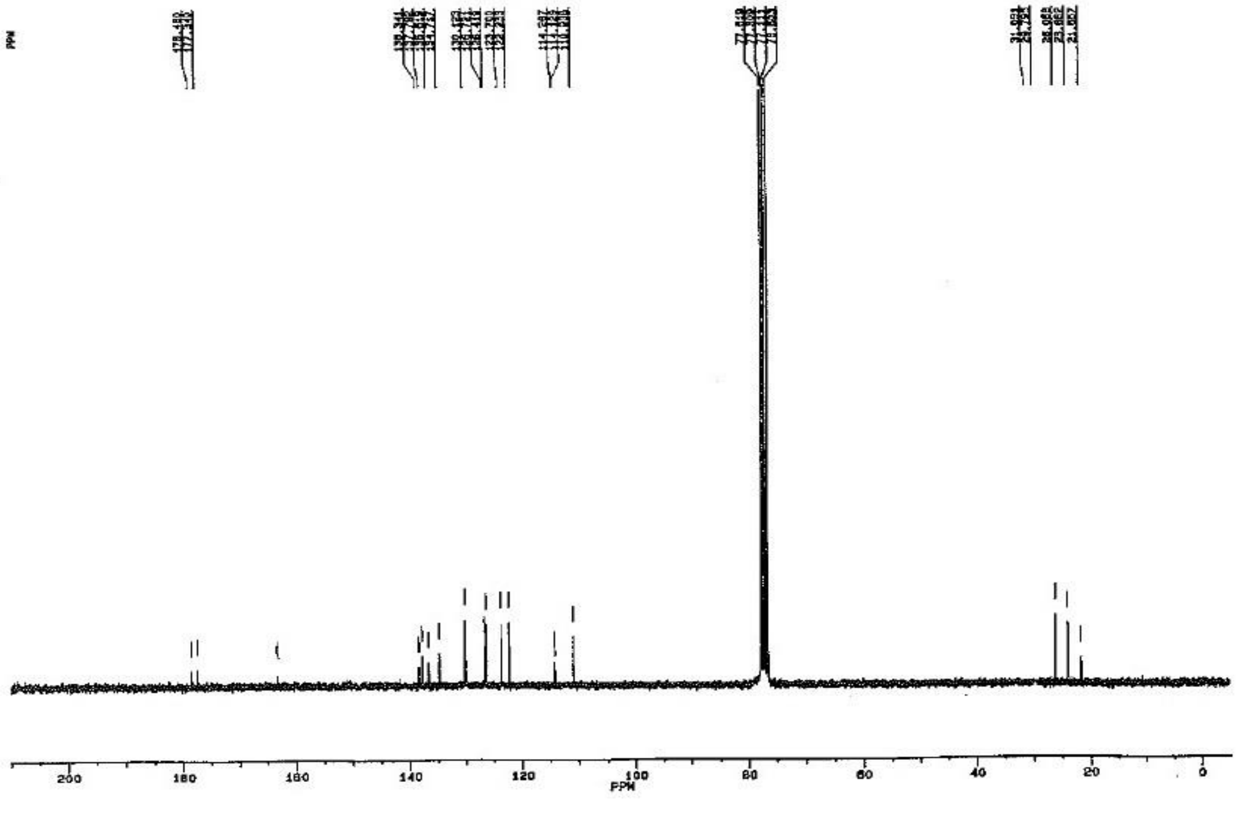

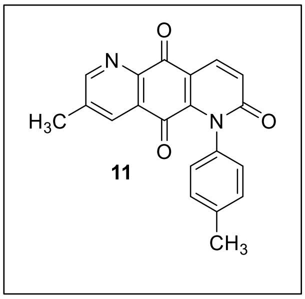
N
O
O
O
H3C
N
11
CH3

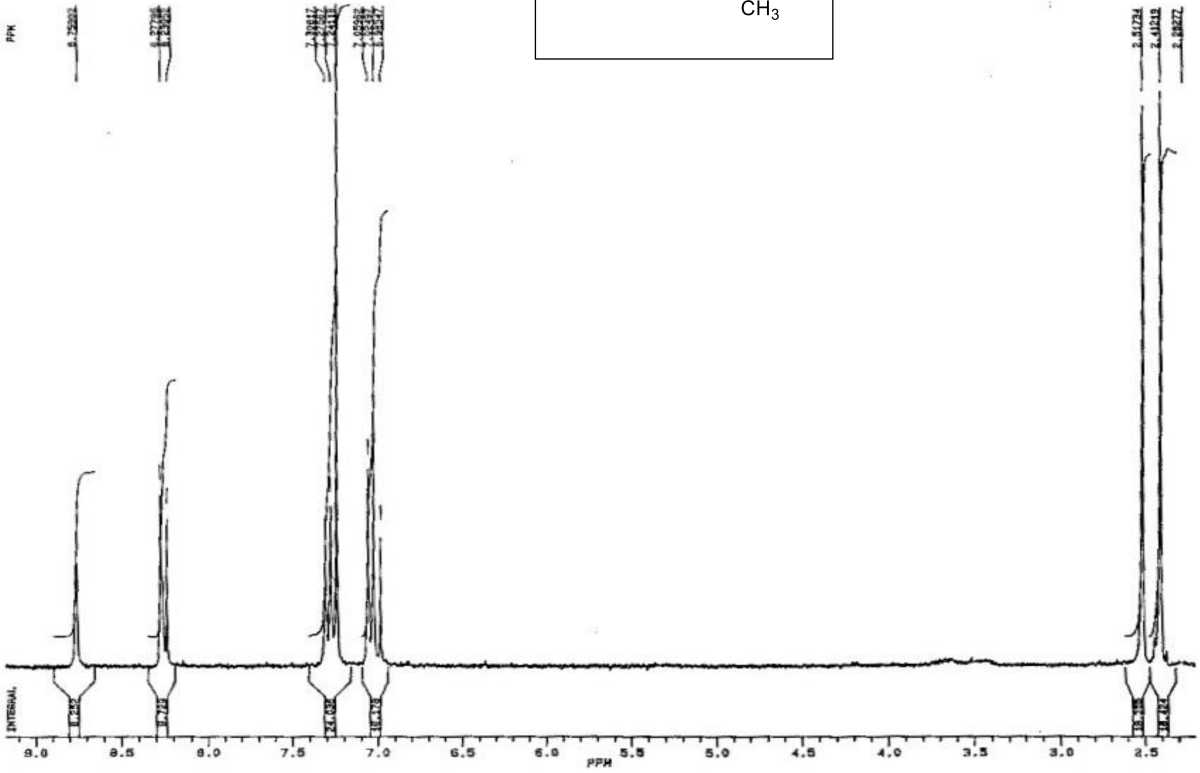
9.0
8.5
8.0
7.5
7.0
6.5
6.0
5.5
5.0
4.5
4.0
3.5
3.0
2.5
PPM

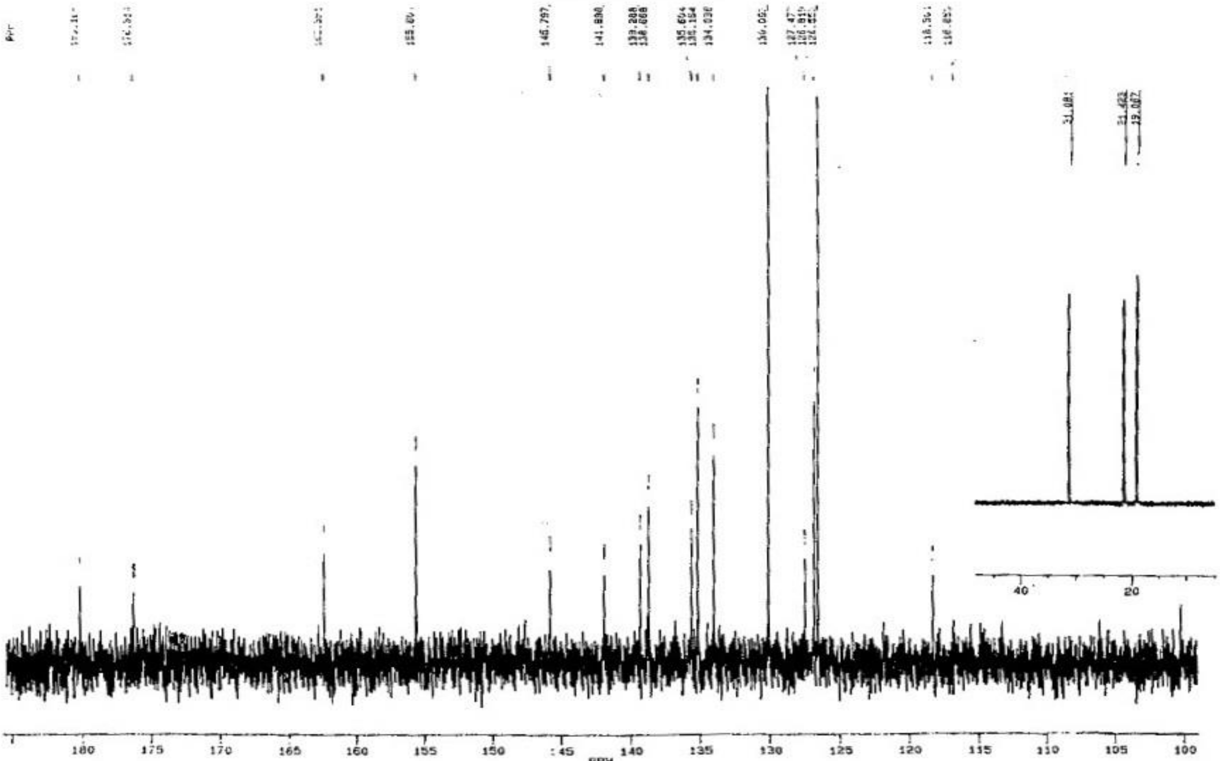
180
175
170
165
160
155
150
145
140
135
130
125
120
115
110
105
100
PPM
40
20

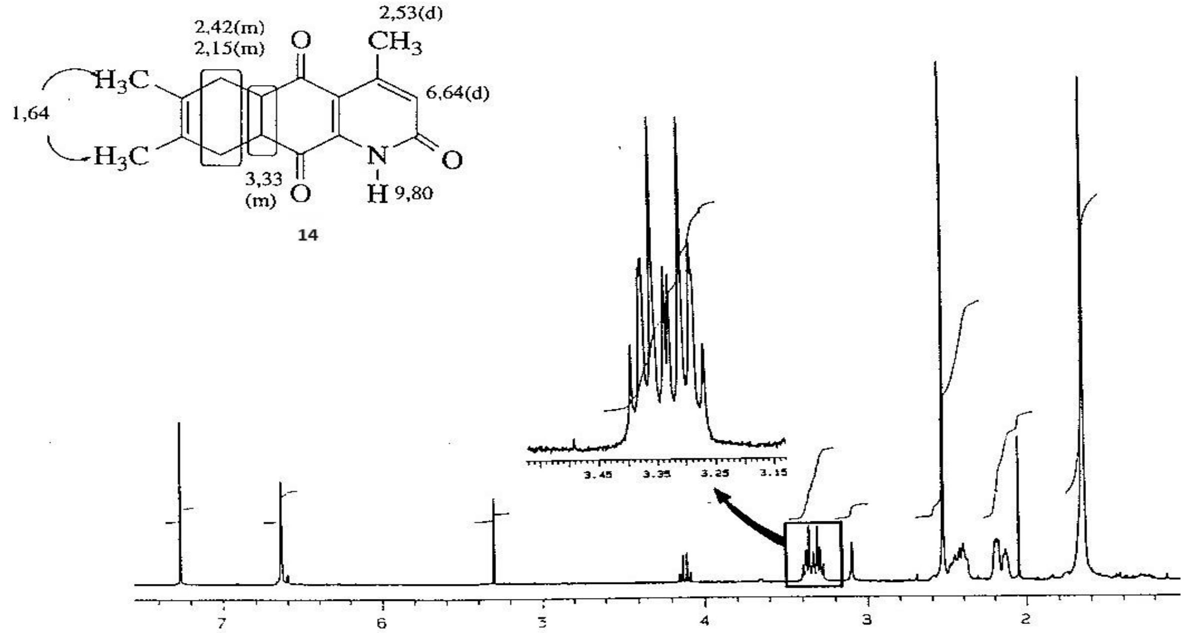
2,53(d)
2,42(m)
2,15(m)
CH3
H3C
H3C
6,64(d)
1,64
3,33
(m)
9,80
3.45 3.35 3.25 3.15
7 6 5 4 3 2

14

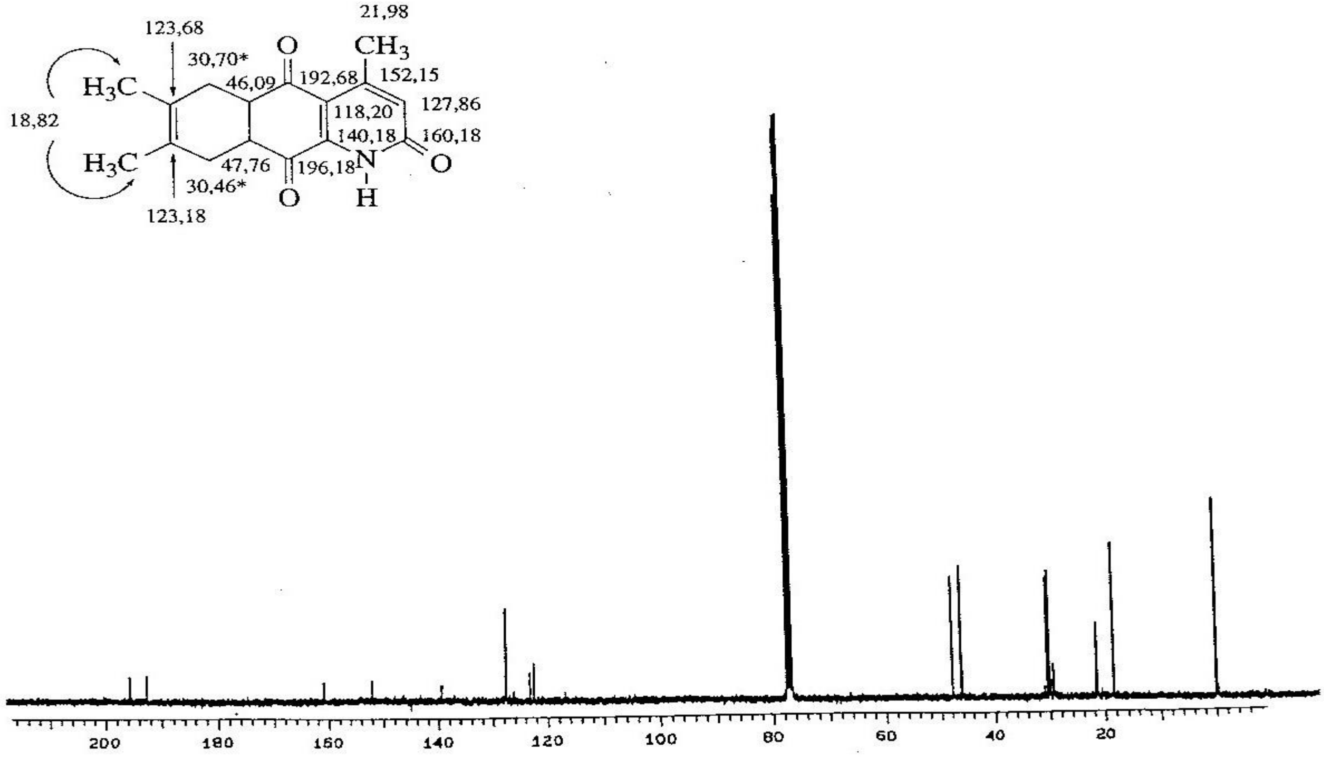
21,98
123,68
30,70*
46,09
192,68
152,15
127,86
118,20
140,18
160,18
18,82
47,76
196,18
30,46*
123,18
200 180 160 140 120 100 80 60 40 20

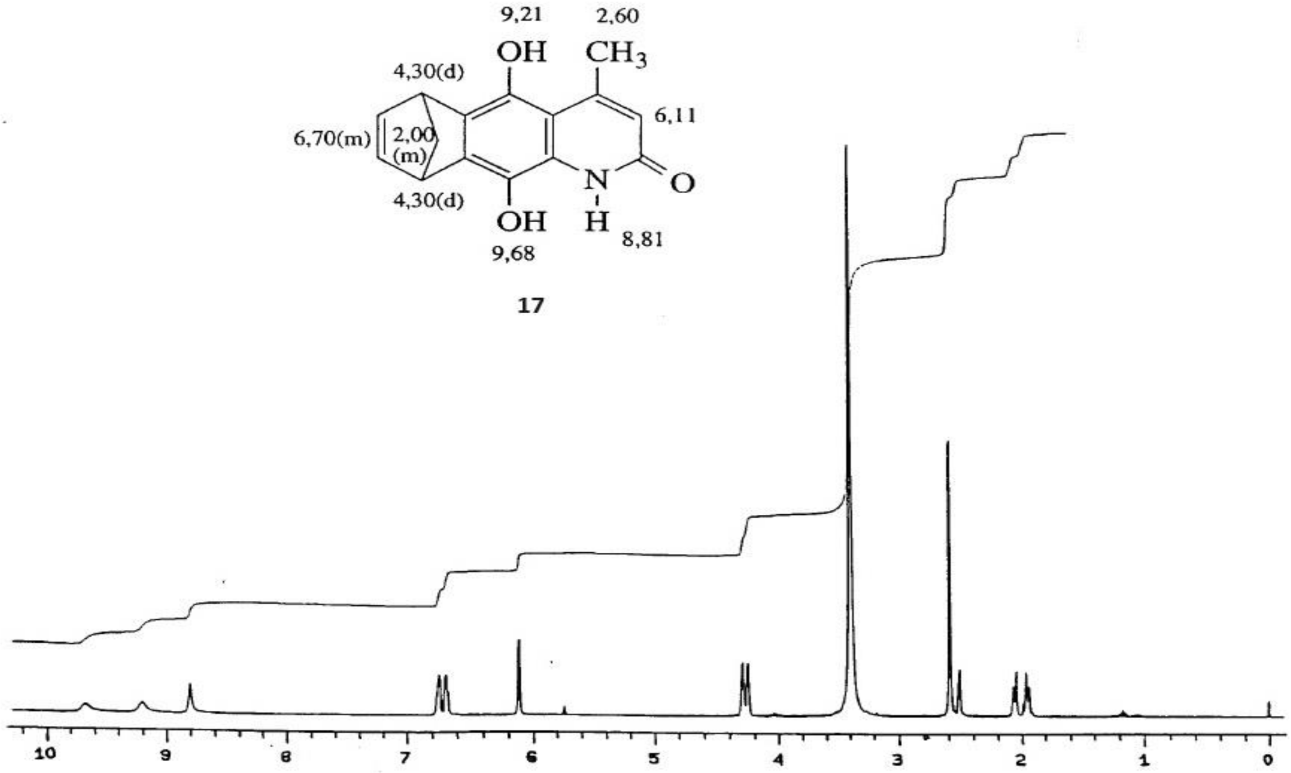

9,21
2,60
OH
CH3
4,30(d)
6,11
6,70(m)
2,00
(m)
N
O
4,30(d)
OH
H
9,68
8,81
17
10 9 8 7 6 5 4 3 2 1 0

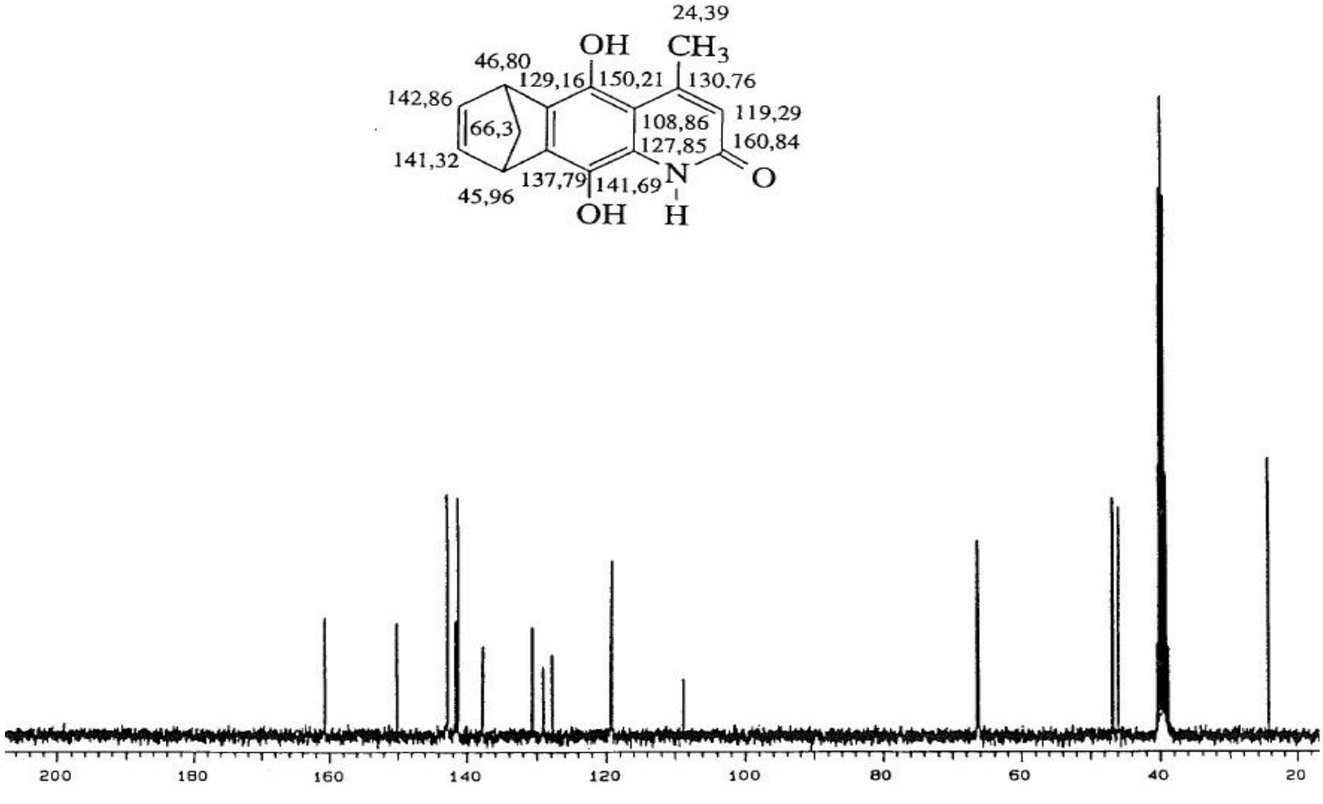

24,39
OH
CH3
46,80
129,16
150,21
130.76
142,86
108,86
119,29
66,3
127,85
160,84
141,32
N
O
137,79
141,69
45,96
OH
H
200 180 160 140 120 100 80 60 40 20

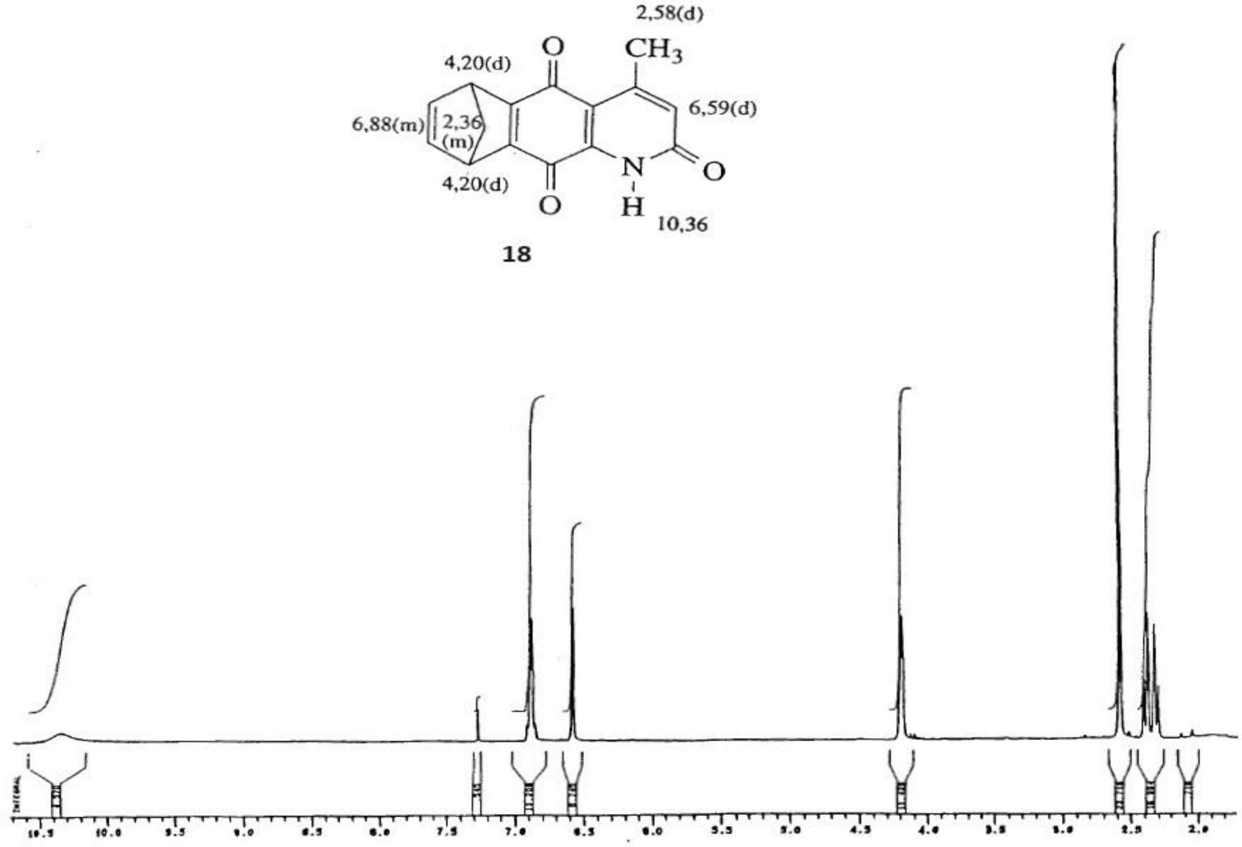
2,58(d)
CH3
4,20(d)
6,59(d)
6,88(m)
2,36
(m)
4,20(d)
10,36

18

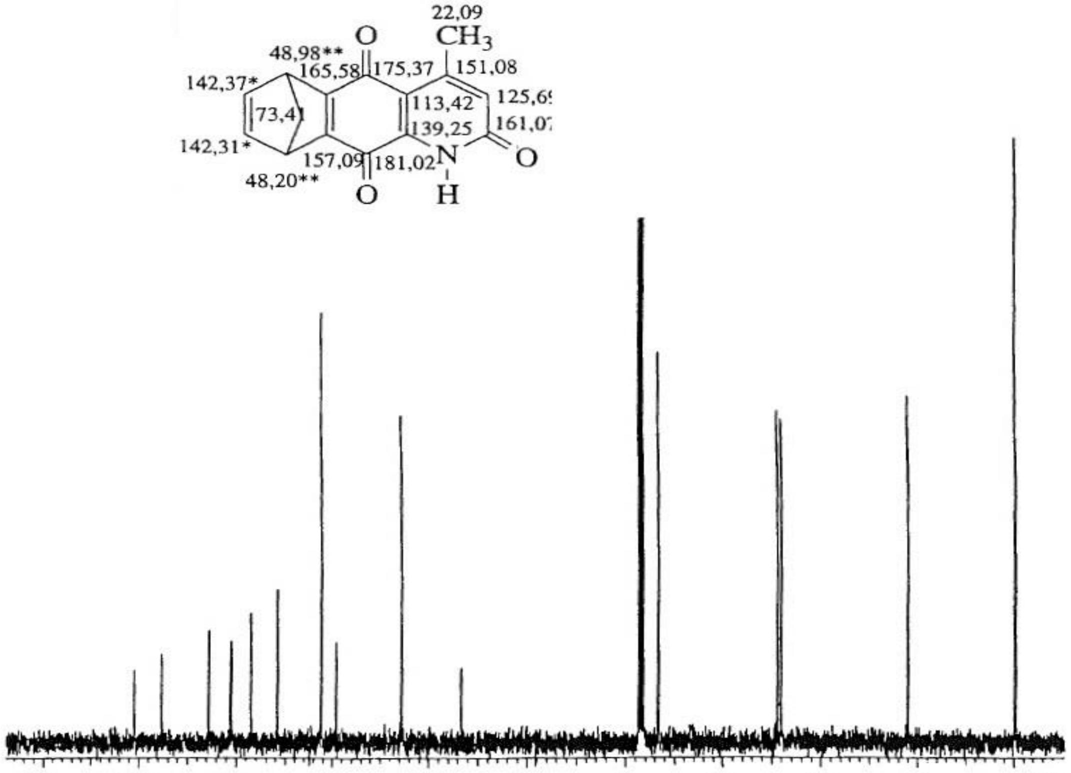
22,09
CH3
48,98**
142,37*
165,58
175,37
151,08
113,42
125,6
73,41
139,25
161,0
142,31*
157,09
181,02
48,20**
200
180
160
140
120
100
80
60
40
20
ppm
